# Life Course Trajectories of Body Mass Index and Risk of Cancer in Adulthood: Systematic Review and Meta‐Analysis

**DOI:** 10.1111/obr.70114

**Published:** 2026-03-05

**Authors:** Samira Behboudi‐Gandevani, Tommy Haugan, Nayla Cristina Do Vale Moreira, Ellen Christin Arntzen, Moira Strand Hutchinson, Melanie Nichols, Razieh Bidhendi‐Yarandi

**Affiliations:** ^1^ Faculty of Nursing and Health Sciences Nord University Bodø Norway; ^2^ Faculty of Nursing and Health Sciences Nord University Levanger Norway; ^3^ Valnesfjord Centre for Health Sports and Rehabilitation Fauske Norway; ^4^ Global Centre for Preventive Health and Nutrition, Institute for Health Transformation, Faculty of Health Deakin University Geelong Victoria Australia; ^5^ Department of Biostatistics and Epidemiology, School of Social Health University of Social Welfare and Rehabilitation Sciences Tehran Iran; ^6^ Social Determinants of Health Research Center University of Social Welfare and Rehabilitation Sciences Tehran Iran

**Keywords:** body mass index, cancer risk, life course, trajectory

## Abstract

**Objective:**

This systematic review and meta‐analysis aimed to identify common life‐course body mass index (BMI) trajectories (childhood/adulthood to adulthood) and their impact on risk of cancer overall and cancer at different sites in adulthood.

**Methods:**

Observational studies were identified that assessed the association of BMI trajectories with cancer risks from databases published in English. The pooled effect sizes were estimated using a random‐effects model.

**Findings:**

A total of 24 eligible studies were included in the meta‐analysis. Transitioning from normal weight to obesity was significantly associated with an increased risk of colorectal cancer (effect size [ES] = 1.170, 95% CI: 1.096, 1.245), pancreatic cancer (ES = 1.337, 95% CI: 1.247, 1.426), kidney cancer (ES = 2.122, 95% CI: 1.619, 2.624), and liver cancer (ES = 1.777, 95% CI: 1.261, 2.293), compared to those in the stable normal weight trajectory. No significant difference in the risk of breast or prostate cancer was observed when comparing various BMI trajectories to a stable normal BMI trajectory.

**Conclusion:**

The findings suggest that lifetime BMI trajectories influence cancer risk, with a transition from normal BMI to higher levels associated with an increased risk of cancer. The impact varied by trajectory and cancer type. Further studies are needed to confirm these findings.

## 1 Introduction

The global burden of cancer continues to rise, with an increasing incidence observed across the world [1]. Currently, the lifetime risk of developing cancer is approximately one in four, affecting men and women at similar rates [2]. By 2050, over 35 million new cancer cases are expected, reflecting a 77% increase from the estimated 20 million cases in 2022 [1, 3]. Given these trends, understanding the relative contribution of modifiable risk factors to various cancer types is essential for the development of effective cancer prevention strategies at local and global levels.

Obesity has tripled in prevalence over the past several decades and is now an established risk factor for multiple cancers, including colorectal, breast, endometrial, and pancreatic cancers, among others [4, 5]. However, emerging evidence suggests that the lifetime risk of excess body weight on various cancers may depend on both the severity and duration of overweight or obesity throughout life. Investigating the health effects of high BMI requires a life‐course research approach, which enables examination of how different body mass index (BMI) patterns over the lifespan may influence cancer risk in adulthood. Recent epidemiological studies have employed advanced methods to explore the life course trajectories of BMI and their effects on health outcomes, specifically on different cancers [6, 7, 8, 9]. Trajectory analysis can provide further evidence on the impact of an exposure throughout life, helping to identify critical time points and subgroups to inform prevention and control strategies [10].

Nevertheless, BMI trajectories are often inconsistently defined, and their effects on cancer risk remain debated across studies [8, 9, 11, 12, 13, 14]. This systematic review and meta‐analysis therefore aimed to summarize the current state of the literature, identify common life‐course BMI trajectories, and assess their impact on overall and site‐specific cancer risk in adulthood.

## 2 Methods

This systematic review and meta‐analysis was conducted in accordance with the 2020 Preferred Reporting Items for Systematic Reviews and Meta‐Analyses (PRISMA) statement [15]. The protocol was registered in the International Prospective Register of Systematic Reviews (PROSPERO) under the registration number CRD42024603179. An Institutional Review Board approval was not required, as the study utilized previously published data that contained no identifiable information.

### 2.1 Search Strategy

The following PICO (Population, Exposure, Comparison, Outcome) elements were applied for this systematic review: (P) Individuals whose BMI trajectories spanning from childhood/adulthood to adulthood; (I) distinct BMI trajectories across the lifespan; (C) Stable normal weight trajectory; (O) Incidence/diagnosis of each cancer or group of cancers, as defined by the included studies, measured by pooled effect sizes (ES) and 95% confidence intervals (CI).

Electronic databases of PubMed (with MEDLINE), EMBASE, Scopus, and Web of Science were searched from 2000 to Aug 2024. Additionally, we manually searched reference lists of relevant articles, performing both backward and forward citation searches. A combination of synonyms and relevant terms was employed to maximize the sensitivity of the search across electronic databases used to identify relevant studies and titles, abstracts, and keywords. The final search strategy was developed in consultation with a librarian at Nord University.

The following keywords were used: (“body mass index” OR bmi OR “body weight*” OR “overweight*” OR “body weight gain*” OR “weight gain*” or obes* OR anthropometr* OR “Adiposity”) AND (trajector* OR “life course*” OR “life‐long” OR “life‐course” OR “lifelong” OR “life course trajector*” OR “life course trajector*” OR “growth mixture model*” OR “group‐based trajectory model*” OR “latent class model*” OR “Growth Patterns” OR class* OR group* OR cluster* OR path* OR pattern* OR profile* OR longitudinal) AND (cancer OR “Neoplasm” OR Carcinoma* OR malignan* OR oncolog* OR “Tumor”).

### 2.2 Selection of Studies

Inclusion and exclusion criteria were defined prior to the literature search. To be eligible for inclusion, studies needed to meet the following criteria: observational studies that assessed BMI trajectories across the life course, including those from childhood to adulthood (childhood to over 18 years) or from adulthood to later adulthood (ages 18 and older), and examined their relationship with cancer occurring in adulthood (over 18 years). BMI measurements needed to be assessed at a minimum of three time points to allow for trajectory identification. Studies that used self‐reported, recalled, or measured BMI were considered eligible. There were no restrictions on cancer definitions; studies were included if cancer outcomes were assessed via self‐report or objective measures, in accordance with national or international criteria. Only peer‐reviewed studies published in English were considered for inclusion. All studies that focused on disease‐specific populations, cancer survivors, as well as gray literature and nonprimary research articles, commentaries, editorials, and reviews were excluded. Brief communications were eligible if they provided sufficient data relevant to our study.

### 2.3 Screening and Data Extraction

Selection of eligible studies and data extraction was performed independently by two of the reviewers (SB‐G and RBY); any disagreement was resolved through discussion. General information extracted from each study included the first author, year of publication, country, and study setting. Additional details gathered comprised study design, sample size for analysis, duration of follow‐up, life period assessed, and the number of time points. The methods used for BMI assessment were also documented. Furthermore, trajectory‐specific data were collected, which included statistical modeling methods, the number and names of identified trajectories, the nature of each trajectory, and the proportion of participants within each trajectory, adjusted variables, and data on the association between BMI trajectories and both composite cancer outcomes and specific cancer types. In instances of missing data or ambiguities within any part of the study, the authors were contacted via email to request additional information.

### 2.4 Quality Assessment

The quality of the included studies was evaluated using the Newcastle–Ottawa Scale (NOS) for observational studies [16]. This tool consists of eight criteria divided across three categories: selection of study population, comparability between groups, and determination of outcomes or exposures. The NOS uses a semiquantitative approach, awarding stars based on the methodological rigor of the study. A maximum of four stars can be given for population selection, two stars for group comparability, and three stars for outcome assessment. The total NOS score ranges from zero to nine stars, with studies rated as good quality (seven to nine stars), fair quality (four to six stars), or poor quality (zero to three stars). One reviewer conducted the primary quality assessment, and 15% of the studies were double‐checked by a second reviewer to ensure consistency.

### 2.5 Synthesis of Results/Data Analysis

A narrative synthesis was performed for the results of studies included in the meta‐analysis. To generate a consistent effect measure across studies that are included in the meta‐analysis, relative risks (RR) and hazard ratios (HR) were converted to odds ratios (ORs) using standard methods [17, 18].

Certain trajectories were consistently reported across multiple studies. Based on the characteristics of consistently identified BMI trajectories, the following four classifications of trajectories were defined for meta‐analysis: (1) “stable normal weight” trajectory (normal BMI throughout the life course/follow‐up [childhood/adulthood to adulthood]), as the reference group; (2) “normal to overweight trajectory (normal BMI during childhood/adulthood and overweight in adulthood); (3) “normal to obesity trajectory (normal BMI during childhood/adulthood and obesity in adulthood); and (4) “overweight to obesity” trajectory (overweight BMI during childhood/adulthood and obesity in adulthood). All included studies used standard BMI cutoffs as defined by the World Health Organization (WHO). Underweight was defined as a BMI below 18.5 kg/m^2^, normal weight as a BMI between 18.5 and 24.9, overweight as a BMI between 25.0 and 29.9, and obesity as a BMI of 30.0 or higher.

For the pooled analysis, BMI trajectories were selected based on their frequency, consistency, and conceptual similarity across the included studies.

Heterogeneity was evaluated using the Chi‐square test and I‐square index; the random effect model was applied in case of significant heterogeneity. Publication bias was assessed using Harbord and the Begg's tests. In cases where significant publication bias was evident, the trim and fill method was used to address this. Forest plots for each outcome and by subgroup of the trajectories of BMI were also illustrated.

Most of the included studies applied the Latent Class Growth Mixture Modeling (LCGMM) approach to estimate trajectories of BMI, considering age as the time variable. LCGMM is a semiparametric statistical technique used to identify distinct subgroups within a population based on their growth trajectories following a distinct pattern of change over the time variable of interest. It combines elements of growth modeling and latent class analysis, allowing researchers to capture heterogeneity in developmental patterns [19, 20].

Raw data were extracted from the included studies and their Supporting Information files to estimate the crude ES and standard errors. Adjusted effect sizes (95% CI) were extracted as well. Since most of the outcomes reported could be considered as rare events, we treated OR, RR, and HR as equivalent [17]. Trajectories were defined and classified based on the models and the variation of BMI reported by studies. Sensitivity analyses were performed sequentially excluding each study from the pooled analysis to evaluate the influence of individual studies on the overall estimates and to assess the stability and robustness of the meta‐analytic results. The STATA software package (version 14; STATA Inc., College Station, TX, USA) was used to conduct statistical analysis. The *p*‐value < 0.05 was set as statistically significant.

## 3 Results

### 3.1 Study Selection and Characteristics Included Studies and Quality Appraisal

**FIGURE 1:**
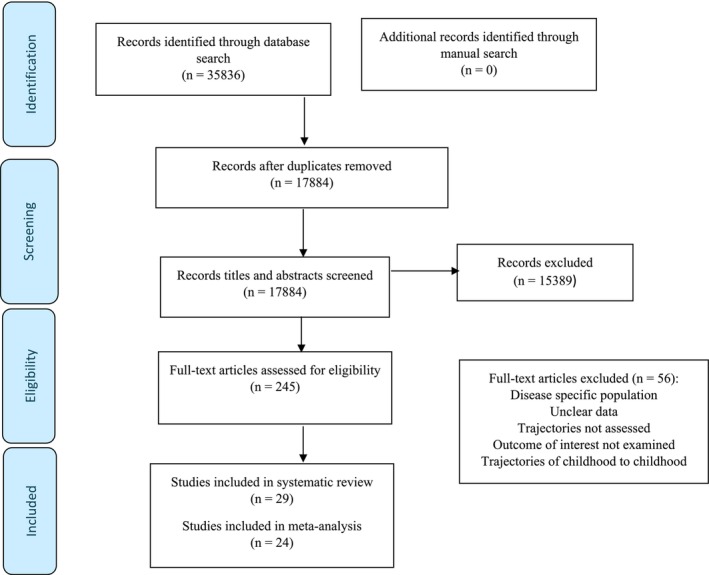
Study flowchart.

Figure 1 illustrates the flow diagram of the study selection procedure. The literature search of electronic databases resulted in a total of 35,836 records. No additional studies were identified through manual research or contact with authors. We excluded duplicates that appeared in multiple databases (*n* = 17,952). Subsequently, we evaluated the titles and abstracts of the remaining articles, resulting in 245 articles being selected for further evaluation. After screening the full texts of these articles, a total of 29 studies [8, 9, 11, 12, 13, 14, 21, 22, 23, 24, 25, 26, 27, 28, 29, 30, 31, 32, 33, 34, 35, 36, 37, 38, 39, 40, 41, 42, 43] met all inclusion criteria and were included in the systematic review. Among these studies, a total of five studies [22, 24, 28, 35, 43] were excluded from the final meta‐analysis; four studies [22, 24, 28, 35] were excluded because they used time to follow‐up as a variable to develop trajectories, which does not align with our inclusion criteria for BMI trajectory modeling. One study [43] was excluded as it analyzed BMI growth trajectories rather than distinct life‐course BMI trajectories, which were required for inclusion in the meta‐analysis. The characteristics of the included studies are presented in Table 1.

**TABLE 1:** Characteristics of studies included on life‐course trajectories of body mass index and cancer risks in adulthood.

ID	Country	Study design	Sample size for analysis	Sex, female (%)	Period of life (age range, years) for trajectories	Number of assessment points, and method of assessment	Outcomes	Mean follow‐up time	Trajectories identified (number and names with % participant)	Outcomes with OR/RR/HR (95% CI)
Abdel‐Rahman O. 2019	USA	Prospective cohort (PLCO trial)	145,544	50.7%	20–74 years	Three assessments, Self‐reported BMI at 20 and 50 years, measured BMI at enrollment	Lung cancer	Mean follow‐up of the entire study cohort is 4106.95 days (11.25 years); SD: 849.639	Five trajectories: 1. Normal to normal (38.7%) 2. Normal to overweight (30.7%) 3. Normal to obese (6.8%) 4. Overweight to obese (5.5%) 5. Other (18.2%)	**Lung cancer risk:**—Normal to normal: Ref—Normal to overweight: HR 0.662 (0.560, 0.782) ‐ Normal to obese: HR 0.798 (0.734, 0.867) ‐ Overweight to obese: HR 0.687 (0.579, 0.815) ‐ Other: No information presented
Arjani S, et al. 2022	USA	Prospective cohort (NIH–AARP Diet and Health Study)	269,480 (162,735 males, 106,745 females)	39.6%	18–71 years	Four assessments Self‐reported BMI at ages 18, 35, 50, and measured at baseline (50–71 years)	Pancreatic ductal adenocarcinoma	Follow‐up from 1995 to 2011, 15.2 years of follow‐up,	Four trajectories: 1. Normal‐weight maintainer (39.5%) 2. Normal‐to‐overweight (39.8%) 3. Normal‐to‐obese Class I (17.5%) 4. Overweight‐to‐obese Class III (3.2%)	**Pancreatic ductal adenocarcinoma risk:** **Overall population** ‐Normal‐weight maintainer: Ref ‐Normal‐to‐overweight: HR 1.15 (1.06, 1.25) ‐Normal‐to‐obese Class I: HR 1.39 (1.25, 1.54) ‐Overweight‐to‐obese Class III: HR 1.48 (1.18, 1.87) **Males** ‐Normal‐weight maintainers: Reference (HR = 1) ‐Normal‐to‐overweight: HR = 1.16 (95% CI: 1.05–1.29) ‐Normal‐to‐obese Class I: HR = 1.45 (95% CI: 1.28–1.64) ‐Overweight‐to‐obese Class III: HR = 1.76 (95% CI: 1.30–2.38) **Females** ‐Normal‐weight maintainers: Reference ‐Normal‐to‐overweight: HR = 1.17 (95% CI: 1.02–1.34) ‐Normal‐to‐obese Class I: HR = 1.28 (95% CI: 1.07–1.53) ‐Overweight‐to‐obese Class III: HR = 1.22 (95% CI: 0.85–1.74)
Busund M, et al. 2023	Norway	Prospective cohort study (Norwegian Women and Cancer Study)	148,866	100%	Adulthood, starting from age 18 until postmenopause	Up to four BMI assessment points (self‐reported at age 18, and during three follow‐up questionnaires)	Postmenopausal breast cancer subtypes	14.9 years	Five trajectories: 1. Normal‐stable: 43.5% 2. Normal‐overweight: 40.3% 3. Normal‐obesity: 12.8% 4. Overweight‐obesity: 2.5% 5. Obesity‐decrease: 0.8%	**Postmenopausal breast cancer overall** Normal‐stable: Ref Normal‐overweight: HR 1.02 (0.96–1.07) Normal‐obesity: HR 1.01 (0.94–1.09) Overweight‐obesity: HR 0.88 (0.75–1.05) Obesity‐decrease: HR 0.54 (0.33–0.90) **Luminal A‐like breast cancer**: Normal‐stable: Ref Normal‐overweight: HR 1.09 (1.01–1.18) Normal‐obesity: HR 1.20 (1.08–1.33) Overweight‐obesity: HR 1.05 (0.84–1.33) Obesity‐decrease: HR 0.54 (0.33–0.90)
										**Luminal B‐like breast cancer**: Normal‐stable: Ref Normal‐overweight: 0.95 (0.84–1.06) Normal‐obesity: HR 0.94 (0.78–1.12) Overweight‐obesity: 0.64 (0.41–1.00) Obesity‐decrease: HR 0.67 (0.33–1.35) **HER2‐enriched breast cancer**: Normal‐stable: Ref Normal‐overweight: 0.96 (0.73–1.27) Normal‐obesity: HR 0.82 (0.53–1.28) Overweight‐obesity: 1.00 (0.44–2.30) Obesity‐decrease: HR 0.54 (0.08–3.88) **TNBC breast cancer**: Normal‐stable: Ref Normal‐overweight: HR 0.91 (0.74–1.11) Normal‐obesity: HR 0.96 (0.72–1.30) Overweight‐obesity: HR 0.78 (0.40–1.53) Obesity‐decrease: HR 0.76 (0.24–2.37)
Chiu PW, et al. 2023	Taiwan	Retrospective cohort study	89,886	53.3%	Adults aged 40 + years	Seven assessment points using measured BMI data at health check‐ups	Cause‐specific mortality	16.8 years	Four trajectories: 1. Obesity, stable (4.4%–7.3%) 2. Overweight, stable (21.9%–36.1%) 3. Midnormal weight, stable (42.7%–47.2%) 4. Low‐normal weight, stable (13.9%–29.7%)	**Cancer‐related mortality**: **Men aged over 60**: Obesity: HR = 1.34 (1.09–1.64) Overweight: No significant Midnormal weight: Ref Low‐normal weight: No significant **Men aged 40–60:** Obesity: No significant Overweight: No significant Mid‐normal weight: Ref Low‐normal weight: No significant **Women aged over 60**: Obesity: HR = 0.75 (0.60–0.93) Overweight: No significant Midnormal weight: Ref Low‐normal weight: No significant **Women aged 40–60:** Obesity: No significant Overweight: No significant Mid‐normal weight: Ref Low‐normal weight: No significant
Dalmartello M, et al. 2022	Italy	Pooled analysis of two case–control studies	458 endometrial cancer cases and 782 controls	100%	Adult lifetime (starting from ages 20–29 years)	Six Self‐reported BMI assessments: (20–29, 30–39, 40–49, 50–59, 60–69, 70–74 years).	Endometrial cancer risk	over six decades	Five trajectories: 1. Underweight increasing to normal weight (8.3%) 2. Normal weight‐stable (43.2) 3. Normal weight increasing to overweight (23.1%) 4. Overweight‐stable (13.7%) 5. Overweight increasing to obese (11.8%)	**Endometrial cancer risk:** Normal weight‐stable: Ref Underweight increasing to normal weight: OR 0.50 (0.28, 0.99) Normal weight increasing to overweight: No significant Overweight‐Stable: OR 1.71 (1.12, 2.59) Overweight Increasing to Obese: OR 2.03 (1.31, 3.13)
De Rubeis V, et al. 2019	Canada	Population‐based case–control study	Cases: 310 Controls: 1258	NM	From Adolescence, 20s, 30–40s to 50–60s	Four assessment points (Adolescence, 20s, 30–40s, 50–60s) Self‐reported height and weight at each timepoint	Pancreatic cancer	NM	Five trajectories: 1. Stable‐normal weight (38.9%) 2. Progressively overweight (42.2%) 3. Persistent overweight (12.6%) 4. Progressive obesity (4.2%) 5. Persistent obesity (2.1%)	**Pancreatic cancer risk** ^a^ **:** Stable‐normal weight: Ref Progressively overweight: OR 1.15 (0.84, 1.58) Persistent overweight: OR 1.63 (1.05, 2.53) Progressive obesity: OR 1.51 (0.77, 2.99) Persistent Obesity: OR 0.22 (0.03, 1.70)
Gray LA, et al. 2022	UK	Cohort study	9206	Approximately 54%	50 years and older	5 points (1998–2000, 2002–2003, 2006–2007, 2010–2011, 2012–2013) Method: BMI measured by nurse visits	Composite outcome of cancer	15 years (1998–2013)	Four trajectories: 1. Stable Overweight: 85.3% 2. Increasing BMI: 7.2% 3. Elevated BMI: 4.1% 4. Decreasing BMI: 3.4%	**Cancer risk** ^a^: 1. Stable Overweight: Ref 2. Increasing BMI: HR: 0.873 (0.568–1.343) 3. Elevated BMI: HR: 1.308 (0.848–2.017) 4. Decreasing BMI: HR: 1.080 (0.636–1.835)
Hoyt M, et al. 2022	USA	Cohort study within the Prostate, Lung, Colorectal, and Ovarian (PLCO) Cancer Screening Trial.	145,489 participants (with 696 incident cases of pancreatic cancer)	Approximately 26.8%	Ages 20, 50, and 55–74 years	Three assessment points (ages 20, 50, and baseline at 55–74 years), self‐reported weight and height.	Pancreatic cancer risk	12 years	Four trajectories: Steady normal weight (37.9%) Normal weight to overweight (45.4%) Normal weight to obesity (14.2%) Overweight to obesity (2.5%)	**Total Pancreatic cancer risk** ^a^ **:** Steady normal weight: Ref Normal weight to overweight: HR = 1.06 (0.90–1.26) Normal weight to obesity: HR = 1.04 (1.81–1.34) Overweight to obesity: HR = 1.43 (0.87–2.34) **Early‐stage pancreatic cancer risk** ^a^ **:** Steady normal weight: Ref Normal weight to overweight: HR = 1.01 (0.77–1.31) Normal weight to obesity: HR = 1.18 (0.82–1.71) Overweight to obesity: HR = 1.01 (1.41–2.48)
Song M, et al. 2016	USA	Cohort study (Nurses' Health Study and Health Professionals Follow‐up Study).	73,581 women and 32,632 men	69%	5–60 years	At least 5 time points (ages 5, 10, 20, 30, 40, and measured at 50 and 60), assessed using self‐reported body shape and BMI measurements.	Total and obesity‐related cancers including esophagus, colorectal, pancreas, breast (postmenopausal), endometrium, ovaries, prostate (advanced), kidney, liver, gallbladder.	Approximately 10 years.	Five trajectories: Lean‐stable (16%) Lean‐moderate increase (22%) Lean‐marked increase (21%) Medium‐stable (27%) Heavy‐stable/increase (14%)	**Women** **Total cancer risk:** Lean‐stable: Ref Lean‐moderate increase: RR 1.06 (1.00–1.13) Lean‐marked increase: RR 1.19 (1.09–1.24) Medium‐stable: RR 1.04 (0.98–1.10) Heavy‐stable/increase: RR 1.15 (1.07–1.23) **Obesity‐related cancer risk** ^b^: Lean‐stable: Ref Lean‐moderate increase: RR 1.17 (1.08–1.30) Lean‐marked increase: RR 1.39 (1.27–1.50) Medium‐stable: RR 1.03 (0.95–1.12) Heavy‐stable/increase: RR 1.28 (1.17–1.41) **Colorectal cancer risk:** Lean‐stable: Ref Lean‐moderate increase: RR 0.97 (0.80–1.17) Lean‐marked increase: RR 1.22 (1.00–1.49) Medium‐stable: RR 1.02 (0.85–1.22) Heavy‐stable/increase: RR 1.40 (1.13–1.74) **Esophageal adenocarcinoma risk:** Lean‐stable: Ref Lean‐moderate increase: RR 1.02 (0.29–3.63) Lean‐marked increase: RR 2.56 (0.82–8.03) Medium‐stable: RR 1.04 (0.30–3.57) Heavy‐stable/increase: RR 2.19 (0.63–7.70) **Pancreatic cancer risk:** Lean‐stable: Ref Lean‐moderate increase: RR 1.18 (0.82–1.69) Lean‐marked increase: RR 1.36 (0.93–1.98) Medium‐stable: RR 1.15 (0.81–1.63) Heavy‐stable/increase: RR 1.39 (0.91–2.12) **Kidney cancer risk:** Lean‐stable: Reference group Lean‐moderate increase: RR 1.26 (0.78–2.04) Lean‐marked increase: RR 1.89 (1.19–3.03) Medium‐stable: RR 1.05 (0.65–1.69) Heavy‐stable/increase: RR 1.92 (1.15–3.21)
										**Postmenopausal breast cancer risk:** Lean‐stable: Ref Lean‐moderate increase: RR 1.30 (1.17–1.45) Lean‐marked increase: RR 1.41 (1.26–1.58) Medium‐stable: RR 1.05 (0.94–1.17) Heavy‐stable/increase: RR 1.11 (0.97–1.28) **Endometrial cancer risk:** Lean‐stable: Reference group Lean‐moderate increase: RR 0.99 (0.75–1.29) Lean‐marked increase: RR 1.57 (1.21–2.03) Medium‐stable: RR 0.94 (0.73–1.22) Heavy‐stable/increase: RR 2.08 (1.59–2.73) **Ovarian cancer risk:** Lean‐stable: Reference group Lean‐moderate increase: RR 0.88 (0.66–1.16) Lean‐marked increase: RR 0.93 (0.70–1.25) Medium‐stable: RR 0.88 (0.67–1.15) Heavy‐stable/increase: RR 0.84 (0.59–1.19) **Men** **total cancer risk:** Lean‐stable: Ref Lean‐moderate increase: RR 1.06 (0.96–1.16) Lean‐marked increase: RR 1.07 (0.98–1.16) Medium‐stable: RR 1.04 (0.94–1.15) Heavy‐stable/increase: RR 1.22 (1.09–1.36) **Obesity‐related cancer risk** ^c^: Lean‐stable: Ref Lean‐moderate increase: RR 1.17 (1.00–1.37) Lean‐marked increase: RR 1.09 (0.95–1.26) Medium‐stable: RR 1.13 (0.95–1.34) Heavy‐stable/increase: RR 1.17 (0.97–1.42) **Colorectal cancer risk**: Lean‐stable: Ref Lean‐moderate increase: RR 1.36 (1.03–1.80) Lean‐marked increase: RR 1.23 (0.95–1.60) Medium‐stable: RR 1.26 (0.92–1.72) Heavy‐stable/increase: RR 1.47 (1.05–2.05)
										**Esophageal adenocarcinoma risk:** Lean‐stable: Ref Lean‐moderate increase: RR 1.90 (0.67–5.34) Lean‐marked increase: RR 2.09 (0.80–5.48) Medium‐stable: RR 1.53 (0.48–4.84) Heavy‐stable/increase: RR 3.01 (1.04–9.13) **Pancreatic cancer risk:** Lean‐stable: Ref Lean‐moderate increase: RR 0.85 (0.54–1.35) Lean‐marked increase: RR 1.20 (0.81–1.78) Medium‐stable: RR 1.12 (0.70–1.80) Heavy‐stable/increase: RR 1.50 (0.92–2.46) **Kidney cancer risk:** Lean‐stable: Ref Lean‐moderate increase: RR 1.05 (0.67–1.64) Lean‐marked increase: RR 0.94 (0.63–1.43) Medium‐stable: RR 1.07 (0.66–1.74) Heavy‐stable/increase: RR 0.93 (0.53–1.64) **Advanced prostate risk:** Lean‐stable: Ref Lean‐moderate increase: RR 1.16 (0.91–1.47) Lean‐marked increase: RR 0.97 (0.78–1.21) Medium‐stable: RR 1.00 (0.76–1.32) Heavy‐stable/increase: RR 0.67 (0.47–0.95)
Kelly SP, et al. 2019	USA	Prospective cohort study	153,730 men	0%	Ages 18, 35, and 50 years, plus study entry (mean age 63 years)	Four time points (ages 18, 35, 50, and study entry), BMI assessed via self‐report	Mortality of fatal prostate cancer and based on smoking status	15.1 years	**Five trajectories:** Stable normal BMI: 45% Normal BMI to overweight: 39% Normal BMI to obese: 9% Stable overweight: 6% Overweight to obese: 2%	**Fatal prostate cancer risk** ^a^ **:** Stable normal: Ref Normal to overweight: HR = 1.13 (0.95, 1.34) Normal to Obese: HR = 1.16 (0.84, 1.58) Stable Overweight: HR = 1.11 (0.77, 1.61) Overweight to Obese: HR = 0.70 (0.29, 1.70)
Kelly SP, et al. 2016	USA	Prospective cohort study	69,873 men	0%	20 to baseline (mean age 63 years)	3 assessment (ages 20, 50, and baseline), Self‐reported BMI recalled at ages 20 and 50, measured at baseline	Incidence and mortality of prostate cancer	11.5 years	**Five trajectories:** Normal to overweight: 47% Normal to obese: 7% Stable normal: 33% Stable overweight: 10% Overweight to obese: 3%	**Total prostate cancer risk** ^a^ **:** Normal to overweight: HR = 0.96 (0.91–1.01) Normal to Obese: HR = 0.98 (0.89–1.08) Stable Normal: Ref Stable Overweight: HR = 0.91 (0.83–0.99) Overweight to obese: HR = 0.79 (0.67–0.93) **Fatal prostate cancer risk** ^a^ **:** Normal to overweight: HR = 0.99 (0.74–1.32) Normal to obese: HR = 1.95 (1.21–3.12) Stable normal: Ref Stable overweight: HR = 1.51 (0.96–2.36) Overweight to obese: HR = 2.65 (1.35–5.18) **Nonaggressive prostate cancer risk** ^a^ **:** Stable normal: Ref Normal to overweight: HR = 0.97 (0.88–1.07) Normal to obese: HR = 0.92 (0.74–1.14) Stable overweight: HR = 0.86 (0.71–1.05) Overweight to obese: HR = 0.73 (0.51–1.05) **Aggressive prostate cancer risk** ^a^ **:** Stable normal: Ref Normal to overweight: HR = 0.99 (0.74–1.32) Normal to obese: HR = 1.95 (1.21–3.12) Stable overweight: HR = 1.51 (0.96–2.36) Overweight to obese: HR = 2.65 (1.35–5.18)
Kuchibhatla MN, et al. 2013	USA	Longitudinal study	3861	65%	65–105 years	4 points (baseline, 3 years, 6 years, and 10 years) using self‐reported weight and height, with some measured data at the third interview	Cancer (excluding skin cancer)	10 years	**Three trajectories:** Normal weight: 27.6% Overweight: 65.1% Obese: 7.3%	**Cancer risk (excluding skin)** ^a^ **:** Normal weight: Reference Overweight: HR = 1.02 (0.78, 1.32) Obese: HR = 1.28 (0.80, 2.06)
Lavalette C, et al. 2022	France	Population‐based case–control study	781 cases and 829 controls (total 1610)	0%	20–2 years before diagnosis/reference date	5 points measures over a lifetime (self‐reported weight every decade)	Prostate cancer risk	Not explicitly stated; cases diagnosed in 2012–2013	**Five trajectories:** Stable Normal BMI (36.3%) Normal to overweight (28.6%) Growing Overweight (23.6%) Normal to obese (7.6%) Overweight to obese (3.9%)	**All Prostate cancer risk** ^a^ **:** Stable Normal BMI: Ref Normal to Overweight: OR = 1.04 (0.80–1.35) Growing Overweight: OR = 1.03 (0.78–1.36) Normal to obese: OR = 0.98 (0.64–1.49) Overweight to obese: OR = 1.05 (0.60–1.86) **Low and intermediate prostate cancer risk** ^a^ Stable normal BMI: Ref Normal to overweight: OR = 1 1.03 (0.78–1.37) Growing overweight: OR = 0.96 (0.71–1.30) Normal to obese: OR = 1.05 (0.67–1.63) Overweight to obese: OR = 0.81 (0.42–1.54) **Agresssive prostate cancer risk** ^a^ Stable normal BMI: Ref Normal to overweight: OR = 1.11 (0.71–1.74) Growing Overweight: OR = 1.27 (0.80–2.00) Normal to obese: OR = 0.81 (0.36–1.81) Overweight to Obese: OR = 2.16 (1.00–4.66)
Luo L, et al. 2020	China	Prospective cohort (PLCO cancer screening trial)	39,824	48.5%	BMI was measured at ages 20, 30, 40, and 50.	Four assessment points (ages 20, 30, 40, and 50), using self‐reported height and weight to calculate BMI.	Colorectal adenomas (premalignant condition)	12.4 years	**Four trajectories:** Normal weight maintained: 41.2% Normal to overweight: 44.1% Normal to obesity: 12.9% Overweight to obesity: 1.9%	**Colorectal adenomas risk:** Normal weight maintained: Ref Normal to overweight: 1.04 (0.98, 1.11) Normal to obesity: 1.15 (1.06, 1.25) Overweight to obesity: 1.33 (1.11, 1.61)
Pedersen DC, et al. 2023	Denmark	Cohort study	6698	100%	Childhood (ages 6–15 years) and adulthood (ages 20, 30, 40, 50, 50–64 years)	At least 6 BMI measures across the life course (ages 6–15 in childhood; ages 20, 30, 40, 50, and post‐50 in adulthood), Combination of measured BMI in childhood (from school health records) and self‐reported BMI in adulthood	Post‐menopausal breast cancer by estrogen receptor (ER) status (ER‐positive and ER‐negative)	23 years	**Six trajectories:** Average BMI (low or stable) gain across life (54.1%) High BMI increase in childhood and adolescence, stabilizing in adulthood (14.4%) Low BMI gain in childhood, followed by a steep increase in adulthood (17.1%) Continued large BMI gain throughout life (2.7%) Steep BMI increase in childhood, stabilizing at the overweight level in adulthood (10.5%) Steep gain in BMI in childhood and adolescence, continuing into early adulthood (BMI > 35 kg/m^2^) (1.2%)	**Overall postmenopausal breast cancer risk** ^a^ **:** Average BMI gain across life: (Reference) High BMI increase in childhood, stabilizing in adulthood: HR = 0.67 (0.48–0.93) Low BMI gain in childhood, steep increase in adulthood): HR = 1.20 (0.94–1.54) Continued large BMI gain throughout life): HR = 0.99 (0.54–1.81) Steep BMI increase in childhood, stabilizing at overweight level in adulthood): HR = 0.77 (0.55–1.08) Steep BMI gain in childhood, continuing into adulthood with BMI > 35 kg/m^2^): HR = 0.66 (0.25–1.75) **ER‐positive tumors risk** ^a^ **:** Average BMI gain across life): (Reference) High BMI increase in childhood, stabilizing in adulthood): HR = 0.67 (0.47–0.95) Low BMI gain in childhood, steep increase in adulthood): HR = 1.28 (0.98–1.67) Continued large BMI gain throughout life): HR = 0.99 (0.54–1.81) Steep BMI increase in childhood, stabilizing at overweight level in adulthood): HR = 0.68 (0.46–1.01) Steep BMI gain in childhood, continuing into adulthood with BMI > 35 kg/m^2^): HR = 0.66 (0.25–1.75) **ER‐negative tumors risk** ^a^ **:** Average BMI gain across life): (Reference) High BMI increase in childhood, stabilizing in adulthood): HR = 0.95 (0.41–2.19) Low BMI gain in childhood, steep increase in adulthood): HR = 0.82 (0.36–1.88) Continued large BMI gain throughout life): HR = 1.28 (0.33–5.00) Steep BMI increase in childhood, stabilizing at overweight level in adulthood): HR = 1.02 (0.45–2.34) Steep BMI gain in childhood, continuing into adulthood with BMI > 35 kg/m^2^): HR = 0.80 (0.10–6.39)
Petrick JL, et al. 2017	USA	Pooled analysis of two prospective cohort studies (NIH‐AARP Diet and Health Study, and PLCO Cancer Screening Trial)	409,796 individuals (633 esophageal adenocarcinoma (EA) cases, 415 gastric cardia adenocarcinoma (GCA) cases)	NM	Ages 20, 50, and baseline (age ≥ 50 years)	3 points—ages 20, 50, and baseline (age 50–71) Self‐reported height and weight at all points (age 20, age 50, and baseline)	Incidence of esophageal adenocarcinoma (EA) and gastric cardia adenocarcinoma (GCA)	Participants were followed up to December 31, 2011 (NIH‐AARP) and December 31, 2009 (PLCO), with a mean follow‐up time of approximately 10–15 years	**Four trajectories:** Normal BMI to normal BMI (42.8%) Normal BMI to overweight (43.4%) Normal BMI to obese (11.7%) Overweight to obese (2.0%)	**Esophageal adenocarcinoma risk** ^a^: Normal BMI to normal BMI: Reference Normal BMI to overweight: HR = 1.39 (1.15–1.69) Normal BMI to obese: HR = 2.42 (1.90–3.08) Overweight to obese: HR = 2.90 (1.67–5.04) **Gastric cardia adenocarcinoma risk**: Normal BMI to normal BMI: Reference Normal BMI to overweight: HR = 1.42 (1.13–1.79) Normal BMI to obese: HR = 1.69 (1.22–2.35) Overweight to obese: HR = 4.07 (2.32–7.15)
Su L, et al. 2023	USA	Prospective cohort study	79,034	100%	18 years to baseliner (50–79 years)	4 points (ages 18, 35, 50, and baseline), self‐reported for ages 18, 35, 50; measured at baseline	Colorectal cancer	15.8 years	**Five trajectories:** Low normal stable (28.88%) High normal stable (22.34%) Normal to overweight (24.70%) Normal to obesity (14.92%) Borderline overweight to obesity (9.16%)	**Colorectal cancer risk** ^a^ **:** Low normal stable: ref. High normal stable: 1.14 (0.98–1.32) Normal to overweight: 1.10 (0.95–1.27) Normal to obesity: HR 1.29 (1.09, 1.53) Borderline overweight to obesity: HR 1.37 (1.13, 1.68)
Vallières E, et al. 2021	Canada	Population‐based case–control study	1933 cases and 1994 controls	0%	20, 40, 50, 60 years; adults up to 75 years old at diagnosis	Five time points assessed using interviews (ages 20, 40, 50, 60 years, and before interview)	Prostate cancer	N/A (case–control study)	**Four trajectories:** Normal stable (29.5%) Normal‐overweight (47.9%) Overweight‐Obese (20%) Obese‐increase (2.5%)	**Prostate cancer, overall risk** ^a^ **:** Normal stable: Ref Normal‐overweight: OR: 0.87 (0.74–1.03) Overweight‐obese: OR: 0.70 (0.57–0.86) Obese‐increase: OR: 0.39 (0.24–0.62) **Prostate cancer, low‐grade risk** ^a^ **:** Normal stable: Ref Normal‐overweight: OR: 0.90 (0.76–1.07) Overweight‐obese: OR: 0.70 (0.57–0.88) Obese‐increase: OR: 0.32 (0.18–0.55) **Prostate cancer, high‐grade risk** ^a^ **:** Normal stable: Ref Normal‐overweight: OR: 0.79 (0.61–1.03) Overweight‐Obese: OR: 0.69 (0.49–0.97) Obese‐increase: OR: 0.64 (0.32–1.28)
von Bonsdorff MB, et al. 2015	Finland	Cohort study	4943 participants	52.1%	Birth to 11 years	Average of 17 measurements on body size from birth to school age. Data on weight and height in adulthood obtained from postal questionnaires at mean age of 60 years.	Cancer mortality	10 years (2000–2010)	**Three trajectories for Men:** Increasing BMI (6.4%) Average BMI (66.7%) Average‐to‐low BMI (26.9%) **Three trajectories for women:** Increasing BMI (25.4%) Average BMI (59.0%) Low‐to‐high BMI (15.6%)	**Cancer mortality risk among men** ^a^ Increasing BMI: HR 0.73 (1.43–1.22) Average BMI: Ref Average‐to‐low BMI: HR 1.55 (1.03–2.34) **Cancer mortality risk among men** ^a^ Increasing BMI: HR 1.73 (1.00–2.98) Average BMI: Ref Low‐to‐high BMI: HR 2.70 (1.56–4.67)
Wang K, et al. 2018	USA	Observational cohort study	4857	0%	Middle‐to‐late adulthood (median age 63 at baseline)	Multiple (at least 3 in 3 different calendar years), measured in clinical settings	Prostate cancer (overall and grade‐specific)	Median of 8.0 years (IQR: 2.0, 13.0).	**Four trajectories:** Persistently normal BMI (23.4%) Normal‐to‐obese growing BMI (23.6%) Obese‐to‐normal declining BMI (44.7%) Obese growing BMI (8.3%)	**Overall Prostate cancer risk** ^a^ **:** Persistently normal BMI: Ref Normal‐to‐obese growing BMI: HR: 1.76 (1.25–2.48) Obese‐to‐normal declining BMI: HR: 1.17 (0.94–1.47) Obese growing BMI: HR: 3.72 (1.60–8.66) **Prostate cancer with Gleason score < 7 risk** ^a^ **:** Persistently normal BMI: Ref Normal‐to‐obese growing BMI: HR: 1.60 (1.11–2.30) Obese‐to‐normal declining BMI: HR: 1.19 (0.96–1.47) Obese growing BMI: HR: 3.46 (1.52–7.86) **Prostate cancer with Gleason score ≥ 7 risk** ^a^ **:** Persistently normal BMI: Ref Normal‐to‐obese growing BMI: HR: 2.88 (1.02–9.05) Obese‐to‐normal declining BMI: HR: 0.95 (0.45–2.20) Obese growing BMI: HR: 4.33 (1.52–7.74)
Watson C, et al. 2021	USA	Prospective cohort study (Prostate, Lung, Colorectal and Ovarian Cancer Screening Trial)	Subset A: 147,350 (72,513 Men; 74,837 Women) Subset B: 89,465 (42,113 Men; 47,352 Women) Subset C: 150,351 (74,106 Men; 76,245 Women)	54.5%	20–50 years (specific age recall) and 30–70 years (decade recall)	Subset A: Maximum 3 points (mean 2.99), specific age recall Subset B: Maximum 6 points (mean 5.48), decade recall Subset C: Maximum 9 points, combination of specific age and decade recall (All were Recalled, baseline was measured)	Obesity‐related cancers including colon and rectal, postmenopausal breast, endometrium, ovary, liver, gallbladder, pancreas, gastric cardia, kidney, thyroid cancers, esophageal adenocarcinoma, and meningioma	NM	**Subset A** (Specific age recall), **Men: Five trajectories**: Lean stable Lean increase Medium stable Medium increase Heavy increase **Women**: **Five trajectories**: Lean stable Lean increase Lean heavy increase Heavy increase **Subset B** (Decade recall): **Men: Seven trajectories**: Lean increase Medium increase Medium S shaped Heavy S shaped Delayed N shaped Heavy S shaped increase Double peak **Women: Six trajectories**: Lean increase Medium increase Heavy S shaped Heavy S shaped increase Delayed N shaped Double peak	**Obesity‐related cancers risk** ^a^ **:** **Subset A** (Specific age recall), **Men** Lean stable: Ref Lean increase: 1.09 (0.93–1.27) Medium stable: 1.12 (0.96–1.32) Medium increase: 1.47 (1.27–1.70) Heavy increase: 1.82 (1.42–2.34) **Women**: Lean stable: Ref Lean increase: 1.08 (1.01–1.16) Lean heavy increase: 1.23 (1.14–1.33) Heavy increase: 1.34 (1.18–1.53) **Subset B** (Decade recall)^a^: **Men:** Lean increase: Ref Medium increase: 1.45 (1.25–1.67) Medium S shaped: 1.87 (1.44–2.43) Heavy S shaped: 2.10 (1.42–3.10) Delayed N shaped: 2.07 (1.59–2.71) Heavy S shaped increase: 1.48 (0.74–2.99) Double peak: 1.94 (0.86–4.34) **Women: Six trajectories**: Lean increase: Ref Medium increase: 1.14 (1.06–1.23) Heavy S shaped: 1.30 (1.13–1.50) Heavy S shaped increase: 1.17 (1 03–1.32) Delayed N shaped: 1.61 (1.24–2.09) Double peak: 1.39 (1.02–1 89) **Subset C (Combined specific age and decade recall):** **Men:** Medium stable: Ref Medium increase: 1.12 (1.03–1.22) Medium heavy increase: 1.38 (1.16–1.63) N‐shaped: 1.96 (1.39–2.05)
									**Subset C (Combined specific age and decade recall):** **Men: Four trajectories**: Medium stable Medium increase Medium heavy increase N‐shaped **Women: Four trajectories**: Lean increase Lean heavy increase Lean extreme increase N shaped	**Women**: Lean increase: Ref Lean heavy increase: 1.14 (1.08–1.21) Lean extreme increase: 1.30 (1.18–1.43) N shaped: 1.27 (1.10–1.47)
Yang B, et al. 2017	USA	Prospective cohort study	303,620 participants	41.7%	18, 35, 50, and baseline (mean age 62.3 years, range 50.3–71.5 years)	4‐time points assessment (ages 18, 35, 50, and baseline), using retrospective self‐reported BMI with questionnaires	Hepatocellular carcinoma (HCC) and intrahepatic cholangiocarcinoma (ICC)	11.9 years	**Five Trajectories:** Stable normal (37%) Normal to overweight (46%) Normal to obese Class II (8%) Overweight to obese Class I (7%) Overweight to obese Class III (2%)	**HCC risk** ^a^: Stable normal: reference Normal to overweight: HR 1.28 (0.99–1.67) Normal to obese Class II: HR 1.84 (1.25–2.70) Overweight to obese Class I: HR 1.71 (1.17–2.50) Overweight to obese Class III: HR 1.83 (0.98–3.42) **ICC risk** ^a^: Stable normal: reference Normal to overweight: HR 1.14 (0.73–1.79) Normal to obese Class II: HR 1.54 (0.74–3.21) Overweight to obese Class I: HR 1.14 (0.49–2.63) Overweight to obese Class III: HR 1.76 (0.52–5.96)
Yang W, et al. 2022	USA	Prospective cohort study (Prostate, Lung, Colorectal and Ovarian Cancer Screening Trial)	138,922 (70,063 women and 68,859 men)	50.4%	BMI measured at ages 20, 50, and at study enrollment (ages 42–78)	3‐time points assessment Self‐reported BMI at ages 20 years, 50 years, and at enrollment	Liver cancer and biliary tract cancer (BTC)	15.9 years	**Four trajectories:** Stable normal: 28.1% Normal to overweight: 48.5% Normal to obese: 18.6% Overweight to obese: 4.8%	**Liver cancer risk** ^a^:: Stable normal: ref. Normal to overweight: 1.33 (0.89–2.05) Normal to obese: 2.50 (1.55–4.04) Overweight to obese: 1.99 (0.89–4.44) **Biliary tract cancer risk** ^a^: Stable normal: ref. Normal to overweight: 1.75 (1.09–2.79) Normal to obese: 1.83 (0.03–3.22) Overweight to obese: 4.26 (2.16–8.41)
Yang Y, et al. 2019	Australia	Prospective cohort study (Melbourne Collaborative Cohort Study)	29,881	NM	From age 18–21 years to late adulthood (participants were 40–70 years old at baseline)	4 points (age 18–21 years, 1990–1994, 1995–1998, and 2003–2007); measured weight at baseline and 2003–2007, self‐reported weight in 1995–1998, and recalled weight at age 18–21 years	Obesity‐related cancer mortality	NM	**Six trajectories:** Lower‐normal stable (19%) Higher‐normal stable (37.6%) Normal to overweight (27.2%) Chronic borderline obesity (3.1%) Normal to Class I obesity (9.7%) Overweight to Class II obesity (3.2%)	**Obesity‐related cancer mortality risk** ^a^:: Lower‐normal stable: Ref Higher‐normal stable: 1.18 (0.94, 1.48) Normal to overweight: 1.24 (0.97, 1.57) Chronic borderline obesity: HR 1.82, (1.24–2.68) Normal to Class I obesity: 1.49 (1.10, 2.01) Overweight to Class II obesity: 2.33 (1.56, 3.47)
Yang Y, et al. 2021	Australia	Cohort study (Melbourne Collaborative Cohort Study)	30,377 participants	55.7%	From age 18–21 to adulthood (age 40–85)	4 assessment points (age 18–21, baseline, follow‐up 1, follow‐up 2), Recalled for age 18–21 years, Measured at baseline and follow‐up 2, Self‐reported at follow‐up 1	Obesity‐related cancer including esophagus adenocarcinoma, postmenopausal Breast, liver, gallbladder, renal cell, colon and rectum, multiple myeloma, Meningioma, thyroid, gastric cardia, pancreas, ovary, and corpus Uteri	The follow‐up period is not explicitly stated, but it includes assessments from 1990–2007; therefore, the mean follow‐up time spans several years (approximately 10–17 years depending on the individual)	**Six trajectories:** Lower normal stable (19.4%) Higher normal stable (37.7%) Normal to overweight (26.8%) Chronic borderline obesity (3.2%) Normal to Class I obesity (9.7%) Overweight to Class II obesity (3.2%)	**Obesity‐related cancer risk** ^a^: Lower normal stable: reference Higher normal stable: HR 1.04 (0.92–1.18) Normal to overweight: HR 1.29 (1.13–1.47) Chronic borderline obesity: HR 1.12 (0.85–1.48) Normal to Class I obesity: HR 1.50 (1.28–1.75) Overweight to Class II obesity: HR 1.66 (1.32–2.08) **Colorectal cancer risk** ^a^: Lower normal stable: reference Higher normal stable: HR 0.86 (0.71–1.06) Normal to overweight: HR 1.10 (0.89–1.35) Chronic borderline obesity: HR 0.92 (0.60–1.41) Normal to Class I obesity: HR 1.11 (0.84–1.46) Overweight to Class II obesity: HR 1.47 (0.98–2.20) **Postmenopausal breast cancer risk** ^a^: Lower normal stable: reference Higher normal stable: HR 0.93 (0.76–1.14) Normal to overweight: HR 1.31 (1.05–1.62) Chronic borderline obesity: HR 1.18 (0.68–2.04) Normal to Class I Obesity: HR 1.55 (1.20–2.00) Overweight to Class II Obesity: HR 1.49 (1.02–2.18)
You D, et al. 2022	China	Population‐based cohort study	138,110 participants	49.52%	49–78 years	3 time points (ages 20, 50, and enrollment), Self‐reported height and weight	Nonsmall cell lung cancer (NSCLC)	NM	**Four trajectories:** Normal to normal (34.74%) Normal to overweight (46.70) Normal to obese (15.39%) Overweight to obese (3.16%)	**Nonsmall cell lung cancer risk** ^a^ **:** Normal to normal: Ref Normal to overweight: 0.77 (0.70–0.84) Normal to obese: 0.60 (0.53–0.69) Overweight to obese: 0.54 (0.40–0.74)
Zheng R, et al. 2018	USA	Cohort study (Prostate, Lung, Colorectal, and Ovarian Cancer Screening Trial ‐ PLCO)	139,229 participants	50.4%	Age 20, age 50, and baseline (mean age at baseline: 64.3 years)	Age 20 (self‐reported BMI) Age 50 (self‐reported BMI) Baseline (measured BMI at study enrollment)	Colorectal cancer (CRC)	13 years	**Four trajectories:** Normal BMI: 34.4% Normal BMI to Overweight: 46.3% Normal BMI to Obese: 16% Overweight to Obese: 16%	**All colorectal cancer (CRC) risk** ^a^ **:** Normal BMI: reference Normal BMI to Overweight: HR = 1.11 (0.99–1.23) Normal BMI to Obese: HR = 1.18 (1.03–1.35) Overweight to Obese: HR = 1.27 (0.99–1.64)
Aarestrup J, et al. 2017	Denmark	Cohort study (Copenhagen School Health Records Register)	155,505 girls	100%	Ages 6.25–14.0 years	Multiple measures (annual assessments, maximum 12 per participant), Measured BMI at school health exams, converted to z‐scores based on age‐specific references	Endometrial cancer (including estrogen‐dependent and non‐estrogen‐dependent cancers)	4.1 million person‐years	**Three trajectories:** Early childhood BMI growth (ages 6.25–7.99 years) Mid‐childhood BMI growth (ages 8.0–10.99 years) Late childhood BMI growth (ages 11.0–14.0 years)	**All Endometrial Cancers risk:** Reference for all: baseline average BMI growth (z‐score of 0) **Early Childhood BMI Growth (Ages** 6.25–7.99 years): HR 1.17 (1.05–1.30) per 0.1 z‐score increase Mid‐Childhood BMI Growth (Ages 8.0–10.99 years): HR 1.15 (0.94–1.40) Late Childhood BMI Growth (Ages 11.0–14.0 years): HR 1.10 (0.90–1.34) Combined Growth Estimate (6.25–14.0 years): HR 1.15 (1.07–1.24)
										**Estrogen‐Dependent Cancers risk:** Early Childhood BMI Growth (Ages 6.25–7.99 years): HR 1.19 (1.09–1.31) Mid‐Childhood BMI Growth (Ages 8.0–10.99 years): HR 1.12 (1.04–1.21) Late childhood BMI growth (Ages 11.0–14.0 years): HR 1.10 (0.90–1.34) Combined growth estimate (6.25–14.0 years): HR 1.12 (1.04–1.21) **Endometrioid adenocarcinomas:** Early childhood BMI growth (Ages 6.25–7.99 years): HR 1.19 (1.09–1.31) Midchildhood BMI growth (Ages 8.0–10.99 years): HR 1.16 (1.06–1.26) Late childhood BMI growth (Ages 11.0–14.0 years): HR 1.10 (0.90–1.34) Combined growth estimate (6.25–14.0 years): HR 1.16 (1.06–1.26) **Non–estrogen‐dependent cancers:** Early childhood BMI growth (Ages 6.25–7.99 years): HR 1.17 (1.05–1.30) Midchildhood BMI growth (Ages 8.0–10.99 years): HR 1.15 (0.94–1.40) Late childhood BMI growth (Ages 11.0–14.0 years): HR 1.46 (1.16–1.84) Combined growth estimate (6.25–14.0 years): HR 1.46 (1.16–1.84)
Deng Z, et al. 2023	USA	Prospective cohort study	138,614 participants	37.8% females	From age 20 years to baseline age (range: 51–78 years)	Assessment at ages 20, 50, and baseline Method: Self‐reported weight at these age points	Renal cell cancer (RCC)	11.5 years	**Four Trajectories:** Retained normal BMI throughout adulthood (34.8%) Normal BMI to overweight (46.7%) Normal BMI to obese (15.3%) Overweight to obese (3.2%)	**Renal cell cancer risk** ^a^ **:** Retained normal BMI throughout adulthood: Ref Normal BMI to overweight: HR 1.49 (1.19–1.87) Normal BMI to obese: HR 2.22 (1.70–2.90) Overweight to obese: HR 2.78 (1.81–4.27)

^a^ Full adjusted model.

^b^ Including cancers of the colorectum, esophagus (adenocarcinoma only), pancreas, kidney, breast (postmenopause), endometrium, ovaries, liver, and gallbladder.

^c^ Including cancers of the colorectum, esophagus (adenocarcinoma only), pancreas, kidney, prostate (advanced cancer only), liver, and gallbladder.

BMI was assessed at multiple time points across studies using three different measurement approaches. In 11 out of 29 (38%) studies, BMI was entirely recalled weight/BMI [8, 13, 14, 21, 26, 29, 30, 32, 34, 37, 42], while 52% combined measured BMI at certain time points with recalled weight/BMI at others [11, 12, 23, 25, 27, 28, 31, 33, 35, 36, 38, 39, 40, 41] and 10% of studies relied exclusively on measured BMI [9, 22, 24]. Except for one study (which used self‐report), all outcome measurements were based on record linkage.

Studies were conducted across various regions worldwide. North America had the largest representation, with 17 studies [9, 11, 12, 13, 23, 25, 26, 27, 28, 32, 33, 34, 36, 37, 38, 41, 42], 15 from the United States and two from Canada. Seven studies were conducted in Europe [8, 21, 24, 29, 31, 35, 43], with one study each from Norway, Italy, France, the UK, and Finland, and two studies from Denmark. There were three studies in Asia [14, 22, 30] (two from China, one from Taiwan) and two studies in Australia [39, 40].

### 3.2 Number and Nature of Trajectories and Outcomes

Studies both from childhood to adulthood and adulthood to adulthood predominantly identified increasing or stable BMI trajectories (Table 1). Only four [9, 21, 24, 35] reported decreasing BMI trajectories, shifts from obesity to lower BMI categories, including patterns such as obesity‐decrease, decreasing BMI, obesity‐to‐normal decreasing BMI, and average‐to‐low BMI. Additionally, one study [36] identified an N‐shaped trajectory with a partial decrease. Neither these decreasing trajectories nor the N‐shaped trajectory could be pooled with other BMI trajectories due to heterogeneity in trajectory shapes and insufficient data for meta‐analysis.

The trajectories varied in number and nature, with each included study identifying between three and six distinct trajectories, depending on the study's focus and population. Some studies identified three trajectories [28, 35, 43], typically involving categories normal weight, overweight, and obesity. Some others identified four [9, 12, 14, 22, 24, 25, 30, 32, 34, 36, 38, 41] or five [8, 11, 13, 21, 23, 26, 27, 29, 33, 37] trajectories, with more detailed classifications, such as normal to overweight, normal to obesity, stable normal, overweight to obesity, and stable obesity. A few studies identified six trajectories [31, 39, 40], incorporating additional variations such as lean stable, medium stable, and various stages of BMI increase or decrease throughout the life course.

The most frequently identified trajectories were normal to overweight or obesity and stable normal, each comprising approximately 40%–60% of participants in the studies. Among these, stable normal served as the reference trajectory in the majority of studies, making it the most common comparator for assessing cancer risk. Four studies employed lean stable as their reference category [26, 33, 36, 39]. Gender‐specific analyses were conducted in three studies [12, 22, 26].

The outcomes encompassed a broad range of specific individual cancer types, including breast cancer [21, 26, 31, 40], colorectal cancer [26, 30, 33, 40, 41], esophageal and gastric cancer [26, 32], liver and gallbladder cancer [37, 38], lung cancer [11, 14], renal cancer [26, 42], prostate cancer [9, 26, 27, 29, 34], endometrial cancer [8, 26, 43], ovarian cancer [26], and pancreatic cancer [12, 23, 25, 26]. Some studies also presented a composite outcome of obesity‐related cancers [26, 28, 36, 39, 40], composite or specific cancer mortality [13, 22, 27, 35, 39], gender‐specific cancers [12, 22, 26, 35, 36] and the composite outcome of all cancers [24, 26, 28].

The results of the quality assessment are presented in Tables S1 and S2. None of the studies included were classified as low or very low quality.

### 3.3 Results of Meta‐Analysis

The unadjusted results of the effect of different BMI trajectories from childhood/adulthood to adulthood on the composite and individual cancer risk outcomes are presented in Table S3, followed by the adjusted pooled results, which are shown in Table 2 and Figures 2 and S1–S10.

**TABLE 2:** Results of fixed/random effect meta‐analysis to estimate pooled adjusted ES (95% CI) for various cancer outcomes by trajectories.

Outcome	Trajectories	*N* ^a^	Heterogeneity *I* ^2^%^b^	Egger's publication bias^c^	Pooled adjusted ES (95% CI)
**Individual cancer outcome**					
Breast cancer	Stable normal	Reference			
	Normal to obese	4	83.9%	0.396	1.232 (0.968, 1.496)
	Normal to overweight	7	79.0%	0.945	1.047 (0.918, 1.176)
	Overweight to obese	4	45.0%	0.544	1.020 (0.836, 1.204)
Colorectal cancer	Stable normal	Reference			
	Normal to obese	4	0.0%	0.605	**1.170** (**1.096**, **1.245**)
	Normal to overweight	9	18.9%	0.739	**1.055** (**1.000**, **1.110**)
	Overweight to obese	6	0.0%	0.654	**1.306** (**1.175**, **1.437**)
Pancreatic cancer	Stable normal	Reference			
	Normal to obese	7	19.7%	0.954	**1.337** (**1.247**, **1.426**)
	Normal to overweight	11	0.0%	0.937	**1.136** (**1.079**, **1.194**)
	Overweight to obese	9	41.8%	0.629	**1.382** (**1.194**, **1.569**)
Liver cancer	Stable normal	Reference			
	Normal to obese	5	0.0%	0.772	**1.777** (**1.261**, **2.293**)
	Normal to overweight	5	0.0%	0.259	**1.335** (**1.060**, **1.609**)
	Overweight to obese	7	0.0%	0.540	**1.876** (**1.269, 2.483**)
Lung cancer	Stable normal	Reference			
	Normal to obese	3	77.4%	0.782	**0.676** (**0.531**, **0.822**)
	Normal to overweight	3	69.6%	0.854	**0.735** (**0.591**, **0.879**)
	Overweight to obese	3	82.2%	0.757	**0.714** (**0.415**, **1.012**)
Prostate cancer	Stable normal	Reference			
	Normal to obese	8	48.4%	0.389	0.981 (0.893, 1.069)
	Normal to overweight	11	0.0%	0.211	0.966 (0.923, 1.009)
	Overweight to obese	9	38.3%	0.747	**0.680** (**0.599**, **0.760**)
Kidney cancer	Stable normal	Reference			
	Normal to obese	2	0.0%	—	**2.122** (**1.619**, **2.624**)
	Normal to overweight	6	5.9%	0.361	1.175 (0.982, 1.369)
	Overweight to obese	3	76.6%	0.099	1.771 (0.673, 2.870)
Endometrial Cancer	Stable Normal	Reference			
	Normal to obese	0	—	—	—
	Normal to overweight	5	60.1%	0.136	1.165 (0.898, 1.432)
	Overweight to obese	2	0.0%	—	**2.066** (**1.583**, **2.549**)
**Composite cancer outcomes**					
Women‐related cancer	Stable normal	Reference			
	Normal to obese	5	80.2%	0.506	1.167 (0.948, 1.387)
	Normal to overweight	14	69.2%	0.764	1.042 (0.942, 1.141)
	Overweight to obese	7	76.0%	0.130	1.204 (0.935, 1.472)
Obesity‐related cancer	Stable normal	Reference			
	Normal to obese	24 + 7^d^	65.0%	0.014	**1.359** (**1.253**, **1.464**) **1.281** (**1.162**, **1.399** ^d^)
	Normal to overweight	44 + 11^d^	10.0%	0.016	**1.101** (**1.072**, **1.129**) **1.088** (**1.060**, **1.116** ^d^)
	Overweight to obese	33	29.5%	0.067	**1.307** (**1.236**, **1.378**)
Gastrointestinal cancers	Stable normal	Reference			
	Normal to obese	3	36.4%	0.875	**2.061** (**1.476**, **2.645**)
	Normal to overweight	7	0.0%	0.749	**1.401** (**1.198**, **1.604**)
	Overweight to obese	4	0.0%	0.886	**3.126** (**1.900**, **4.353**)
Overall cancers	Stable normal	Reference			
	Normal to obese	42 + 14^d^	88.6%	0.011	**1.228** (**1.121**, **1.335**) 1.081 (0.976, 1.186^d^)
	Normal to overweight	79 + 22^d^	67.3%	0.012	**1.062** (**1.024**, **1.100**) 1.015 (0.977, 1.053^d^)
	Overweight to obese	56 + 19^v^	80.4%	0.021	**1.168** (**1.059**, **1.277**) 1.063 (0.950, 1.176^d^)

*Note:* —, Insufficient data; bold values indicate significant results.

^a^ Number of records to get pooled.

^b^ *I* ^2^, chi^2^ test.

^c^ Harbord publication bias test for odds ratio.

^d^ Random effect Mantel–Haenszel method.

**FIGURE 2:**
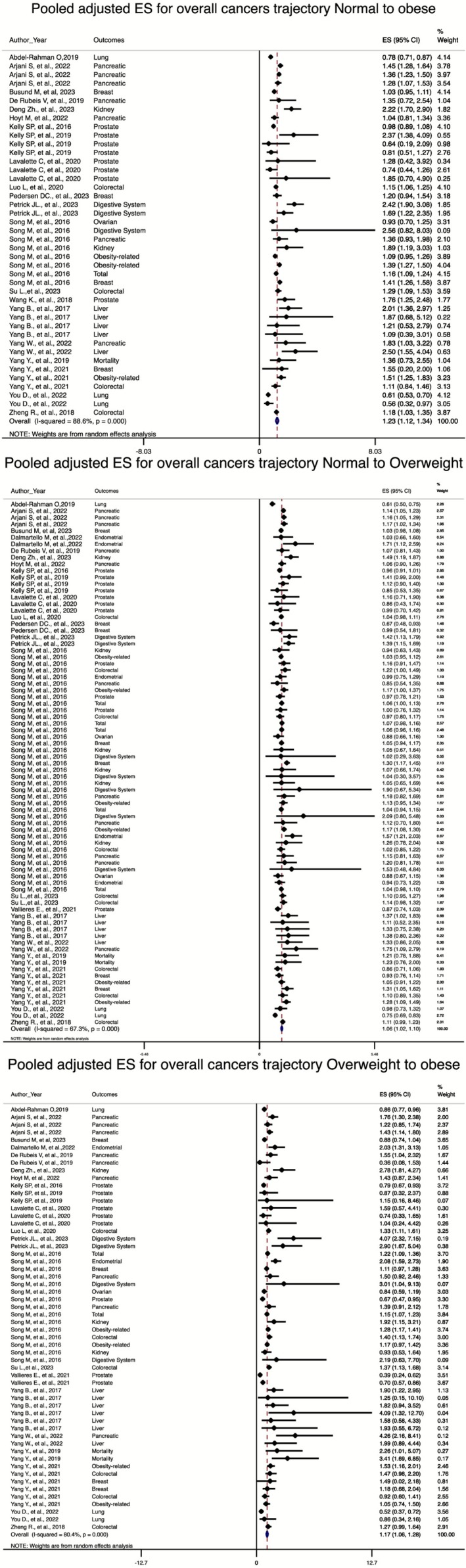
Forest plots for the pooled effect size (ES) of composite outcome of overall cancers for different BMI trajectories compared to stable normal weight trajectory: (A) ES of composite outcome of overall cancers for normal to obesity trajectory; (B) ES of composite outcome of overall cancers for normal to overweight trajectory; (C) ES of composite outcome of overall cancers for overweight to obesity trajectory.

#### 3.3.1 Individual Cancer Outcomes

Regarding colorectal cancer, individuals following a normal to obesity trajectory exhibited a significantly increased risk compared to those maintaining a normal BMI (pooled ES adjusted for confounders: 1.170; 95% CI: 1.096–1.245; *I* ^2^ = 0.0%). The normal‐to‐overweight trajectory also showed a slightly elevated but significant risk (pooled ES adjusted for confounders: 1.055; 95% CI: 1.000–1.110; *I* ^2^ = 18.9%). The highest risk was seen in those with an overweight to obesity trajectory (pooled ES adjusted for confounders: 1.306; 95% CI: 1.175–1.437; *I* ^2^ = 0.0%), (Table 2 and Figure S4).

For pancreatic cancer, both the normal to obesity and normal to overweight trajectories were significantly associated with increased cancer risk. Specifically, individuals following a normal to obesity trajectory had a pooled ES = 1.337 (95% CI: 1.247–1.426; *I* ^2^ = 19.7%), while the normal‐to‐overweight trajectory had an ES = 1.136 (95% CI: 1.079–1.194; *I* ^2^ = 0.0%). The overweight to obesity trajectory further elevated the risk, with a pooled ES = 1.382 (95% CI: 1.194–1.569; *I* ^2^ = 41.8%), (Table 2 and Figure S5).

The risk of liver cancer was significantly increased for individuals following a normal to obesity trajectory, with a pooled ES = 1.777 (95% CI: 1.261–2.293; *I* ^2^ = 0.0%). Similarly, those with a normal‐to‐overweight trajectory exhibited a moderate increase in risk (pooled ES adjusted for confounders: 1.335; 95% CI: 1.060–1.609; *I* ^2^ = 0.0%). The overweight to obesity trajectory had a consistent association, with ES = 1.876 (95% CI: 1.269–2.483; *I* ^2^ = 0.0%) (Table 2 and Figure S6).

Regarding kidney cancer, individuals following a normal to obesity trajectory showed a significant increase in risk (pooled ES adjusted for confounders: 2.122; 95% CI: 1.619–2.6249, *I* ^2^ = 0%). A nonsignificant association with an increased risk of kidney cancer was observed for the normal‐to‐overweight and overweight to obesity trajectories (Table 2 and Figure S7).

For endometrial cancer, the overweight to obesity trajectory showed a significant association with increased risk (pooled ES adjusted for confounders: 2.066; 95% CI: 1.583–2.549; *I* ^2^ = 0.0%). No data were available for the normal to obesity trajectory, and no significant association was found for the normal‐to‐overweight trajectory (Table 2 and Figure S8).

Regarding prostate cancer, no significant association was observed for individuals following normal to obesity and normal‐to‐overweight trajectories. The overweight to obesity trajectory was significantly associated with a reduced risk of prostate cancer, with a pooled adjusted ES: 0.680 (95% CI: 0.599–0.760; *I* ^2^ = 38.3%), (Table 2 and Figure S9).

For lung cancer, all BMI trajectories (normal to obesity, normal‐to‐overweight, and overweight to obesity) were associated with a decreased risk of lung cancer, compared to the stable normal trajectory. The normal to obesity trajectory showed a significant protective effect (ES = 0.676, 95% CI: 0.531–0.822, *I* ^2^ = 77.4%). The normal‐to‐overweight trajectory also demonstrated a slight protective effect (ES = 0.735, 95% CI: 0.591–0.879, *I* ^2^ = 69.6%), while the overweight to obesity trajectory showed a nonsignificant protective effect (ES = 0.714, 95% CI: 0.415–1.012, *I* ^2^ = 82.2%). (Table 2 and Figure S10).

#### 3.3.2 Composite Cancer Outcomes

Regarding the composite outcome of overall cancers, individuals with a trajectory from normal weight at study entry progressing to obesity had an increased risk of overall cancers compared to those who maintained a normal weight (pooled ES adjusted for confounders: 1.228; 95% CI: 1.121–1.335; *I* ^2^ = 88.6%). After adjusting for potential publication bias, this association was no longer significant (ES = 1.081; 95% CI: 0.976–1.186, *I* ^2^ = 88.6%). Similarly, those following a normal‐to‐overweight trajectory experienced a modestly increased risk of overall cancers (pooled ES adjusted for confounders: 1.062; 95% CI: 1.024–1.100; *I* ^2^ = 67.3%). After accounting for publication bias, the adjusted ES was 1.015 (95% CI: 0.977–1.053, *I* ^2^ = 67.3%). Lastly, overweight to obesity trajectories were significantly associated with cancer risk (pooled ES adjusted for confounders: 1.168; 95% CI: 1.059–1.277; *I* ^2^ = 80.4%). After adjusting for potential publication bias, this association was no longer significant (ES = 1.063, 95% CI: 0.950–1.176, *I* ^2^ = 80.4%), (Table 2 and Figures 2A–C and S11).

Regarding obesity‐related cancers, individuals with a trajectory from normal weight to obesity showed a significantly increased risk compared to those who maintained a normal weight (pooled ES adjusted for confounders: 1.359; 95% CI: 1.253–1.464; *I* ^2^ = 65.0%). After adjusting for publication bias, the effect size was slightly reduced but remained significant (ES = 1.281; 95% CI: 1.162–1.399; *I* ^2^ = 65.0%). Those following a normal‐to‐overweight trajectory also exhibited a significant increase in risk (pooled ES adjusted for confounders: 1.101; 95% CI: 1.072–1.129; *I* ^2^ = 10.0%), with a publication bias‐adjusted ES of 1.088 (95% CI: 1.060–1.116). Lastly, overweight to obesity trajectories were associated with a significantly increased risk of obesity‐related cancers (pooled ES adjusted for confounders: 1.307; 95% CI: 1.236–1.378; *I* ^2^ = 29.5%). Although the publication bias‐adjusted effect size showed a minor reduction, the significance remained (ES = 1.307; 95% CI: 1.236–1.378; *I* ^2^ = 29.5%) (Table 2 and Figures S1A–C and S11).

Regarding composite outcome of gastrointestinal cancers, individuals with a normal to obesity trajectory showed a notably increased risk compared to those who maintained a normal weight (pooled ES adjusted for confounders: 2.061; 95% CI: 1.476–2.645; *I* ^2^ = 36.4%, *p* = 0.875). Those with a normal‐to‐overweight trajectory also exhibited a significantly elevated risk of gastrointestinal cancers (pooled ES adjusted for confounders: 1.401; 95% CI: 1.198–1.604; *I* ^2^ = 0.0%, *p* = 0.749). The highest risk was observed in individuals following an overweight to obesity trajectory, with a pooled ES adjusted for confounders of 3.126 (95% CI: 1.900–4.353; *I* ^2^ = 0%), (Table 2 and Figure S2).

Regarding women's cancers, compared to the stable normal trajectory, all BMI trajectories (normal to obesity, normal‐to‐overweight, and overweight to obesity) were associated with an increased risk compared to the stable normal trajectory, although the increases were not statistically significant (Table 2 and Figure S3).

#### 3.3.3 Subgroup and Sensitivity Analyses

To assess whether the timing of initial BMI measurements influenced cancer risk estimates, we conducted a subgroup analysis by stratifying studies according to the age at the first BMI measurement (childhood vs. adulthood). Four studies initiated BMI assessments in childhood [23, 26, 31, 37]. Using meta‐regression, we compared the pooled adjusted ES for cancer outcomes across BMI trajectories between studies starting in childhood and those starting in adulthood, with the stable normal trajectory as the reference group. No statistically significant differences were observed between the two groups (data not shown). Additional subgroup analyses were performed based on follow‐up duration (< 15 years vs. ≥ 15 years), BMI assessment method (measured vs. recalled), and geographic region. This analysis indicated that the heterogeneity across studies was largely explained by differences in follow‐up duration, BMI assessment methods, and geographic regions. Studies using measured BMI, longer follow‐up periods, and conducted in European populations tended to yield more homogeneous and precise estimates. Despite heterogeneity in some subgroups (such as women's cancers), the direction of association remained consistent, which supports the robustness of the link between unfavorable BMI trajectories (particularly normal to obesity and overweight to obesity) and increased cancer risk (Table S4 and Figure S11). Due to lack of data, subgroup analysis was not performed based on ethnicity or for site‐specific cancers.

Sensitivity analyses, conducted sequentially excluding individual studies, showed no material change in the overall pooled estimates. This suggests that no single study disproportionately influenced the observed associations, supporting the stability and robustness of the main findings (Supplementary Figure S12).

## 4 Discussion

The results of this systematic review and meta‐analysis indicate that BMI trajectories from childhood/adulthood to later adulthood are significantly associated with risk of cancer, with varying effects depending on the trajectory and cancer type. Individuals who progressed from normal weight to obesity or overweight to obesity exhibited an increased risk of obesity‐related cancers and gastrointestinal cancers compared to those maintaining normal weight. Similar patterns were observed for specific cancers, including colorectal, pancreatic, liver, and kidney cancers. However, for certain outcomes, such as breast cancer and women's cancers, the associations were not statistically significant despite a clear trend toward increased risk. Additionally, the findings for prostate cancer were mixed. Conversely, for lung cancer, the pooled results of our meta‐analysis showed that all increasing BMI trajectories were associated with a decreased risk.

Obesity is a well‐established risk factor for various cancers [44]. However, the underlying mechanisms resulting in carcinogenesis are complex and not yet fully understood. Altered fatty acid secretion and metabolism, extracellular matrix remodeling, the secretion of anabolic and sex hormones, immune dysregulation, chronic inflammation, and changes in the gut microbiome have been linked to carcinogenesis, the development of metastases, and the progression of cancer in obesity [44, 45, 46, 47]. It is likely that different mechanisms lead to the development of different cancers. Emerging evidence has demonstrated that chronic low‐grade inflammation associated with prolonged obesity promotes tumor initiation and progression [45], while hormonal alterations, including elevated insulin, IGF‐1, and estrogen levels, create an environment conducive to carcinogenesis [47, 48]. Visceral adiposity further activates oncogenic pathways such as mTOR, enhancing tumor cell proliferation [49]. Additionally, obesity‐induced epigenetic modifications may accumulate over time, leading to dysregulated gene expression that heightens cancer susceptibility [50]. Further, it is shown that childhood overweight or obesity and long‐term adulthood obesity are associated with increased risk of cancer [51, 52]. It should be noted that several factors, including socioeconomic or sociodemographic and behavioral factors, may also be related to high BMI or to cancer risks and outcomes [53, 54]. Some studies included in this review adjusted for various variables, including sex, age, physical activity, alcohol, smoking, diet, diabetes mellitus, and alcohol consumption.

Emerging evidence highlights cancer as a tumor microenvironment (TME) disease [55], driven by obesity‐induced metabolic reprogramming that fosters TME heterogeneity, including immunosuppressive macrophages and fibroblasts that promote tumor progression and therapy resistance. Chronic diseases like type 2 diabetes exacerbate this through obesity‐driven dysbiosis and inflammation, elevating cytokines such as IL‐6 and TNF‐α that link systemic metabolic dysfunction to pro‐tumorigenic immune shifts [56]. The mTOR pathway, dysregulated in obesity, drives oncogenic proliferation, with mTOR inhibitors showing promise in disrupting cancer stem cells and enhancing immunotherapy. Obesity also amplifies glycolysis, increasing lactate efflux to acidify the TME and promote metastasis via HIF‐1α‐mediated mechanisms [57, 58, 59, 60, 61].

In this meta‐analysis, we found that increasing BMI trajectories from normal weight to overweight or obesity were significantly associated with elevated risks of certain cancers, such as colorectal, pancreatic, liver, and kidney cancers, compared to the stable normal weight trajectory. Focusing on BMI trajectories over the life course, which provides an understanding of how weight gain patterns influence cancer risk, is a strength of this study compared to traditional analyses based on BMI at a single time point. Prior evidence has consistently linked higher BMI in adulthood to increased cancer risk, but such findings do not capture the long‐term impact of weight gain trajectories or differentiate between those who have been overweight or obese since childhood versus those who transitioned into these categories later in life. Our findings suggest that individuals who transition from normal weight to obesity are at significantly higher risk for cancer. Unlike cross‐sectional BMI measures, trajectory‐based analyses can account for cumulative exposure to excess adiposity and better reflect the metabolic and inflammatory consequences of prolonged weight gain. This may explain why certain cancers show particularly strong associations with BMI trajectories, as prolonged exposure to obesity‐related mechanisms such as chronic low‐grade inflammation, insulin resistance, and hormonal dysregulation may be necessary to drive carcinogenesis. Additionally, by analyzing BMI trajectories, we can differentiate between individuals who have remained at a stable weight and those who experienced substantial weight gain.

However, for certain outcomes, such as breast cancer and women's cancers, the associations were not statistically significant despite a clear trend toward increased risk. Additionally, the findings for prostate cancer were mixed. In line with our findings, Shi et al. (2024) [5], in a meta‐analysis of 66 observational studies, evaluated the association between BMI and weight change on the risk of developing cancer. They found that compared to underweight or normal weight, overweight or obesity was associated with an increased risk of endometrial cancer, kidney cancer, and liver cancer but a decreased risk of prostate cancer and lung cancer. Additionally, weight gain of more than 5 kg increased overall cancer risk, particularly breast cancer risk, but had no significant effect on colon or reproductive cancer. Smaller gains showed no significant impact. Conversely, weight loss of more than 5 kg lowered overall cancer risk, specifically reducing the risk of breast and reproductive cancers, while having no significant effect on colon cancer. One possible explanation is that the nature of cancer development involves complex interactions between genetic, environmental, and lifestyle factors; also, the relationship between obesity and certain cancers, such as prostate or breast cancers, may be more influenced by hormonal factors, including estrogen, androgens, and insulin resistance, rather than BMI alone.

Particularly for breast cancer, adiposity influences circulating estrogen levels through increased aromatization in adipose tissue, which is especially relevant in postmenopausal women [62]. In this respect, adipose tissue acts as the major reservoir for estrogen biosynthesis after menopause. Elevated estrogen levels in serum along with enhanced peripheral site production of estrogen have been identified as major contributors to the development of breast cancer in overweight postmenopausal women [63]. In premenopausal women, higher BMI might influence aggressive tumor characteristics and has divergent impacts on the risk of different breast cancer subtypes [64].

For prostate cancer, our meta‐analysis found no significant association for normal‐to‐obesity or normal‐to‐overweight trajectories, while the overweight‐to‐obesity trajectory was associated with a reduced risk. This inverse association contrasts with biological hypotheses suggesting that obesity may increase prostate cancer risk through mechanisms such as hormonal alterations (testosterone, insulin, IGF‐1), chronic low‐grade inflammation, oxidative stress, and dysregulated adipokine signaling, which could promote epithelial‐to‐mesenchymal transition and a more aggressive phenotype [65, 66]. Moreover, one plausible explanation for this discrepancy is detection bias, as men with obesity may exhibit lower PSA levels [67, 68], which may lead to fewer biopsy referrals and underdiagnosis, artificially attenuating observed associations. Alternatively, the relationship between adiposity and prostate cancer may be more complex, potentially influenced by competing risks, metabolic adaptations, or differences in tumor biology. Age further modifies these relationships, as both hormonal milieu and cancer biology may differ across the life course. Taken together, these factors such as age, screening bias, and hormonal factors may affect the inconsistent associations observed between BMI trajectories and hormone‐related cancers in our review.

The limited statistical power due to insufficient data in some subgroup analyses may have contributed to inconclusive results. Another important consideration is that the use of BMI as a measure of obesity presents significant limitations in assessing cancer risk. While BMI is widely used in epidemiological research due to its simplicity, reproducibility, and strong correlation with body fat, it does not distinguish between fat mass and lean mass and therefore may misclassify individuals with high muscularity as living with overweight or obesity [69, 70]. Furthermore, BMI cannot capture body fat distribution, particularly visceral adiposity, which is strongly linked to metabolic dysfunction and cancer risk [71, 72, 73]. Emerging evidence suggests that visceral adiposity and central obesity are more strongly associated with cancer risk than BMI‐defined obesity [71, 72, 74]. In addition, BMI thresholds have variable applicability across ethnic populations. For example, Asian populations experience higher cardiometabolic and cancer risks at lower BMI values than European populations, prompting the World Health Organization (WHO) to recommend lower BMI cut‐offs for overweight and obesity in Asian groups [75, 76, 77]. Furthermore, gender differences and aging can influence BMI and interact with obesity‐related health risks. These BMI limitations should be considered when interpreting our findings, as they may partly contribute to between‐study heterogeneity and limit the generalizability of BMI‐based trajectories across populations.

The pooled results of our meta‐analysis showed that all increasing BMI trajectories were associated with a decreased risk of lung cancer, compared to stable normal BMI. However, this apparent association should be interpreted with caution, as only a limited number of original studies examined BMI trajectories for lung cancer, potentially leading to inconclusive results. One possible explanation for this inverse association is the well‐established link between smoking and lower body weight, as nicotine suppresses appetite and increases metabolic rate [78]. Since smoking is by far the most important risk factor for lung cancer [79], individuals with lower BMI may be more likely to have a history of smoking, introducing confounding into the observed relationship. It should be noted that this association may be influenced by reverse causality, depending on the timing of final BMI measurement relative to cancer diagnosis. Lung cancer's highly catabolic nature can lead to significant weight loss during the extended lag between early symptoms and diagnosis [80, 81], potentially lowering BMI before measurement, further complicating this association. Additionally, genetic analysis revealed that higher BMI interacts with specific SNPs involved in cell growth, differentiation, and inflammation, potentially contributing to a lower risk of lung cancer. Key genes such as *BRAF, CACNA1A, CLASP2*, and *SLC16A7* regulate cellular signaling, calcium homeostasis, microtubule stability, and metabolic processes, mechanisms that may reduce lung cancer development. Additionally, higher nutrient intake in individuals with elevated BMI could further reduce risk by modulating inflammatory and metabolic pathways [14, 82, 83].

The heterogeneity in effect sizes across cancer types likely reflects differences in the pathological mechanisms linking adiposity to carcinogenesis. In obesity‐related cancers, excess adiposity contributes through multiple pathways, including chronic low‐grade inflammation, hyperinsulinemia, insulin resistance, and increased bioavailable sex hormones [46, 84, 85, 86]. As such, positive energy balance induces a variety of systemic changes including altered levels of insulin, insulin‐like growth factor‐1, leptin, adiponectin, steroid hormones, and cytokines [46]. Each of these factors alters the nutritional milieu and has the potential to create an environment that favors tumor initiation and progression [46]. Additionally, hormone‐sensitive cancers, such as endometrial and postmenopausal breast cancer, are further influenced by obesity‐related hyperestrogenism, driven by increased aromatization of androgens in adipose tissue [87]. Aromatase is the rate‐limiting enzyme in estrogen biosynthesis, and its expression in adipose stromal cells is hypothesized to drive the growth of breast tumors and confer resistance to endocrine therapy in postmenopausal women with obesity. The molecular regulation of aromatase has been characterized in response to many obesity‐related molecules, including inflammatory mediators and adipokines [88]. Additionally, site‐specific factors, such as visceral fat accumulation and the tumor microenvironment, likely modulate the magnitude of associations across cancer sites. It is worth considering that an increasing BMI trajectory may reflect prolonged exposure to lifestyle factors such as low physical activity levels and suboptimal dietary patterns. Over time, excessive caloric intake, poor nutritional quality, and sedentary behavior may contribute to BMI gain, potentially influencing cancer risk independently of BMI change itself [89, 90, 91]. Although most included studies adjusted for common factors such as age, sex, and smoking, data on dietary patterns, physical activity, and socioeconomic status or other lifestyle behaviors were not consistently available across studies. Inadequate adjustment of these important variables [91, 92, 93, 94] may have led to over‐ or under‐estimation of associations, limiting our ability to assess these effects. Future research should aim to incorporate detailed lifestyle factors, alongside BMI trajectories, to better disentangle their independent and combined contributions to cancer risk.

In the current meta‐analysis, we identified publication bias in some outcomes. This type of bias is common in meta‐analyses, as studies with significant or positive results are more likely to be published. To address this, we performed a trim‐and‐fill correction to adjust for the potential impact of publication bias.

Additionally, we observed some degree of heterogeneity across the outcomes. This variability may be explained by several factors. The studies included in the analysis differed in their follow‐up periods, study populations (including variations in age groups, geographic locations, and ethnic backgrounds), as well as the methods used to ascertain BMI (including such as direct measurement, self‐report, or a combination of both). Furthermore, variations in BMI trajectories definitions and classification methods across different populations and studies could also be a potential source of heterogeneity. Although most included studies applied latent class growth or mixture modeling approaches, the number, shape, and labeling of trajectories varied considerably, with some studies identifying as few as three categories, while others reported up to six distinct patterns. These methodological inconsistencies likely contributed to between‐study variation in effect estimates and could complicate direct comparisons of cancer risks across trajectory groups. In addition, our subgroup analyses helped clarify some of the underlying sources of heterogeneity. Studies with longer follow‐up durations (≥ 15 years) generally demonstrated lower heterogeneity and more stable estimates, which suggests that extended observation periods may provide a more accurate reflection of the long‐term influence of BMI changes on cancer development. Similarly, analyses restricted to studies using measured BMI values yielded more consistent results with narrower confidence intervals, whereas those relying on recalled or self‐reported BMI showed greater variability, likely due to recall bias or misclassification of weight status. Geographic differences also appeared to play a role: European cohorts tended to produce more homogeneous results than studies conducted in American populations, potentially reflecting variations in lifestyle, other cancer determinants, healthcare systems, or cancer surveillance practices. Despite these differences, the direction of associations remained consistent across all subgroup analyses, supporting the robustness of the overall findings. Sensitivity analyses, in which studies were systematically excluded one by one, further confirmed the stability and reliability of the pooled estimates.

Our findings demonstrate that distinct BMI trajectories are associated with differential cancer risks, which emphasizes the potential utility of moving beyond single time‐point BMI in both clinical and public health strategies. While individuals with obesity at any given time already represent a high‐risk population [5, 95], our results suggest that those with persistent or progressively increasing obesity trajectories constitute a particularly vulnerable subgroup, potentially reflecting longer cumulative exposure to metabolic and inflammatory risk factors. Recognizing these longitudinal patterns and contextual influences could help identify individuals at highest risk, who may benefit from targeted, earlier intervention through structured weight‐management programs, lifestyle modification, and where clinically indicated, medical or surgical approaches. Preventive measures should consider life‐course factors, including transitions between life phases and major life events.

Along with risk‐based approaches, population‐based public health strategies should also be considered [96]. Further, preventive interventions should target different eco‐social levels across the life course that account for sensitive periods, pathways, and accumulated risk factors [97]. Although our study does not assess the impact of specific weight‐management interventions, the results reinforce broader public‐health strategies that promote healthy weight maintenance across the life course. Early prevention, particularly during young adulthood, remains crucial, alongside supportive environments that facilitate sustained healthy behaviors. Incorporating demographic and cultural factors may further enhance the effectiveness and equity of these prevention initiatives.

These results may also have implications for cancer prevention guidelines. Current guidelines [98, 99, 100, 101] mainly focus on maintaining a BMI within the normal range (18.5–24.9 kg/m^2^) but do not account for longitudinal BMI trajectories. Incorporating longitudinal weight/BMI history into risk assessment or BMI trajectory‐based risk stratification into clinical practice, rather than relying only on current BMI, may improve identification of individuals at elevated cancer risk. Cancer prevention policies could therefore be revised to recommend trajectory‐based risk stratification, with individuals on high‐risk patterns prioritized for intensive weight‐management support. An important consideration that warrants further investigation is the timing of BMI changes across the life course. The age at which an individual transitions from normal weight to overweight or obesity may have important implications for cancer risk, as earlier onset and longer duration of obesity could lead to greater cumulative metabolic and inflammatory burden. Although the data included in this meta‐analysis did not allow for age‐specific analyses, future longitudinal studies should examine BMI trajectories within defined age intervals to identify critical periods of vulnerability.

The primary strength of this systematic review and meta‐analysis was providing, for the first time, insights into life‐course BMI trajectories and their impact on cancer risk, offering deeper understanding compared to single‐time‐point BMI categories. Unlike static BMI assessments, which capture risk at specific moments, our trajectory‐based analysis reveals longitudinal BMI trajectories and their cumulative effects on cancer risk. The high quality of the original studies allowed us to present reliable findings. This study has several limitations that should be noted. In interpreting our findings, it is important to consider potential methodological limitations that may partly explain the observed associations. First, some subgroup analyses included a relatively small number of observations, which may have limited statistical power and led to unstable effect estimates. Additionally, selective reporting bias cannot be ruled out, as studies with null or nonsignificant findings may be underrepresented in the published literature, potentially inflating observed associations. To address this, we conducted statistical tests to evaluate publication bias, which indicated that most included studies were unaffected. For outcomes where publication bias was detected, we applied trim‐and‐fill adjustments; however, residual effects may still remain. Furthermore, systematic biases in exposure and outcome assessment may have influenced the study results. Several included studies relied on recalled or self‐reported BMI, some spanning long periods of up to 50 years or more, which introduces the potential for recall bias. Additionally, heterogeneity in trajectory modeling approaches may have contributed to inconsistent findings across studies. Similarly, variations in follow‐up duration and adjustments for confounding variables may have affected comparability across studies. In addition, due to a lack of data in most studies, we were unable to estimate a crude pooled result and subsequently adjust for confounders. However, most of the included studies adjusted for relevant covariates, allowing us to pool and calculate adjusted findings. It should be noted, however, that potential confounding could not be fully eliminated, as all the studies included were observational and did not adjust for all potential confounders. Another limitation of this study was the lack of data on “high stable” BMI trajectories, where individuals maintain a consistently elevated BMI over time, or on any trajectories that represented decreasing BMI over time, which would both potentially offer valuable insights into the differential impact of prolonged exposure to high BMI on cancer risk. Another limitation of this review is that none of the included studies reported the time interval between the last BMI measurement and cancer diagnosis. This gap prevents assessment of potential reverse effects of preclinical disease and limits interpretation of the temporal relationship between BMI trajectories and cancer risk. Finally, due to insufficient data in the original studies, we were unable to conduct a subgroup analysis based on ethnicity, which may be a potential source of heterogeneity.

## 5 Conclusion

The findings of this systematic review and meta‐analysis indicate that the transition from normal weight to overweight or obesity throughout life is associated with an increased risk of cancer, with varying effects depending on the trajectory and cancer type. This epidemiological knowledge is valuable in preventive public health efforts concerning both population‐based and risk‐based strategies. Further epidemiological follow‐up studies with larger sample sizes and analyses stratified by gender, specific age intervals, and specific types of cancer, specifically women's cancers, are warranted. Additionally, studies using alternative obesity indices and more precise measures of adiposity are needed to better understand these associations.

## Author Contributions

**S.B.‐G.**, **T.H.**, **R.B.‐Y.:** study concept and design. **R.B.‐Y., S.B.‐G., T.H.:** data analysis. **S.B.‐G., N.C.V.M., R.B.‐Y.:** drafting of the manuscript. **S.B.‐G., M.N., N.C.V.M., T.H., E.C.A., M.S.H.:** critical revision of the manuscript. All authors have read and approved the final manuscript. Open access funding provided by Nord University.

## Conflicts of Interest

The authors declare no conflicts of interest.

## Supporting information




**Table S1:** Quality assessment of the included studies using the Newcastle–Ottawa Quality Assessment Scale for cohort studies.
**Table S2:** Quality assessment of the included studies using the Newcastle–Ottawa Quality Assessment Scale for case–control studies.
**Table S3:** Results of Fixed/Random effect meta‐analysis to estimate pooled unadjusted ES (95% CI) for various cancer outcomes by trajectories.
**Table S4:** Results of Random effect sub‐group analysis to estimate pooled unadjusted ES (95% CI) for various cancer outcomes by trajectories based on follow‐up duration (< 15 years vs. ≥ 15 years), BMI assessment method (measured vs. recalled), and geographic region.
**Figure S1:** Forest plots for the pooled effect size (ES) of composite outcome of obesity related cancers for different BMI trajectories compared to stable normal weight trajectory: (A) ES of composite outcome of obesity related for normal to obesity trajectory; (B) ES of composite outcome of obesity related for normal to overweight trajectory (C) ES of composite outcome of obesity related for overweight to obesity trajectory.
**Figure S2:** Forest plots for the pooled effect size (ES) of composite outcome of gastrointestinal cancers for different BMI trajectories compared to stable normal weight trajectory.
**Figure S3:** Forest plots for the pooled effect size (ES) of composite outcome women related cancers for different BMI trajectories compared to stable normal weight trajectory.
**Figure S4:** Forest plots for the pooled effect size (ES) of colorectal cancer for different BMI trajectories compared to stable normal weight trajectory.
**Figure S5:** Forest plots for the pooled effect size (ES) of pancreatic cancer for different BMI trajectories compared to stable normal weight trajectory.
S6. Forest plots for the pooled effect size (ES) of liver cancer for different BMI trajectories compared to stable normal weight trajectory.
**Figure S7:** Forest plots for the pooled effect size (ES) of kidney cancer for different BMI trajectories compared to stable normal weight trajectory.
**Figure S8:** Forest plots for the pooled effect size (ES) of endometrial cancer for different BMI trajectories compared to stable normal weight trajectory.
**Figure S9:** Forest plots for the pooled effect size (ES) of prostate cancer for different BMI trajectories compared to stable normal weight trajectory.
**Figure S10:** Forest plots for the pooled effect size (ES) of lung cancer for different BMI trajectories compared to stable normal weight trajectory.
**Figure S11:** Funnel plot assessing publication bias in the association between BMI trajectories and obesity related cancer and overall cancers risks.
**Figure S12:** Forest plots for the pooled effect size (ES) of subgroup analysis for different BMI trajectories.
**Figure S13:** Sensitivity analysis.

## Data Availability

All relevant data presented in this manuscript are available upon request.

## References

[1] F. Bray , M. Laversanne , E. Weiderpass , and I. Soerjomataram , “The Ever‐Increasing Importance of Cancer as a Leading Cause of Premature Death Worldwide,” Cancer 127 (2021): 3029–3030. 34086348 10.1002/cncr.33587

[2] R. Zheng , S. Wang , S. Zhang , et al., “Global, Regional, and National Lifetime Probabilities of Developing Cancer in 2020,” Sci Bull (Beijing) 68 (2023): 2620–2628. 37821267 10.1016/j.scib.2023.09.041 PMC10640926

[3] D. C. Whiteman and L. F. Wilson , “The Fractions of Cancer Attributable to Modifiable Factors: A Global Review,” Cancer Epidemiology 44 (2016): 203–221. 27460784 10.1016/j.canep.2016.06.013

[4] C. Koliaki , M. Dalamaga , and S. Liatis , “Update on the Obesity Epidemic: After the Sudden Rise, Is the Upward Trajectory Beginning to Flatten?,” Current Obesity Reports 12 (2023): 514–527. 37779155 10.1007/s13679-023-00527-y PMC10748771

[5] X. Shi , G. Deng , H. Wen , et al., “Role of Body Mass Index and Weight Change in the Risk of Cancer: A Systematic Review and Meta‐Analysis of 66 Cohort Studies,” Journal of Global Health 14 (2024): 04067. 38547495 10.7189/jogh.14.04067 PMC10978059

[6] K. T. Kibret , C. Strugnell , K. Backholer , A. Peeters , T. K. Tegegne , and M. Nichols , “Life‐Course Trajectories of Body Mass Index and Cardiovascular Disease Risks and Health Outcomes in Adulthood: Systematic Review and Meta‐Analysis,” Obesity Reviews 25 (2024): e13695. 38226403 10.1111/obr.13695

[7] B. W. Jensen , J. Aarestrup , K. Blond , et al., “Childhood Body Mass Index Trajectories, Adult‐Onset Type 2 Diabetes, and Obesity‐Related Cancers,” Journal of the National Cancer Institute 115 (2023): 43–51. 36214627 10.1093/jnci/djac192 PMC9830482

[8] M. Dalmartello , J. Vermunt , E. Negri , F. Levi , and C. La Vecchia , “Adult Lifetime Body Mass Index Trajectories and Endometrial Cancer Risk,” BJOG: An International Journal of Obstetrics and Gynaecology 129 (2022): 1521–1529. 34962692 10.1111/1471-0528.17087

[9] K. Wang , X. Chen , T. A. Gerke , V. Y. Bird , H. K. Ghayee , and M. Prosperi , “BMI Trajectories and Risk of Overall and Grade‐Specific Prostate Cancer: An Observational Cohort Study Among Men Seen for Prostatic Conditions,” Cancer Medicine 7 (2018): 5272–5280. 30207080 10.1002/cam4.1747 PMC6198207

[10] H. L. Nguena Nguefack , M. G. Pagé , J. Katz , et al., “Trajectory Modelling Techniques Useful to Epidemiological Research: A Comparative Narrative Review of Approaches,” Clinical Epidemiology 12 (2020): 1205–1222. 33154677 10.2147/CLEP.S265287 PMC7608582

[11] O. Abdel‐Rahman , “Pre‐Diagnostic Body Mass Index Trajectory in Relationship to Lung Cancer Incidence and Mortality; Findings From the PLCO Trial,” Expert Review of Respiratory Medicine 13 (2019): 1029–1035. 31414914 10.1080/17476348.2019.1656532

[12] S. Arjani , P. F. Saint‐Maurice , S. Julián‐Serrano , G. Eibl , and R. Stolzenberg‐Solomon , “Body Mass Index Trajectories Across the Adult Life Course and Pancreatic Cancer Risk,” JNCI Cancer Spectrum 6 (2022): 6. 10.1093/jncics/pkac066 PMC9651977 36255251

[13] S. P. Kelly , H. Lennon , M. Sperrin , et al., “Body Mass Index Trajectories Across Adulthood and Smoking in Relation to Prostate Cancer Risks: The NIH‐AARP Diet and Health Study,” International Journal of Epidemiology 48 (2019): 464–473. 30376043 10.1093/ije/dyy219 PMC6469294

[14] D. You , D. Wang , Y. Wu , et al., “Associations of Genetic Risk, BMI Trajectories, and the Risk of Non‐Small Cell Lung Cancer: A Population‐Based Cohort Study,” BMC Medicine 20 (2022): 203. 35658861 10.1186/s12916-022-02400-6 PMC9169327

[15] M. J. Page , J. E. McKenzie , P. M. Bossuyt , et al., “The PRISMA 2020 Statement: An Updated Guideline for Reporting Systematic Reviews,” BMJ (Clinical Research Ed.) 372 (2021): n71. 10.1136/bmj.n71 PMC8005924 33782057

[16] GA, Wells , B, Shea , D, O'Connell , et al., The Newcastle–Ottawa Scale (NOS) for Assessing the Quality of Nonrandomised Studies in Metaanalyses.[Online], accessed in October 2024, https://www.ohri.ca/programs/clinical_epidemiology/oxford.asp.

[17] NP, Jewell Statistics for Epidemiology: Chapman and Hall/CRC 2003.

[18] J. P. Higgins and S. Green , eds., Cochrane Handbook for Systematic Reviews of Interventions, Version 5.1.0 (John Wiley & Sons, Ltd, 2011), http://handbook.cochrane.org.

[19] D. Nagin , Group‐Based Modeling of Development (Harvard University Press, 2005).

[20] H. Andruff , N. Carraro , A. Thompson , P. Gaudreau , and B. Louvet , “Latent Class Growth Modelling: A Tutorial,” Tutorial in Quantitative Methods for Psychology 5 (2009): 11–24.

[21] M. Busund , G. Ursin , E. Lund , T. Wilsgaard , and C. Rylander , “Trajectories of Body Mass Index in Adulthood and Risk of Subtypes of Postmenopausal Breast Cancer,” Breast Cancer Research 25 (2023): 130. 37898792 10.1186/s13058-023-01729-x PMC10612168

[22] P. W. Chiu , T. Yu , S. Kukreti , and C. Strong , “BMI Trajectory in Adulthood in Relation to All‐Cause and Cause‐Specific Mortality: A Retrospective Cohort Study in Taiwan,” PLoS ONE 18 (2023): e0295919. 38117791 10.1371/journal.pone.0295919 PMC10732409

[23] V. De Rubeis , M. Cotterchio , B. T. Smith , et al., “Trajectories of Body Mass Index, From Adolescence to Older Adulthood, and Pancreatic Cancer Risk; a Population‐Based Case‐Control Study in Ontario, Canada,” Cancer Causes Control 30 (2019): 955–966. 31230151 10.1007/s10552-019-01197-9 PMC6685923

[24] L. A. Gray , P. R. Breeze , and E. A. Williams , “BMI Trajectories, Morbidity, and Mortality in England: A Two‐Step Approach to Estimating Consequences of Changes in BMI,” Obesity (Silver Spring) 30 (2022): 1898–1907. 35920148 10.1002/oby.23510 PMC9546036

[25] M. Hoyt , Y. Song , S. Gao , J. O'Palka , and J. Zhang , “Prediagnostic BMI Trajectories in Relation to Pancreatic Cancer Risk in the Prostate, Lung, Colorectal, and Ovarian Cancer Screening Trial,” Obesity (Silver Spring) 30 (2022): 2275–2285. 36156459 10.1002/oby.23550 PMC9826088

[26] M. Song , W. C. Willett , F. B. Hu , et al., “Trajectory of Body Shape Across the Lifespan and Cancer Risk,” International Journal of Cancer 138 (2016): 2383–2395. 26704725 10.1002/ijc.29981 PMC5079685

[27] S. P. Kelly , B. I. Graubard , G. Andreotti , N. Younes , S. D. Cleary , and M. B. Cook , “Prediagnostic Body Mass Index Trajectories in Relation to Prostate Cancer Incidence and Mortality in the PLCO Cancer Screening Trial,” Journal of the National Cancer Institute 109 (2016): djw225. 27754927 10.1093/jnci/djw225 PMC5074530

[28] M. N. Kuchibhatla , G. G. Fillenbaum , W. E. Kraus , H. J. Cohen , and D. G. Blazer , “Trajectory Classes of Body Mass Index in a Representative Elderly Community Sample,” Journals of Gerontology. Series A, Biological Sciences and Medical Sciences 68 (2013): 699–704. 23089335 10.1093/gerona/gls215 PMC3660114

[29] C. Lavalette , E. Cordina Duverger , F. Artaud , et al., “Body Mass Index Trajectories and Prostate Cancer Risk: Results From the EPICAP Study,” Cancer Medicine 9 (2020): 6421–6429. 32639678 10.1002/cam4.3241 PMC7476828

[30] L. Luo , Y. Liu , Z. Wang , et al., “Relationship Between Prediagnostic Body Mass Index Trajectory and Colorectal Adenomas: An Analysis of the PLCO Cancer Screening Trial,” Annals of Translational Medicine 8 (2020): 815. 32793660 10.21037/atm-19-4634 PMC7396232

[31] D. C. Pedersen , J. Aarestrup , K. Blond , et al., “Trajectories of Body Mass Index Across the Lifecourse and Associations With Post‐Menopausal Breast Cancer by Estrogen Receptor Status,” Cancer Epidemiology 87 (2023): 102479. 37897969 10.1016/j.canep.2023.102479

[32] J. L. Petrick , S. P. Kelly , L. M. Liao , N. D. Freedman , B. I. Graubard , and M. B. Cook , “Body Weight Trajectories and Risk of Oesophageal and Gastric Cardia Adenocarcinomas: A Pooled Analysis of NIH‐AARP and PLCO Studies,” British Journal of Cancer 116 (2017): 951–959. 28196067 10.1038/bjc.2017.29 PMC5379141

[33] L. Su , M. Hendryx , M. Li , et al., “Body Size Over the Adult Life Course and the Risk of Colorectal Cancer Among Postmenopausal Women,” Public Health Nutrition 26 (2023): 1539–1548. 37199248 10.1017/S1368980023000988 PMC10410385

[34] E. Vallières , M. Mésidor , M. H. Roy‐Gagnon , H. Richard , and M. Parent , “General and Abdominal Obesity Trajectories Across Adulthood, and Risk of Prostate Cancer: Results From the PROtEuS Study, Montreal, Canada,” Cancer Causes & Control 32 (2021): 653–665. 33818663 10.1007/s10552-021-01419-z

[35] M. B. von Bonsdorff , T. Törmäkangas , T. Rantanen , et al., “Early Life Body Mass Trajectories and Mortality in Older Age: Findings From the Helsinki Birth Cohort Study,” Annals of Medicine 47 (2015): 34–39. 25307361 10.3109/07853890.2014.963664

[36] C. Watson , A. G. Renehan , and N. Geifman , “Associations of Specific‐Age and Decade Recall Body Mass Index Trajectories With Obesity‐Related Cancer,” BMC Cancer 21 (2021): 502. 33952200 10.1186/s12885-021-08226-4 PMC8097878

[37] B. Yang , J. L. Petrick , S. P. Kelly , B. I. Graubard , N. D. Freedman , and K. A. McGlynn , “Adiposity Across the Adult Life Course and Incidence of Primary Liver Cancer: The NIH‐AARP Cohort,” International Journal of Cancer 141 (2017): 271–278. 28411388 10.1002/ijc.30737 PMC5491533

[38] W. Yang , X. Zeng , J. L. Petrick , et al., “Body Mass Index Trajectories, Weight Gain and Risks of Liver and Biliary Tract Cancers,” JNCI Cancer Spectrum 6 (2022): 6. 10.1093/jncics/pkac056 PMC9406603 35960613

[39] Y. Yang , P. A. Dugué , B. M. Lynch , et al., “Trajectories of Body Mass Index in Adulthood and All‐Cause and Cause‐Specific Mortality in the Melbourne Collaborative Cohort Study,” BMJ Open 9 (2019): e030078. 10.1136/bmjopen-2019-030078 PMC6701564 31401610

[40] Y. Yang , B. M. Lynch , P. A. Dugué , et al., “Latent Class Trajectory Modeling of Adult Body Mass Index and Risk of Obesity‐Related Cancer: Findings From the Melbourne Collaborative Cohort Study,” Cancer Epidemiology, Biomarkers & Prevention 30 (2021): 373–379. 10.1158/1055-9965.EPI-20-0690 33268487

[41] R. Zheng , M. Du , B. Zhang , et al., “Body Mass Index (BMI) Trajectories and Risk of Colorectal Cancer in the PLCO Cohort,” British Journal of Cancer 119 (2018): 130–132. 29872147 10.1038/s41416-018-0121-y PMC6035226

[42] Z. Deng , M. Hajihosseini , J. X. Moore , et al., “Lifetime Body Weight Trajectories and Risk of Renal Cell Cancer: A Large U.S. Prospective Cohort Study,” Cancer Epidemiology, Biomarkers & Prevention 32 (2023): 1651–1659. 10.1158/1055-9965.EPI-23-0668 37624040

[43] J. Aarestrup , M. Gamborg , K. Tilling , L. G. Ulrich , T. I. Sørensen , and J. L. Baker , “Childhood Body Mass Index Growth Trajectories and Endometrial Cancer Risk,” International Journal of Cancer 140 (2017): 310–315. 27718528 10.1002/ijc.30464 PMC5132154

[44] S. Pati , W. Irfan , A. Jameel , S. Ahmed , and R. K. Shahid , “Obesity and Cancer: A Current Overview of Epidemiology, Pathogenesis, Outcomes, and Management,” Cancers 15 (2023): 485. 36672434 10.3390/cancers15020485 PMC9857053

[45] F. R. Greten and S. I. Grivennikov , “Inflammation and Cancer: Triggers, Mechanisms, and Consequences,” Immunity 51 (2019): 27–41. 31315034 10.1016/j.immuni.2019.06.025 PMC6831096

[46] B. D. Hopkins , M. D. Goncalves , and L. C. Cantley , “Obesity and Cancer Mechanisms: Cancer Metabolism,” Journal of Clinical Oncology 34 (2016): 4277–4283. 27903152 10.1200/JCO.2016.67.9712 PMC5562429

[47] T. W. Stone , M. McPherson , and L. Gail Darlington , “Obesity and Cancer: Existing and New Hypotheses for a Causal Connection,” eBioMedicine 30 (2018): 14–28. 29526577 10.1016/j.ebiom.2018.02.022 PMC5952217

[48] W. Zhong , X. Wang , Y. Wang , G. Sun , J. Zhang , and Z. Li , “Obesity and Endocrine‐Related Cancer: The Important Role of IGF‐1,” Frontiers in Endocrinology 14 (2023): 1093257. 36755926 10.3389/fendo.2023.1093257 PMC9899991

[49] L. Crudele , E. Piccinin , and A. Moschetta , “Visceral Adiposity and Cancer: Role in Pathogenesis and Prognosis,” Nutrients 13 (2021): 2101. 34205356 10.3390/nu13062101 PMC8234141

[50] C. B. Lagarde , J. Kavalakatt , M. C. Benz , et al., “Obesity‐Associated Epigenetic Alterations and the Obesity‐Breast Cancer Axis,” Oncogene 43 (2024): 763–775. 38310162 10.1038/s41388-024-02954-0 PMC13126611

[51] N. Mohammadian Khonsari , E. Shahrestanaki , A. Ehsani , et al., “Association of Childhood and Adolescence Obesity With Incidence and Mortality of Adulthood Cancers. A Systematic Review and Meta‐Analysis,” Frontiers in Endocrinology 14 (2023): 1069164. 36742402 10.3389/fendo.2023.1069164 PMC9892178

[52] Y. H. Park , H. W. Moon , H. J. Cho , et al., “Cumulative Obesity Exposure Increases the Risk of Kidney Cancer: A Longitudinal Nationwide Cohort Study,” American Journal of Cancer Research 11 (2021): 5016–5026. 34765308 PMC8569344

[53] P. Peretti‐Watel , L. Fressard , A. Bocquier , and P. Verger , “Perceptions of Cancer Risk Factors and Socioeconomic Status. A French Study,” Preventive Medicine Reports 3 (2016): 171–176. 27419011 10.1016/j.pmedr.2016.01.008 PMC4929177

[54] H. Arem and E. Loftfield , “Cancer Epidemiology: A Survey of Modifiable Risk Factors for Prevention and Survivorship,” American Journal of Lifestyle Medicine 12 (2018): 200–210. 30202392 10.1177/1559827617700600 PMC6124966

[55] Q. Wang , X. Shao , Y. Zhang , et al., “Role of Tumor Microenvironment in Cancer Progression and Therapeutic Strategy,” Cancer Medicine 12 (2023): 11149–11165. 36807772 10.1002/cam4.5698 PMC10242329

[56] A. S. Yende and D. Sharma , “Obesity, Dysbiosis and Inflammation: Interactions That Modulate the Efficacy of Immunotherapy,” Frontiers in Immunology 15 (2024): 1444589. 39253073 10.3389/fimmu.2024.1444589 PMC11381382

[57] G. Li , Y. Wan , A. Jiao , et al., “Breaking Boundaries: Chronic Diseases and the Frontiers of Immune Microenvironments,” Medicina Research 1 (2025): 62–102, https://ora.ox.ac.uk/objects/uuid:238d7a6d‐a64f‐4ee6‐882b‐f6a1a458f438.

[58] Y. Fang , Y. Kong , G. Rong , Q. Luo , W. Liao , and D. Zeng , “Systematic Investigation of Tumor Microenvironment and Antitumor Immunity With IOBR,” Le Médecin de Réserve 1 (2025): 136–140, 10.1002/mdr2.70001.

[59] C. Biray Avci , B. Goker Bagca , M. Nikanfar , L. S. Takanlou , M. S. Takanlou , and A. Nourazarian , “Tumor Microenvironment and Cancer Metastasis: Molecular Mechanisms and Therapeutic Implications,” Frontiers in Pharmacology 15 (2024): 1442888. 39600368 10.3389/fphar.2024.1442888 PMC11588459

[60] H. Pópulo , J. M. Lopes , and P. Soares , “The mTOR Signalling Pathway in Human Cancer,” International Journal of Molecular Sciences 13 (2012): 1886–1918. 22408430 10.3390/ijms13021886 PMC3291999

[61] C. Magaway , E. Kim , and E. Jacinto , “Targeting mTOR and Metabolism in Cancer: Lessons and Innovations,” Cells 8 (2019): 8. 10.3390/cells8121584 PMC6952948 31817676

[62] I. Glassman , N. Le , A. Asif , et al., “The Role of Obesity in Breast Cancer Pathogenesis,” Cells 12 (2023): 12. 10.3390/cells12162061 PMC10453206 37626871

[63] S. S. Mohanty and P. K. Mohanty , “Obesity as Potential Breast Cancer Risk Factor for Postmenopausal Women,” Genes & Disease 8 (2021): 117–123. 10.1016/j.gendis.2019.09.006 PMC8099684 33997158

[64] L. A. Torres‐de la Roche , I. Steljes , W. Janni , T. W. P. Friedl , and R. L. De Wilde , “The Association Between Obesity and Premenopausal Breast Cancer According to Intrinsic Subtypes ‐ A Systematic Review,” Geburtshilfe Und Frauenheilkunde 80 (2020): 601–610. 32565550 10.1055/a-1170-5004 PMC7299685

[65] A. Wolk , C. S. Mantzoros , S. O. Andersson , et al., “Insulin‐Like Growth Factor 1 and Prostate Cancer Risk: A Population‐Based, Case‐Control Study,” Journal of the National Cancer Institute 90 (1998): 911–915. 9637140 10.1093/jnci/90.12.911

[66] B. A. Adesunloye , “Mechanistic Insights Into the Link Between Obesity and Prostate Cancer,” International Journal of Molecular Sciences 22 (2021): 3935. 33920379 10.3390/ijms22083935 PMC8069048

[67] M. E. Meunier , Y. Neuzillet , J. F. Dreyfus , et al., “PSA and Obesity Among Men With Localized Prostate Cancer: Results of the Androcan Study,” World Journal of Urology 39 (2021): 2945–2951. 33521883 10.1007/s00345-020-03557-6

[68] L. L. Bañez , R. J. Hamilton , A. W. Partin , et al., “Obesity‐Related Plasma Hemodilution and PSA Concentration Among Men With Prostate Cancer,” Journal of the American Medical Association 298 (2007): 2275–2280. 18029831 10.1001/jama.298.19.2275

[69] K. Sweatt , W. T. Garvey , and C. Martins , “Strengths and Limitations of BMI in the Diagnosis of Obesity: What Is the Path Forward?,” Current Obesity Reports 13 (2024): 584–595. 38958869 10.1007/s13679-024-00580-1 PMC11306271

[70] Y. Wu , D. Li , and S. H. Vermund , “Advantages and Limitations of the Body Mass Index (BMI) to Assess Adult Obesity,” International Journal of Environmental Research and Public Health 21 (2024): 757. 38929003 10.3390/ijerph21060757 PMC11204233

[71] S. Parra‐Soto , J. Boonpor , N. Lynskey , et al., “Association Between Visceral Adiposity Index and Cancer Risk in the UK Biobank Cohort,” Cancer 131 (2025): e35576. 39361532 10.1002/cncr.35576 PMC11694164

[72] E. A. Silveira , N. Kliemann , M. Noll , N. Sarrafzadegan , and C. de Oliveira , “Visceral Obesity and Incident Cancer and Cardiovascular Disease: An Integrative Review of the Epidemiological Evidence,” Obesity Reviews 22 (2021): e13088. 32692447 10.1111/obr.13088 PMC7757158

[73] F. Rubino , D. E. Cummings , R. H. Eckel , et al., “Definition and Diagnostic Criteria of Clinical Obesity,” Lancet Diabetes & Endocrinology 13 (2025): 221–262. 39824205 10.1016/S2213-8587(24)00316-4 PMC11870235

[74] F. Q. Nuttall , “Body Mass Index: Obesity, BMI, and Health: A Critical Review,” Nutrition Today 50 (2015): 117–128. 27340299 10.1097/NT.0000000000000092 PMC4890841

[75] Z. Li , S. Daniel , K. Fujioka , and D. Umashanker , “Obesity Among Asian American People in the United States: A Review,” Obesity (Silver Spring) 31 (2023): 316–328. 36695056 10.1002/oby.23639 PMC10108164

[76] Y. Okawa , T. Mitsuhashi , and T. Tsuda , “The Asia‐Pacific Body Mass Index Classification and New‐Onset Chronic Kidney Disease in Non‐Diabetic Japanese Adults: A Community‐Based Longitudinal Study From 1998 to 2023,” Biomedicine 13 (2025): 373. 10.3390/biomedicines13020373 PMC11853003 40002785

[77] W. E. Consultation , “Appropriate Body‐Mass Index for Asian Populations and Its Implications for Policy and Intervention Strategies,” Lancet 363 (2004): 157–163. 14726171 10.1016/S0140-6736(03)15268-3

[78] Y. Kim , S. M. Jeong , B. Yoo , B. Oh , and H. C. Kang , “Associations of Smoking With Overall Obesity, and Central Obesity: A Cross‐Sectional Study From the Korea National Health and Nutrition Examination Survey (2010‐2013),” Epidemiology Health 38 (2016): e2016020. 27221478 10.4178/epih.e2016020 PMC4967909

[79] P. de Groot and R. F. Munden , “Lung Cancer Epidemiology, Risk Factors, and Prevention,” Radiologic Clinics of North America 50 (2012): 863–876. 22974775 10.1016/j.rcl.2012.06.006

[80] M. G. Prado , L. G. Kessler , M. A. Au , et al., “Symptoms and Signs of Lung Cancer Prior to Diagnosis: Case‐Control Study Using Electronic Health Records From Ambulatory Care Within a Large US‐Based Tertiary Care Centre,” BMJ Open 13 (2023): e068832. 10.1136/bmjopen-2022-068832 PMC10124310 37080616

[81] M. Zigman Suchsland , L. Kowalski , H. A. Burkhardt , et al., “How Timely Is Diagnosis of Lung Cancer? Cohort Study of Individuals With Lung Cancer Presenting in Ambulatory Care in the United States,” Cancers (Basel) 14 (2022): 14. 10.3390/cancers14235756 PMC9740627 36497238

[82] V. K. Lam , S. M. Bentzen , P. Mohindra , et al., “Obesity Is Associated With Long‐Term Improved Survival in Definitively Treated Locally Advanced Non‐Small Cell Lung Cancer (NSCLC),” Lung Cancer 104 (2017): 52–57. 28213000 10.1016/j.lungcan.2016.11.017

[83] A. M. Wood , H. Jonsson , G. Nagel , et al., “The Inverse Association of Body Mass Index With Lung Cancer: Exploring Residual Confounding, Metabolic Aberrations and Within‐Person Variability in Smoking,” Cancer Epidemiology, Biomarkers & Prevention 30 (2021): 1489–1497. 10.1158/1055-9965.EPI-21-0058 34162656

[84] M. E. Ramos‐Nino , “The Role of Chronic Inflammation in Obesity‐Associated Cancers,” ISRN Oncology 2013 (2013): 697521. 23819063 10.1155/2013/697521 PMC3683483

[85] E. Vélez‐Bonet , K. Gumpper‐Fedus , and Z. Cruz‐Monserrate , “Exploring the Role of Hyperinsulinemia in Obesity‐Associated Tumor Development,” Cancer Research 84 (2024): 351–352. 38095504 10.1158/0008-5472.CAN-23-3932 PMC11472301

[86] Y. Yang , M. Chen , and W. Yang , “Mediation Effects of Metabolites and Sex Hormones on the Relationship Between Body Mass Index and Breast Cancer: Mendelian Randomization Analysis and Mediation Analysis,” Frontiers in Oncology 14 (2024): 1449956. 39640279 10.3389/fonc.2024.1449956 PMC11617525

[87] E. Tokunaga , Y. Hisamatsu , K. Tanaka , et al., “Molecular Mechanisms Regulating the Hormone Sensitivity of Breast Cancer,” Cancer Science 105 (2014): 1377–1383. 25155268 10.1111/cas.12521 PMC4462367

[88] P. Bhardwaj , C. C. Au , A. Benito‐Martin , et al., “Estrogens and Breast Cancer: Mechanisms Involved in Obesity‐Related Development, Growth and Progression,” Journal of Steroid Biochemistry and Molecular Biology 189 (2019): 161–170. 30851382 10.1016/j.jsbmb.2019.03.002 PMC6502693

[89] T. P. T. Tran , N. M. Luu , T. T. Bui , M. Han , M. K. Lim , and J. K. Oh , “Trajectory of Physical Activity Frequency and Cancer Risk: Findings From a Population‐Based Cohort Study,” European Review of Aging and Physical Activity 20 (2023): 4. 36890434 10.1186/s11556-023-00316-5 PMC9996897

[90] C. N. Christopher , C. E. Matthews , P. F. Saint‐Maurice , and S. K. Keadle , “Impact of Moderate‐Vigorous Physical Activity Trajectories on Colon Cancer Risk Over the Adult Life Course,” Cancer Epidemiology, Biomarkers & Prevention 32 (2023): 30–36. 10.1158/1055-9965.EPI-22-0768 PMC9839573 36306403

[91] J. L. Yin , Y. Z. Li , R. Wang , et al., “Dietary Patterns and Risk of Multiple Cancers: Umbrella Review of Meta‐Analyses of Prospective Cohort Studies,” American Journal of Clinical Nutrition 121 (2025): 213–223. 39603532 10.1016/j.ajcnut.2024.11.020

[92] Y. Zhang and E. L. Giovannucci , “What to Eat for Cancer Prevention: The Total Dietary Pattern as a Combination Treatment for Prevention,” Cancer Journal 30 (2024): 307–312. 39312450 10.1097/PPO.0000000000000741

[93] X. Diao , Y. Ling , Y. Zeng , et al., “Physical Activity and Cancer Risk: A Dose‐Response Analysis for the Global Burden of Disease Study 2019,” Cancer Commun (Lond) 43 (2023): 1229–1243. 37743572 10.1002/cac2.12488 PMC10631483

[94] S. Li , Y. He , J. Liu , et al., “An Umbrella Review of Socioeconomic Status and Cancer,” Nature Communications 15 (2024): 9993. 10.1038/s41467-024-54444-2 PMC11574020 39557933

[95] M. Sun , M. da Silva , T. Bjørge , et al., “Body Mass Index and Risk of Over 100 Cancer Forms and Subtypes in 4.1 Million Individuals in Sweden: The Obesity and Disease Development Sweden (ODDS) Pooled Cohort Study,” Lancet Regional Health ‐ Europe 45 (2024): 101034. 39253735 10.1016/j.lanepe.2024.101034 PMC11381908

[96] S. Capewell and A. Capewell , “An Effectiveness Hierarchy of Preventive Interventions: Neglected Paradigm or Self‐Evident Truth?,” Journal of Public Health (Oxford, England) 40 (2018): 350–358. 28525612 10.1093/pubmed/fdx055

[97] C. Wagner , C. Carmeli , J. Jackisch , et al., “Life Course Epidemiology and Public Health,” Lancet Public Health 9 (2024): e261–e269. 38553145 10.1016/S2468-2667(24)00018-5

[98] https://www.canceraustralia.gov.au/resources/position‐statements/lifestyle‐risk‐factors‐and‐primary‐prevention‐cancer/lifestyle‐risk‐0?utm_source=chatgpt.com.

[99] C. L. Rock , C. Thomson , T. Gansler , et al., “American Cancer Society Guideline for Diet and Physical Activity for Cancer Prevention,” CA: A Cancer Journal for Clinicians 70 (2020): 245–271. 32515498 10.3322/caac.21591

[100] Be a Healthy Weight? https://www.wcrf.org/research‐policy/evidence‐for‐our‐recommendations/be‐a‐healthy‐weight/?utm_source=chatgpt.com.

[101] American Cancer Society Guideline for Diet and Physical Activity for Cancer Prevention. chrome‐extension, accessed September 22, 2025, https://iris.who.int/bitstream/handle/10665/43575/9241547111_eng.pdf?sequence=1. 10.3322/caac.21591 32515498

