# Supplementary material for: Life Course Trajectories of Body Mass Index and Risk of Cancer in Adulthood: Systematic Review and Meta‐Analysis
Source: Obes Rev. 2026 Mar 5;27(8):e70114. doi: 10.1111/obr.70114 (PMC13371409; doi:10.1111/obr.70114)

Supporting Information

Title: Life-course trajectories of body mass index and risk of cancer in adulthood: Systematic review and meta-analysis

Running title: BMI trajectories and risk of cancer

Samira Behboudi-Gandevani [1], Tommy Haugan [2], Nayla Cristina Do Vale Moreira [1], Ellen Christin Arntzen [1], Moira Strand Hutchinson [3], Melanie Nichols [4], Razieh Bidhendi-Yarandi [5,6]

Affiliations:

1 Faculty of Nursing and Health Sciences, Nord University, Bodø, Norway
2 Faculty of Nursing and Health Sciences, Nord University, Levanger, Norway
3 Valnesfjord Centre for Health sports and Rehabilitation, Fauske, Norway
4 Global Centre for Preventive Health and Nutrition, Institute for Health Transformation, Faculty of Health, Deakin University, Geelong, VIC, Australia
5 Department of Biostatistics and Epidemiology, University of Social Welfare and Rehabilitation Sciences, Tehran, Iran.
6 Neuromusculoskeletal Rehabilitation Research Center, University of Social Welfare and Rehabilitation Sciences, Tehran, Iran.

*Corresponding Author: Samira Behboudi-Gandevani, PhD, Associate Professor
Email: samira.behboudi-gandevani@nord.no
Tel: +47 755 17 000
Mail address: 8049 Bodø, Norway
Post box: 1490

Supplementary Table 1. Quality assessment of the included studies using the Newcastle–Ottawa Quality Assessment Scale for cohort studies.

	SELECTION				COMPARABILITY	Outcome			Total scores
**Author, Year**	Representativeness of the exposed cohort<br>a) Truly representative *<br>b) Somewhat representative (one star)<br>c) Selected group<br>d) No description of the derivation of the cohort	Selection of the non-exposed cohort<br>a) Drawn from the same community as the exposed cohort *<br>b) Drawn from a different source<br>c) No description of the derivation of the non exposed cohort	Ascertainment of exposure<br>a) Secure record (e.g., surgical record) *<br>b) Structured interview *<br>c) Written self report<br>d) No description<br>e) Other	Demonstration that outcome of interest was not present at start of study<br>a) Yes *<br>b) No	Comparability of cohorts on the basis of the design or analysis controlled for confounders<br>a) The study controls for age, and BMI *<br>b) Study controls for other factors (list) *<br>c) Cohorts are not comparable on the basis of the design or analysis controlled for confounders	Assessment of outcome<br>a) Independent blind assessment) *<br>b) Record linkage *<br>c) Self report<br>d) No description<br>e) Other	2) Was follow-up long enough for outcomes to occur<br>a) Yes *<br>b) No	Adequacy of follow-up of cohorts<br>a) Complete follow up- all subject accounted for *<br>b) Subjects lost to follow up unlikely to introduce bias- number lost less than or equal to 20% or description of those lost suggested no different from those followed. *<br>c) Follow up rate less than 80% and no description of those lost<br>d) No statement	
Abdel-Rahman O. 2019 [1]	Population-based sample from PLCO trial *	Non-exposed cohort drawn from the same population *	Self-reported BMI for early life; objective for baseline *	Lung cancer-free cohort at baseline *	Adjusted for sex, smoking, race, and family history **	Cancer outcomes rigorously assessed *	Mean follow-up of 11.25 years*	Large cohort with adequate follow-up*	9*
Arjani S, et al. 2022 [5]	The cohort is from the National Institutes of Health-AARP Diet and Health Study, a large population-based sample of AARP members aged 50-71 years, making it a truly representative cohort *	The non-exposed cohort (those without pancreatic ductal adenocarcinoma) was drawn from the same population.*	BMI data was self-reported for earlier life periods (ages 18, 35, 50) and measured at baseline (ages 50-71). Self-report may introduce some bias, but height and weight were captured with good consistency.*	All participants were cancer-free at baseline.*	Adjusted for important confounders such as sex, smoking status, alcohol consumption, and diabetes. BMI trajectories were also adjusted for other known pancreatic cancer risk factors.**	Cancer outcomes (pancreatic ductal adenocarcinoma) were confirmed using record linkage with cancer registries and the US National Death Index, ensuring rigorous assessment.*	Follow-up was over 15 years.*	The cohort had a complete follow-up with all subjects accounted for, and any potential loss to follow-up was described as unlikely to introduce bias.*	9*
Busund M, et al. 2023 [14]	The study cohort is considered truly representative as it includes a large sample	The non-exposed cohort was drawn from the same community,.*	Exposure ascertainment is based on secure records, *	The study confirmed that the outcome of interest was not	The study controlled for age and BMI and other other relevant factors such as age at menarche, parity, history of breast	The outcome assessment was conducted independently *	Follow-up duration was sufficient for outcomes to occur, with a	There was complete follow-up of subjects*	9*

	from the general population in Norway.*			present at the start, *	cancer in the mother, physical activity, smoking status, and MHT use.**		mean follow-up time of 14.9 years*		
Chiu PW, et al. 2023 [25]	Truly representative *	Drawn from the same community as the exposed cohort *	Secure record (measured BMI) *	Yes (excluded those with cancer, stroke at baseline) *	Study controls for age and BMI and other factors (smoking, alcohol, education, physical activity) **	Record linkage (National Death Registry) *	Yes (mean follow-up 16.8 years) *	The cohort had a complete follow-up. Subjects lost to follow up unlikely to introduce bias *	9*
De Rubeis V, et al. 2019 [42]	The study recruited cases from the Ontario Pancreas Cancer Study, which is a population-based registry, suggesting a representative sample of individuals diagnosed with pancreatic cancer *	Controls were recruited from the Ontario Cancer Risk Factor Study using a modified random digit dialing procedure, ensuring they were from the same community*	BMI was assessed through self-reported height and weight at multiple time points, which is a structured approach, although it is self-reported *	**Yes,** The study design includes cases diagnosed with pancreatic cancer, indicating that they did not have the outcome at the start of the study *	The study adjusted for age, sex, and other potential confounders including race, alcohol consumption, smoking, diet, physical activity, and family history of pancreatic cancer in the analysis.**	The Ontario Cancer Registry was used for identifying cases, indicating a reliable method of outcome ascertainment.*	**Not mentioned in study** (0 stars)	The cohort had a complete follow-up. *	8*
Gray LA, et al. 2022 [61]	The study used data from the English Longitudinal Study of Aging (ELSA) which includes a large, nationally representative sample of individuals aged 50 and older *	Participants from the Health Survey for England were included, which covered both exposed and non-exposed groups in the same population. *	BMI was measured by nurses during several waves *	**Yes** – The study considered the baseline health status of participants, ensuring that outcome morbidities were tracked over time *	The analysis adjusted for key confounders such as age, sex, smoking, and marital status, ethnicity, smoking status, and socioeconomic status **	Mortality data were linked to the National Health Service central register, and morbidities were self-reported with regular surveys. While self-report has limitations, medical records supported the mortality data *	**Yes** – The study followed participants over a 17-year period *	Around 85% of eligible participants participated in each wave, and detailed reporting on those lost to follow-up was included. *	9*
Hoyt M, et al. 2022 [77]	The study used a large, nationally representative sample from the Prostate, Lung, Colorectal, and Ovarian (PLCO) Cancer Screening Trial with over 145,000 participants. *	Both exposed and non-exposed individuals were drawn from the same PLCO cohort. *	Weight and height data were self-reported retrospectively but complemented with subsequent medical records for cancer diagnosis *	**Yes** – Participants were cancer-free at the beginning of the study *	The analysis adjusted for essential confounders, including age, sex, smoking, and family history of pancreatic cancer, race, marital status, and diabetes**.	Cancer outcomes were ascertained through medical records, death certificates, and pathology reports, ensuring accurate outcome data	The median follow-up was 12 years *	The study accounted for loss to follow-up, and exclusions were reported clearly, with a low dropout rate *	9*
Song M, et al. 2016 [87]	Truly representative *	Drawn from the same community as the exposed cohort*	Written self report (0 star)	Yes *	The study controls for age and BMI and other multiple potential confounders **	Record linkage *	Yes *	Subjects lost to follow up unlikely to introduce bias * (Follow-up rates were 95.4% in	8*

								NHS and 95.9% in HPFS)	
Kelly SP, et al. 2019 [94]	The study included a large cohort of 62,565 postmenopausal women from the Nurses' Health Study, representing a broad population of female health professionals *	All participants were drawn from the same Nurses' Health Study cohort*	Dietary information was collected through self-administered food frequency questionnaires (0 star)	Yes * Women with a history of cancer at baseline were excluded from the study.	Analyses were adjusted for multiple potential confounders including age, BMI, physical activity, smoking, and other relevant factors **	Cancer outcomes were confirmed through medical record review by study physicians. *	Yes * The study had a long follow-up period of up to 22 years (1984-2006)	The follow-up rate was high (95.4% of person-time), Subjects lost to follow up unlikely to introduce bias *	8*
Kelly SP, et al. 2016 [95]	The study included 69,873 men from the PLCO Cancer Screening Trial, representing a large cohort from the general population *	All participants were drawn from the same PLCO Cancer Screening Trial cohort. *	BMI was calculated based on self-reported height and weight at different ages. (0 star)	Yes * Men with prior history of cancer were excluded from the study.	Analyses were adjusted for multiple potential confounders including screening arm, family history, race, study center, education, marital status, smoking status, diabetes, myocardial infarction, and PSA history **	Cancer outcomes were ascertained through active follow-up and linkage to the National Death Index*	Yes * The study had a median follow-up of 11.5 years, which is sufficient for prostate cancer outcomes to occur	While specific follow-up rates were not provided, the study used active follow-up methods and linkage to national registries, suggesting high completeness. So Subjects lost to follow up unlikely to introduce bias *	8*
Kuchibhatla MN, et al. 2013 [105]	The study included a stratified random sample of community-based residents aged 65 years and older from five adjacent counties in North Carolina. So Somewhat representative *	All participants were drawn from the same community-based sample*	BMI was calculated from self-reported height and weight, though there was good agreement with measured values at one time point (0 star)	Cancer free at baseline *	Analyses were adjusted for multiple potential confounders including race, sex, ge, sex, education, stroke, heart disease, hypertension, diabetes, cognitive impairment, and depression.**	Health status and BMI were mostly based on self-reported data (0 star)	Yes * The study had a 10-year follow-up period	At the final wave, 9.3% of survivors dropped out, which is less than 20%, so subjects lost to follow up unlikely to introduce bias *	7*
Luo L, et al. 2020 [126]	Participants were selected from the intervention arm of the PLCO cancer screening trial, which provides a large cohort. However, the sample is primarily non-Hispanic white, so it may not be fully representative of the general population. So Somewhat representative *	The non-exposed cohort was also part of the PLCO trial *	BMI data was self-reported through recall at different ages (0 star)	Participants were confirmed to be free of colorectal adenomas at the time of their first colonoscopy or sigmoidoscopy *	The study controlled for key factors such as age, race, gender, smoking status, and education level **	Colorectal adenomas were confirmed histologically after endoscopic biopsy *	Yes* The study had a median follow-up time of 12.4 years *	Most participants were followed up adequately, and there was no significant loss of follow-up reported *	8*
Pedersen DC, et al. 2023 [160]	The study cohort consisted of Danish women from the Copenhagen School Health Records Register (CSHRR)	Both exposed and non-exposed women came from the same population *	BMI in childhood was measured by trained	The study excluded women diagnosed with	The study controlled for important confounders, including age at menarche, number of	Post-menopausal breast cancer diagnoses and estrogen receptor	Yes * The follow-up duration up to 23 years	The study maintained a high follow-up rate, and any losses to	9*

	and the Diet, Cancer, and Health (DCH) cohort So Somewhat representative *		professionals during school health examinations, while BMI in adulthood was based on self-reported weight *	breast cancer prior to follow-up *	pregnancies, breastfeeding duration, education, hormone replacement therapy (HRT) use, smoking, alcohol intake, and physical activity **	status were determined from the Danish Breast Cancer Cooperative Group database *		follow-up were unlikely to introduce significant bias *	
Petrick JL, et al. 2017 [165]	The cohort includes a large sample from multiple US states and metropolitan areas *	Non-exposed individuals were from the same source population as exposed*	BMI and weight data were self-reported by participants (0 star)	Individuals with prior cancer diagnoses were excluded *	Adjusted for sex, race, smoking, education and other potential confounders **	Outcomes ascertained through cancer registry linkage and medical record verification *	Yes * Follow-up through 2009-2011	High follow-up rate through registry linkage and medical records *	8*
Su L, et al. 2023 [194]	The study included postmenopausal women from 40 clinical centers across the US, which provides some representativeness, but may not fully represent all postmenopausal women *	Both exposed and non-exposed groups were drawn from the same WHI-OS cohort *	BMI at ages 18, 35, and 50 was based on self-reported height and weight (0 star)	Women with a history of cancer at baseline were excluded *	The study adjusted for multiple relevant confounders including age, race/ethnicity, education, physical activity, smoking, alcohol use, diet quality, family history, screening history, and other health factors **	Colorectal cancer cases were initially self-reported but then confirmed through medical record review *	Yes* The average follow-up was 15.8 years	hile not explicitly stated, the WHI studies generally have high follow-up rates. The large sample size (79,034) also suggests adequate follow-up *	8*
von Bonsdorff MB, et al. 2015 [213]	The study cohort consisted of individuals born in Helsinki between 1934 and 1944 *	Drawn from the same community *	Data on weight and height were extracted from detailed health care records *	Free of outcome at start of study *	The analysis was controlled for factors such as age, socio-economic status in childhood and adulthood, smoking, alcohol consumption, physical activity, and adult BMI **	Mortality data were obtained from the Finnish National Death Register *	Yes * The follow-up spanned from 2000 to 2010	he study reports on data from a significant portion of the cohort (around 65.6% response rate to a postal questionnaire and mortality data for 9.3% of participants) *	9*
Wang K, et al. 2018 [216]	The study utilized participants from the Prostate, Lung, Colorectal, and Ovarian (PLCO) Cancer Screening Trial, which involved a large, diverse cohort across multiple screening centers in the U.S. *	The non-exposed participants were drawn from the same PLCO cohort *	BMI was calculated based on both self-reported data at specific ages (0 star)	The participants were initially recruited for cancer screening, and only incident cancer cases were considered *	The study controlled baseline BMI, smoking status, diabetes, heart conditions, education level, race, and family history of cancer. For women, additional adjustments were made for hormone replacement therapy (HRT). **	Cancer incidence was confirmed through medical records, cancer registries, and the National Death Index *	Yes * The follow-up period extended up to 2010	The study had a high response rate (87% of the original cohort) and included a large number of participants, minimizing potential bias from loss to follow-up *	9*

Watson C, et al. 2021 [219]	The study uses data from the PLCO Cancer Screening Trial, which includes a large sample *	All participants are from the PLCO trial *	BMI data was collected through self-reported questionnaires (0 star)	The study focuses on incident cancer risk*	The study adjusts for multiple factors including smoking, diabetes, heart conditions, education, race, family history, NSAID use, and HRT status in women **	Cancer incidence was monitored through questionnaires and verified through medical records and cancer registries *	Not mentioned exactly (0 star)	The study used passive linkage after 2010, which likely minimized loss to follow-up.*	7*
Yang B, et al. 2017 [231]	The study uses data from the NIH-AARP Diet and Health Study, which includes a large sample, so Somewhat representative *	All participants are from the NIH-AARP cohort *	BMI was calculated based on self-reported weight and height at different ages. (0 star)	The study excluded participants with self-reported prevalent cancer at baseline *	The study adjusts for multiple factors including sex, age, physical activity, history of diabetes, alcohol drinking, smoking status, and consumption of red meat **	Cancer diagnoses were ascertained through linkage to state cancer registries *	Yes * The study had a mean follow-up of 11.9 years	The study does not provide information on the completeness of follow-up or loss to follow-up rates. (0 star)	7*
Yang W, et al. 2022 [233]	The cohort was drawn from the Prostate, Lung, Colorectal, and Ovarian (PLCO) Cancer Screening Trial, which included 154,887 participants from multiple diverse study sites across the U.S. *	Both exposed and non-exposed individuals were drawn from the same PLCO cohort *	BMI was calculated based on self-reported weight at ages 20, 50, and at enrollment, collected through structured questionnaires (0 star)	Participants with a prior cancer diagnosis (except non-melanoma skin cancer) were excluded from the study *	The analysis was adjusted for key confounders, including age, sex, race, BMI, smoking status, education level, family history of liver and biliary tract cancers, and aspirin use, diabetes, hypertension, and alcohol consumption**	Liver and biliary tract cancer cases were ascertained through self-reports, state cancer registries, and linkage to the National Death Index *	Yes * The median follow-up period was 15.9 years	The study had a high follow-up rate, and there were no significant indications that loss to follow-up introduced bias *	8*
Yang Y, et al. 2019 [234]	The study used a large, population-based cohort of over 29,000 participants from Melbourne *	The control (non-exposed) group was selected from the same population as the exposed group *	Body mass index (BMI) was derived using both measured and self-reported weight data *	Participants diagnosed with cancer before the last BMI measurement or with extreme BMI values were excluded from the analysis *	The study controlled for multiple confounders, including age, height, sex, country of birth, socioeconomic status, and education level **	utcomes (deaths) were ascertained through record linkage to the Victorian Registry of Births, Deaths, and Marriages, and the National Death Index *	Yes * The study had an extended follow-up period for mortality outcomes (up to 2017 for all-cause mortality and 2013 for cause-specific mortality)	The study had a follow-up rate of 88% in 1995–1998 and 68% in 2003–2007, and loss to follow-up was unlikely to introduce significant bias *	9*
Yang Y, et al. 2021 [235]	The cohort was large (n=30,377) and drawn from the Melbourne Collaborative Cohort Study*	The non-exposed cohort was selected from the same population as the exposed group *	(BMI) was measured at multiple time points *	Participants diagnosed with invasive cancer before the last BMI measurement were excluded from the analysis *	The study adjusted for various confounders, including age, sex, height, education, country of birth, smoking status, and socioeconomic status **	Cancer outcomes were ascertained using data from the Victorian Cancer Registry and the Australian Cancer Database *	NM (0 star)	The study had follow-up rates of 88% at the first follow-up (1995–1998) and 68% at the second follow-up (2003–2007), and loss to follow-	8*

								up was unlikely to introduce bias *	
You D, et al. 2022 [237]	The study utilized a large cohort (138,110 participants) from the Prostate, Lung, Colorectal, and Ovarian (PLCO) Cancer Screening Trial, a population-based cohort recruited from various centers across the U.S. *	Both exposed (with BMI trajectories) and non-exposed groups were drawn from the same population *	BMI at different life stages was ascertained through self-reported questionnaires (0 star)	Participants with a history of cancer before enrollment were excluded from the analysis *	The study adjusted for key confounders including age, sex, smoking status, race, family history of lung cancer, education, and other factors **	The outcome (non-small cell lung cancer diagnosis) was ascertained through medical records, National Death Index, and self-reported annual questionnaires *	NM (0 star)	The study reported comprehensive follow-up with minimal loss to follow-up, and dropouts were unlikely to introduce significant bias *	7*
Aarestrup J, et al. 2017 [245]	The cohort included all girls from the Copenhagen School Health Records Register born 1930-1989 with available data *	All subjects came from the same population-based cohort *	Height and weight were measured by medical professionals during mandatory school health examinations. *	Women with endometrial cancer prior to 1978 or age 18 were excluded *	The study controlled for age (used as time scale) and birth cohort (stratified analyses)*	Endometrial cancer outcomes were identified through linkage to the national Danish Cancer Registry *	Yes * Follow-up was until 2012 (4.1 million person-years)	Linkage to national registers allowed for complete follow-up *	9*
Zheng R, et al. 2018 [244]	The study included 139,229 subjects from the Prostate, Lung, Colorectal, and Ovarian (PLCO) Cancer Screening Trial, which is a large population-based cohort. *	All subjects were from the same PLCO cohort. *	BMI was ascertained from self-reported questionnaires (0 star)	At study entry, subjects were not diagnosed with colorectal cancer. *	The study controlled for age, sex, race, and cigarette smoking status **	CRC cases were ascertained by self-reported annual questionnaires and linkage to the National Death Index, and were histologically confirmed via medical record reviews *	Yes * The study had a 13-year follow-up period	The study used linkage to national registers, suggesting complete follow-up *	8*
Deng Z, et al. 2023 [246]	The study drew from the PLCO Cancer Screening Trial, a large, randomized, population-based cohort, which included over 138,000 participants from various centers in the U.S. *	The non-exposed cohort (those with normal BMI trajectories) was drawn from the same population as the exposed cohort *	BMI data was self-reported at multiple time points (0 star)	Individuals with a history of any cancer (including renal cell carcinoma) at baseline were excluded from the study *	The study adjusted for multiple confounders, including age, sex, race, smoking status, history of hypertension and diabetes, regular aspirin and ibuprofen use, education level, and others **	Incident cases of renal cell carcinoma (RCC) were ascertained through multiple approaches including self-reports, family reports, death certificates, and medical record abstraction *	Yes * The median follow-up period was 11.5 years	The study does not explicitly state the percentage of participants lost to follow-up, but the large cohort size and detailed reporting suggest minimal loss to follow-up with little risk of bias *	8*

Supplementary Table2. Quality assessment of the included studies using the Newcastle–Ottawa Quality Assessment Scale for case-control studies.

		SELECTION			COMPARABILITY	Exposure			Total scores
**Author, Year**	Is the case definition adequate?<br>a) yes, with independent validation *<br>b) yes, eg record linkage or based on self-reports<br>c) no description	Representativeness of the cases<br>a) consecutive or obviously representative series of cases *<br>b) potential for selection biases or not stated	Selection of Controls<br>a) community controls *<br>b) hospital controls<br>c) no description	Definition of Controls<br>a) no history of disease (endpoint) *<br>b) no description of source	Comparability of cases and controls on the basis of the design or analysis<br>a: study controls for age and/or BMI *<br>b: control for any additional factor*<br>c: no adjustment or matching	Ascertainment of exposure<br>a) secure record (eg surgical records) *<br>b) structured interview where blind to case/control status *<br>c) interview not blinded to case/control status<br>d) written self report or medical record only<br>e) no description	Same method of ascertainment for cases and controls<br>a) yes *<br>b) no	Non-Response rate<br>a) same rate for both groups *<br>b) non respondents described<br>c) rate different and no designation	
Dalmartello M, et al. 2022 [39]	Self-report<br>(0 star)	In Vaud, case recruitment was population-based, with >80% of identified cases interviewed. In Milan, case recruitment was hospital-based. *	Controls were women admitted to the same networks of hospitals as cases, with unrelated diagnoses<br>(0 star)	Women with a history of hysterectomy or related conditions were excluded from controls *	a: study controls for age *<br>b: study controls for additional factors (education, diabetes, family history of EC, age at menarche, menopausal status, parity, ever oral contraceptive use, ever HRT use, and smoking) *	Centrally trained interviewers administered the same structured questionnaire to cases and controls<br>(0 star)	The same questionnaire was used for both cases and controls. *	Less than 5% of patients refused to participate, but specific rates for cases and controls were not provided<br>(0 star)	5*
Lavalette C, et al. 2022 [108]	Case were incident prostate cancer cases newly diagnosed in 2012-2013, validated by the Hérault Cancer Registry. *	Cases were all men newly diagnosed with prostate cancer in 2012-2013, aged under 75 and living in the département of Hérault. *	Controls were randomly selected from the general population of the same département *	Controls had no history of prostate cancer at the time of inclusion. *	a: study controls for age *<br>b: study controls for additional factors (family history of prostate cancer, ethnic origin) *	Exposure information was collected using a face-to-face standardized computerized questionnaire.<br>(0 star)	The same questionnaire was used for both cases and controls. *	Participation rates were similar for cases (75%) and controls (79%). *	8*
Vallières E, et al. 2021 [207]	Cases were histologically confirmed incident prostate cancer cases diagnosed between 2005-2009 *	Cases were all eligible men diagnosed with prostate cancer during the study period in participating hospitals. *	Controls were randomly selected from electoral lists of the same residential areas as cases. *	Controls had no history of prostate cancer. *	a: study controls for age *<br>b: study controls for additional factors (ancestry, first-degree family history of prostate cancer, education, income) *	In-person interviews were conducted to collect information on anthropometric measures.<br>(0 star)	The same questionnaire was used for both cases and controls. *	Response rates were 79.4% for cases and 55.5% for controls. Reasons for non-participation were provided.<br>(0 star)	7*

Supplementary Table 3. Results of Fixed/Random effect meta-analysis to estimate pooled unadjusted ES (95%CI) for various cancer outcomes by trajectories

Cancer Outcome	Trajectories	[@]N	[#]Pooled Crude OR (95%CI)	Heterogeneity I^2%	Harbord's Publication bias test
Breast	Normal to obesity	3	1.050 (0.979, 1.127)	63.5%	0.354
	Normal to overweight	5	1.007 (0.960, 1.057)	57.1%	0.364
	Overweight to obesity	3	0.917 (0.792, 1.063)	49.7%	0.522
Colorectal	Normal to obesity	4	**1.304 (1.123, 1.515)**	75.8%	0.627
	Normal to overweight	6	**1.215 (1.142, 1.292)**	31.2%	0.208
	Overweight to obesity	5	**1.490 (1.130, 1.965)**	81.3%	0.599
Gastrointestinal	Normal to obesity	2	**2.227 (1.842, 2.694)**	72.7%	--
	Normal to overweight	2	**1.787 (1.545, 2.068)**	0.0%	--
	Overweight to obesity	2	**2.107 (1.428, 3.108)**	0.0%	--
Kidney	Normal to obesity	1	**2.440 (1.891, 3.148)**	--	--
	Normal to overweight	1	**1.696 (1.362, 2.113)**	--	--
	Overweight to obesity	1	**2.711 (1.792, 4.102)**	--	--
Liver	Normal to obesity	1	**2.756 (1.738, 4.370)**	--	--
	Normal to overweight	1	**1.595 (1.039, 2.448)**	--	--
	Overweight to obesity	1	1.801 (0.821, 3.952)	--	--
Lung	Normal to obesity	3	**0.592 (0.534, 0.656)**	0.0%	0.384
	Normal to overweight	3	**0.821 (0.774, 0.871)**	0.0%	0.673
	Overweight to obesity	3	**0.630 (0.544, 0.731)**	72.8%	0.757
Endometrial	Normal to obesity	0	--	--	--
	Normal to overweight	1	1.255 (0.972, 1.620)	--	--
	Overweight to obesity	1	**1.881(1.475, 2.399)**	--	--
Prostate	Normal to obesity	8	**0.760 (0.642, 0.900)**	56.7%	0.126
	Normal to overweight	5	0.980 (0.887, 1.082)	0.0%	**0.378**
	Overweight to obesity	9	**0.740 (0.634, 0.865)**	21.3%	0.993
Pancreatic	Normal to obesity	5	1.387 (0.790, 2.434)	96.4%	0.627
	Normal to overweight	5	1.147 (0.761, 1.727)	96.4%	0.874
	Overweight to obesity	5	0.964 (0.810, 1.148)	30.1%	0.683
Obesity-related	Normal to obesity	17	**1.458 (1.247, 1.705)**	93.2%	0.200
	Normal to overweight	23	**1.223 (1.121, 1.334)**	89.3%	0.806
	Overweight to obesity	20	**1.368 (1.202, 1.556)**	74.2%	0.695
Women-related	Normal to obesity	3	1.120 (0.945,1.327)	63.5%	0.354
	Normal to overweight	6	0.994 (0.875, 1.129)	58.5%	0.671
	Overweight to obesity	4	1.186 (0.766, 1.834)	89.3%	0.664
Overall cancers	Normal to obesity	36	**1.165 (1.020, 1.329)**	94.6%	0.261
	Normal to overweight	53	**1.088 (1.009, 1.174)**	91.9%	0.485
	Overweight to obesity	46	1.149 (0.997, 1.325)	90.1%	0.112

[#]Fixed/Random effect Mantel-Haenszel method, ref: Normal to Normal trajectory group, [@] Number of records to get pooled

Supplementary Table 4. Results of Random effect sub-group analysis to estimate pooled unadjusted ES (95%CI) for various cancer outcomes by trajectories based on follow-up duration (<15 years vs. ≥15 years), BMI assessment method (measured vs. recalled), and geographic region

	**time of follow-up less than 15**						**time of follow-up over than 15**				
Outcome	Trajectories	[@]N	[&]Heterogeneity I^2%	Pooled adjusted ES (95%CI)*			[@]N	[&]Heterogeneity I^2%	Pooled adjusted ES (95%CI)*		
Women-related Cancer	Stable Normal	Ref.	Ref.	Ref.			Ref.	Ref.	Ref.		
	Normal to obesity	8	89.4%	1.132	0.853	1.412	1	--	1.200	0.900	1.500
	Normal to overweight	4	70.5%	1.061	0.953	1.170	4	64.2%	1.008	0.609	1.407
	Overweight to obesity	15	84.4%	1.117	0.822	1.411	1	--	**2.030**	**1.120**	**2.940**
Obesity-related Cancer	Stable Normal	Ref.	Ref.	Ref.			Ref.	Ref.	Ref.		
	Normal to obesity	30	63.4%	**1.277**	**1.136**	**1.418**	6	12.5%	**1.368**	**1.267**	**1.469**
	Normal to overweight	19	0.0%	**1.072**	**1.034**	**1.111**	7	0.0%	**1.148**	**1.091**	**1.204**
	Overweight to obesity	62	0.0%	**1.300**	**1.215**	**1.385**	6	16.6%	**1.434**	**1.222**	**1.646**
Overall cancers	Stable Normal	Ref.	Ref.	Ref.			Ref.	Ref.	Ref.		
	Normal to obesity	10	51.6%	**1.299**	**1.152**	**1.447**	20	86.6%	**1.190**	**1.074**	**1.306**
	Normal to overweight	14	47.5%	**1.107**	**1.017**	**1.196**	48	54.9%	**1.051**	**1.012**	**1.090**
	Overweight to obesity	9	9.5%	**1.437**	**1.241**	**1.633**	28	77.6%	**1.161**	**1.046**	**1.276**
	**recalled BMI**						**% combined measured BMI**				
Women-related Cancer	Stable Normal	Ref.	Ref.	Ref.			Ref.	Ref.	Ref.		
	Normal to obesity	1	38.9%	1.030	0.950	1.110	11	74.2%	1.033	0.898	1.168
	Normal to overweight	3	83.3%	1.104	0.840	1.368	4	68.0%	1.224	0.954	1.493
	Overweight to obesity	2	57.1%	1.364	0.251	2.477	5	72.9%	1.256	0.894	1.619
Obesity-related Cancer	Stable Normal	Ref.	Ref.	Ref.			Ref.	Ref.	Ref.		
	Normal to obesity	12	74.1%	**1.638**	**1.317**	**1.959**	13	47.7%	**1.320**	**1.234**	**1.406**
	Normal to overweight	12	49.8%	**1.234**	**1.102**	**1.366**	34	0.0%	**1.102**	**1.069**	**1.135**
	Overweight to obesity	15	48.6%	**1.695**	**1.286**	**2.103**	20	9.9%	**1.298**	**1.213**	**1.382**
Overall cancers	Stable Normal	Ref.	Ref.	Ref.			Ref.	Ref.	Ref.		
	Normal to obesity	23	89.5%	1.162	0.979	1.346	22	87.5%	**1.250**	**1.135**	**1.365**
	Normal to overweight	26	80.9%	1.076	0.985	1.167	59	59.7%	**1.065**	**1.026**	**1.104**
	Overweight to obesity	28	76.1%	1.067	0.861	1.274	34	76.5%	**1.230**	**1.124**	**1.335**
	**America**						**Europe**				
Women-related Cancer	Stable Normal	Ref.	Ref.	Ref.			Ref.	Ref.	Ref.		
	Normal to obesity	7	72.7%	1.069	0.917	1.221	2	13.2%	1.051	0.941	1.161
	Normal to overweight	2	88.6%	1.184	0.714	1.653	2	83.3%	1.364	0.251	2.477
	Overweight to obesity	3	86.0%	1.264	0.791	1.737	5	68.8%	0.982	0.736	1.228
Obesity-related Cancer	Stable Normal	Ref.	Ref.	Ref.			Ref.	Ref.	Ref.		
	Normal to obesity	39	0.0%	**1.121**	**1.086**	**1.157**	--	--	--		
	Normal to overweight	21	62.1%	**1.395**	**1.273**	**1.517**	--	--	--		
	Overweight to obesity	28	30.1%	**1.375**	**1.245**	**1.504**	--	--	--		
Overall cancers	Stable Normal	Ref.	Ref.	Ref.			Ref.	Ref.	Ref.		
	Normal to obesity	60	58.2%	**1.078**	**1.038**	**1.119**	9	39.9%	0.981	0.844	1.118
	Normal to overweight	40	82.2%	**1.185**	**1.059**	**1.312**	6	0.0%	1.031	0.956	1.106
	Overweight to obesity	30	85.9%	**1.307**	**1.183**	**1.430**	6	27.7%	1.019	0.710	1.328
	**Asia**						**Australia**				
Women-related Cancer	Stable Normal	Ref.	Ref.	Ref.			Ref.	Ref.	Ref.		
	Normal to obesity	--	--	--			--	--	--		
	Normal to overweight	--	--	--			--	--	--		
	Overweight to obesity	--	--	--			--	--	--		
Obesity-related Cancer	Stable Normal	Ref.	Ref.	Ref.			Ref.	Ref.	Ref.		
	Normal to obesity	--	--	--			--	--	--		
	Normal to overweight	--	--	--			--	--	--		
	Overweight to obesity	--	--	--			--	--	--		
Overall cancers	Stable Normal	Ref.	Ref.	Ref.			Ref.	Ref.	Ref.		
	Normal to obesity	--	--	--			--	--	--		
	Normal to overweight	--	--	--			--	--	--		
	Overweight to obesity	--	--	--			--	--	--		

#random effect Mantel-Haenszel method, @ Number of records to get pooled
& I^2%, chi^2 Test
$Harbord publication bias test for odds ratio
-- Insufficient data
*Bold values indicate significant results

Supplementary Figure 1. Forest plots for the pooled effect size (ES) of composite outcome of obesity related cancers for different BMI trajectories compared to stable normal weight trajectory: (A) ES of composite outcome of obesity related for normal to obesity trajectory; (B) ES of composite outcome of obesity related for normal to overweight trajectory (C) ES of composite outcome of obesity related for overweight to obesity trajectory

A:

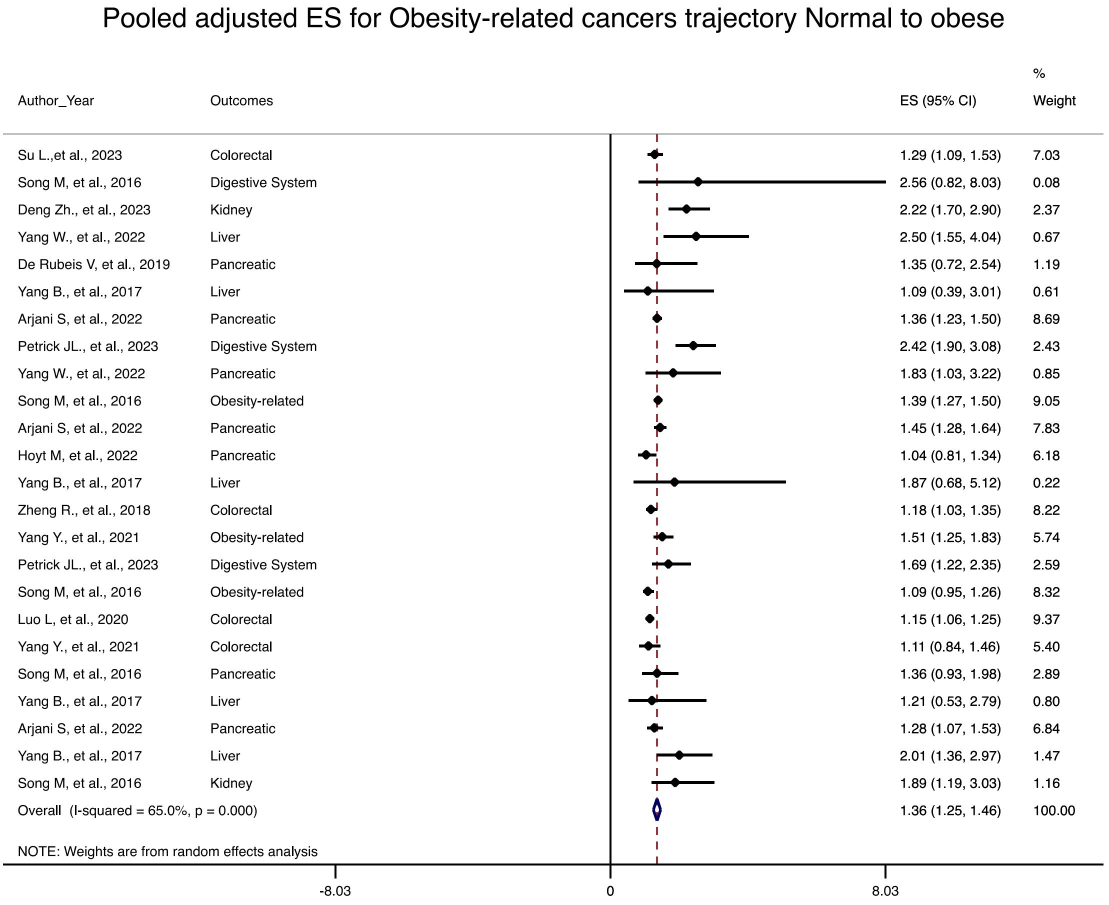

B:

Pooled adjusted ES for Obesity-related cancers trajectory Normal to overweight

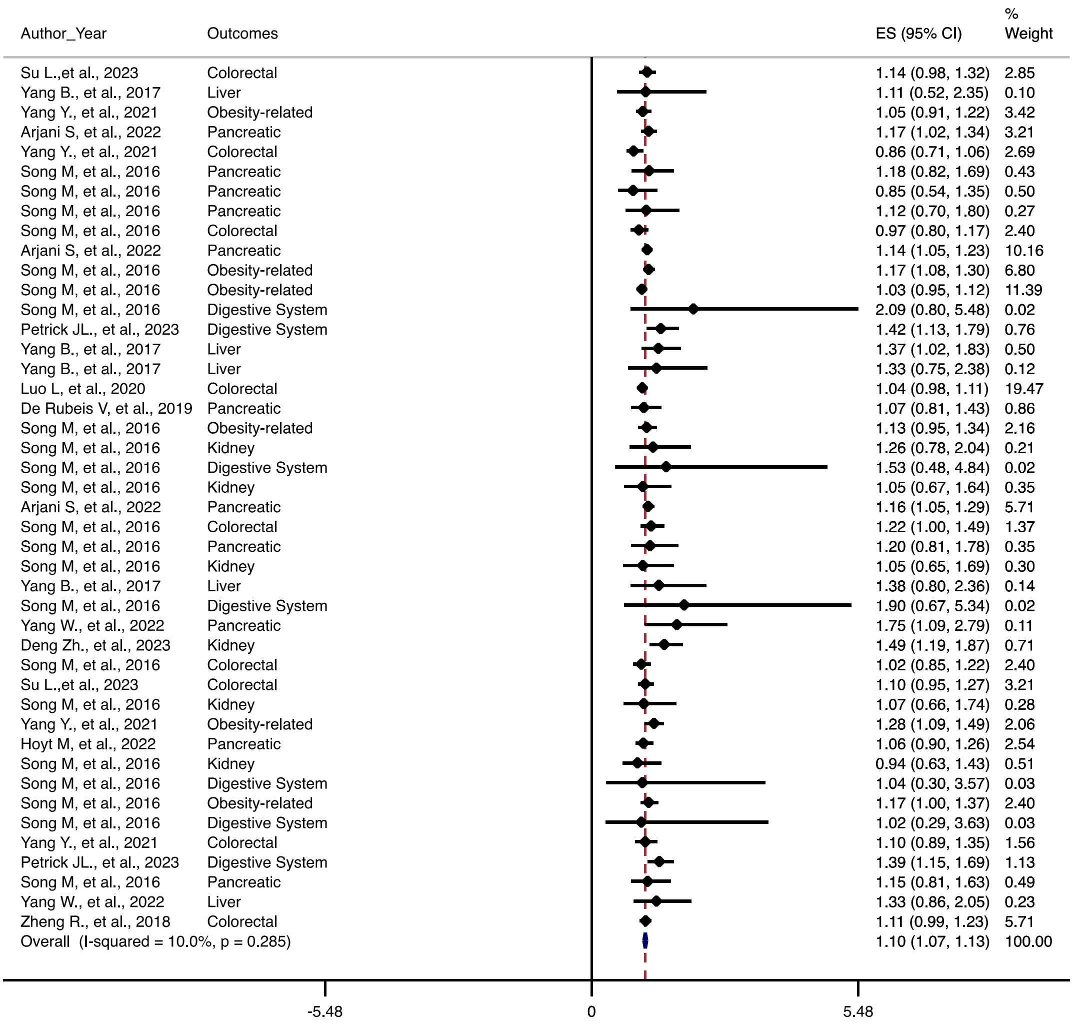

C:

Pooled adjusted ES for Obesity-related cancers trajectory Overweight to obese

Author_Year	Outcomes	ES (95% CI)	% Weight
Yang W., et al., 2022	Pancreatic	4.26 (2.16, 8.41)	0.05
Song M, et al., 2016	Colorectal	1.40 (1.13, 1.74)	5.47
Song M, et al., 2016	Obesity-related	1.17 (0.97, 1.42)	10.05
Yang B., et al., 2017	Liver	1.93 (0.55, 6.72)	0.05
Song M, et al., 2016	Digestive System	2.19 (0.63, 7.70)	0.04
Yang B., et al., 2017	Liver	1.58 (0.58, 4.33)	0.14
De Rubeis V, et al., 2019	Pancreatic	1.55 (1.04, 2.32)	1.24
Luo L, et al., 2020	Colorectal	1.33 (1.11, 1.61)	8.14
Arjani S, et al., 2022	Pancreatic	1.22 (0.85, 1.74)	2.57
Song M, et al., 2016	Kidney	0.93 (0.53, 1.64)	1.65
Su L.,et al., 2023	Colorectal	1.37 (1.13, 1.68)	6.73
Yang B., et al., 2017	Liver	1.82 (0.94, 3.52)	0.31
Zheng R., et al., 2018	Colorectal	1.27 (0.99, 1.64)	4.82
Petrick JL., et al., 2023	Digestive System	4.07 (2.32, 7.15)	0.09
Song M, et al., 2016	Digestive System	3.01 (1.04, 9.13)	0.03
Yang Y., et al., 2021	Colorectal	1.47 (0.98, 2.20)	1.37
Hoyt M, et al., 2022	Pancreatic	1.43 (0.87, 2.34)	0.94
Yang Y., et al., 2021	Obesity-related	1.05 (0.74, 1.50)	3.52
Yang W., et al., 2022	Liver	1.99 (0.89, 4.44)	0.16
Yang B., et al., 2017	Liver	1.25 (0.15, 10.10)	0.02
De Rubeis V, et al., 2019	Pancreatic	0.36 (0.08, 1.53)	0.97
Yang B., et al., 2017	Liver	1.90 (1.22, 2.95)	0.68
Song M, et al., 2016	Obesity-related	1.28 (1.17, 1.41)	35.34
Yang Y., et al., 2021	Obesity-related	1.53 (1.16, 2.01)	2.82
Arjani S, et al., 2022	Pancreatic	1.76 (1.30, 2.38)	1.75
Petrick JL., et al., 2023	Digestive System	2.90 (1.67, 5.04)	0.18
Song M, et al., 2016	Pancreatic	1.50 (0.92, 2.46)	0.86
Arjani S, et al., 2022	Pancreatic	1.43 (1.14, 1.80)	4.67
Song M, et al., 2016	Kidney	1.92 (1.15, 3.21)	0.48
Yang Y., et al., 2021	Colorectal	0.92 (0.60, 1.41)	3.10
Yang B., et al., 2017	Liver	4.09 (1.32, 12.70)	0.02
Song M, et al., 2016	Pancreatic	1.39 (0.91, 2.12)	1.39
Deng Zh., et al., 2023	Kidney	2.78 (1.81, 4.27)	0.34
Overall (I-squared = 29.5%, p = 0.059)		1.31 (1.24, 1.38)	100.00

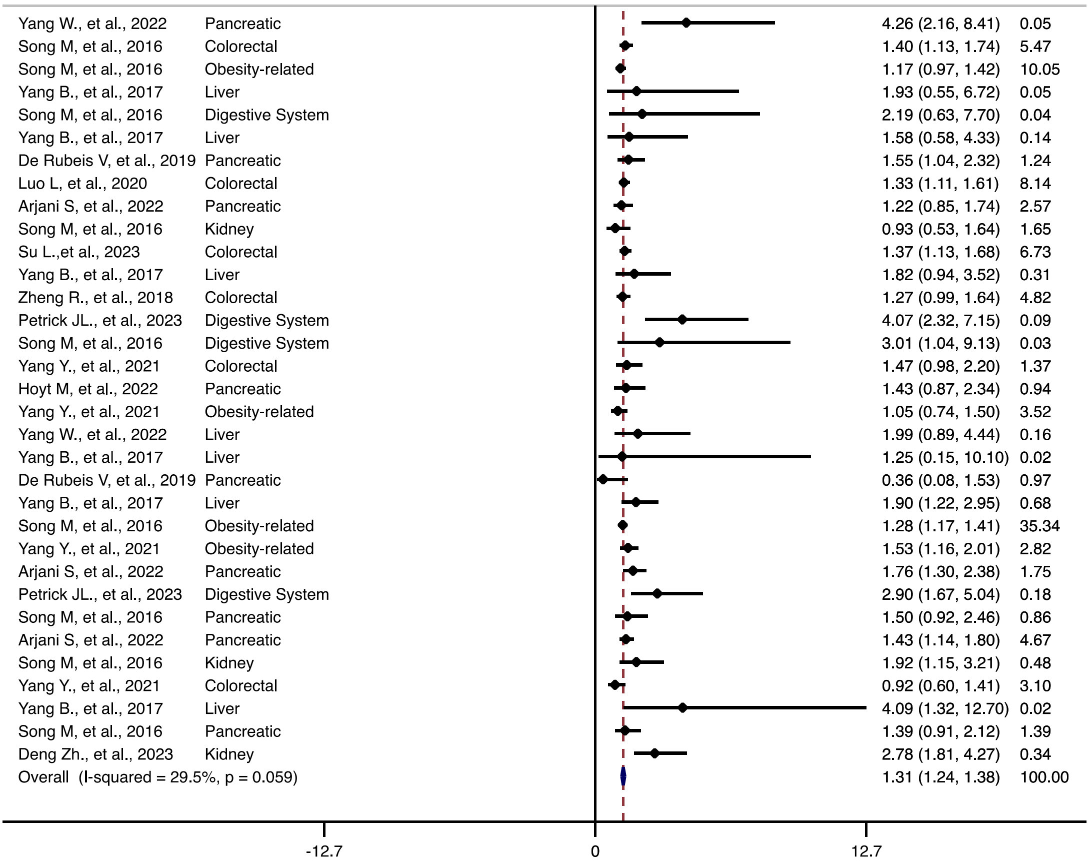

Supplementary Figure 2. Forest plots for the pooled effect size (ES) of composite outcome of gastrointestinal cancers for different BMI trajectories compared to stable normal weight trajectory

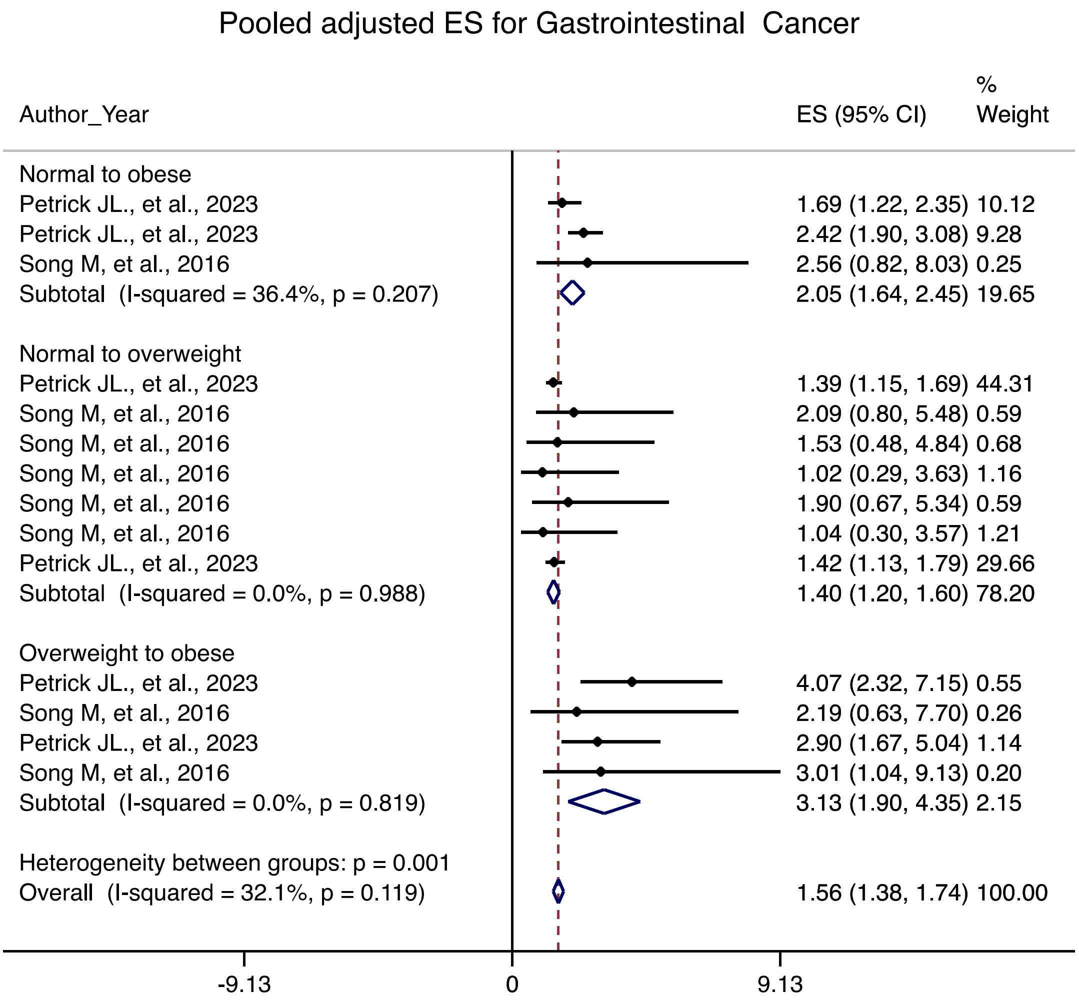

Supplementary Figure 3. Forest plots for the pooled effect size (ES) of composite outcome women related cancers for different BMI trajectories compared to stable normal weight trajectory

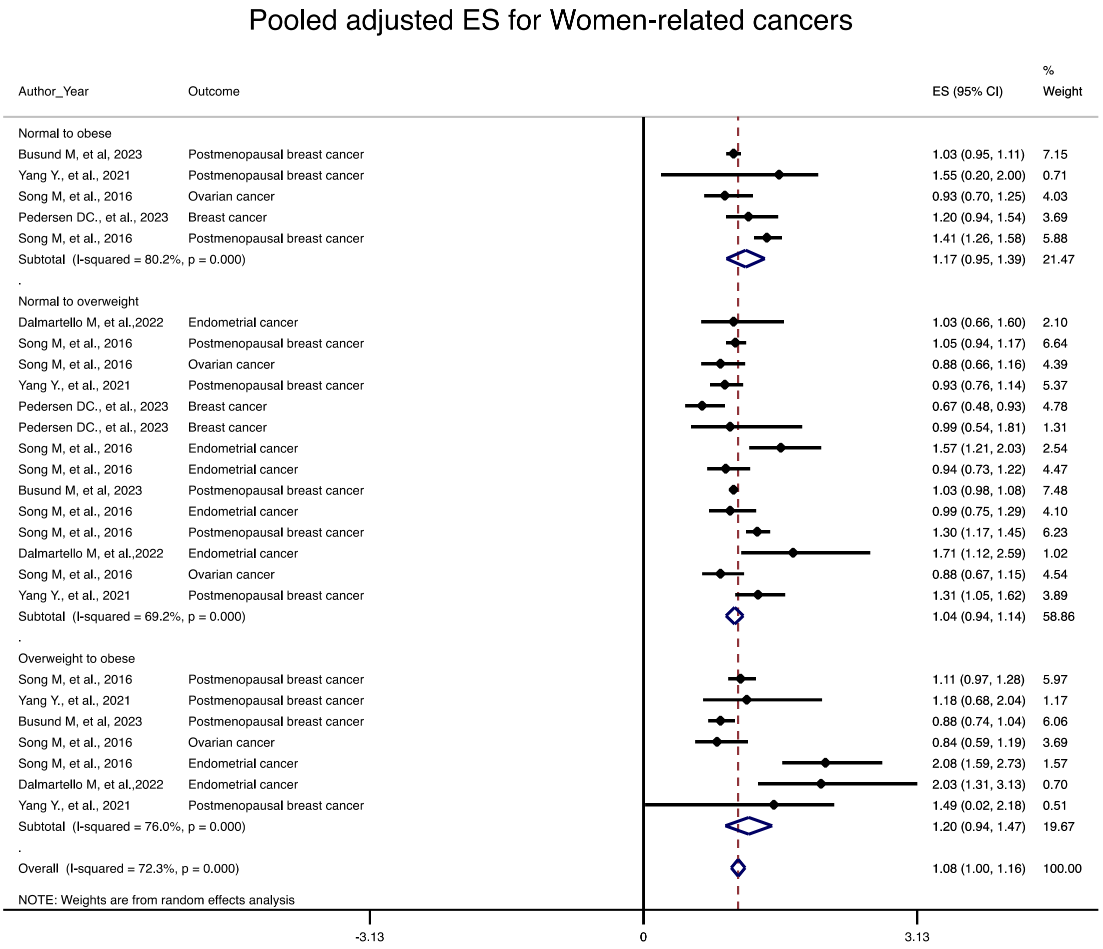

Supplementary Figure 4. Forest plots for the pooled effect size (ES) of colorectal cancer for different BMI trajectories compared to stable normal weight trajectory

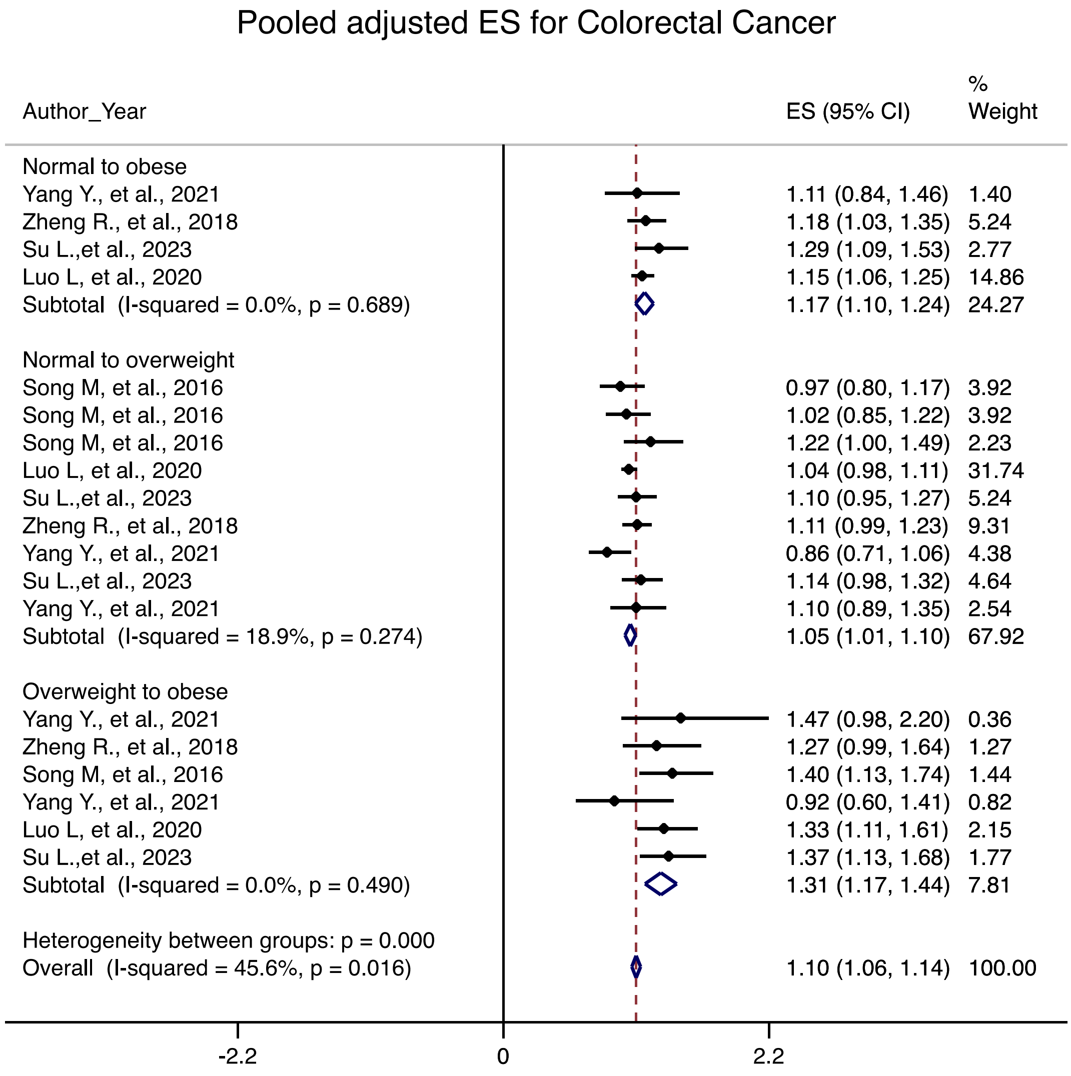

Supplementary Figure 5. Forest plots for the pooled effect size (ES) of pancreatic cancer for different BMI trajectories compared to stable normal weight trajectory

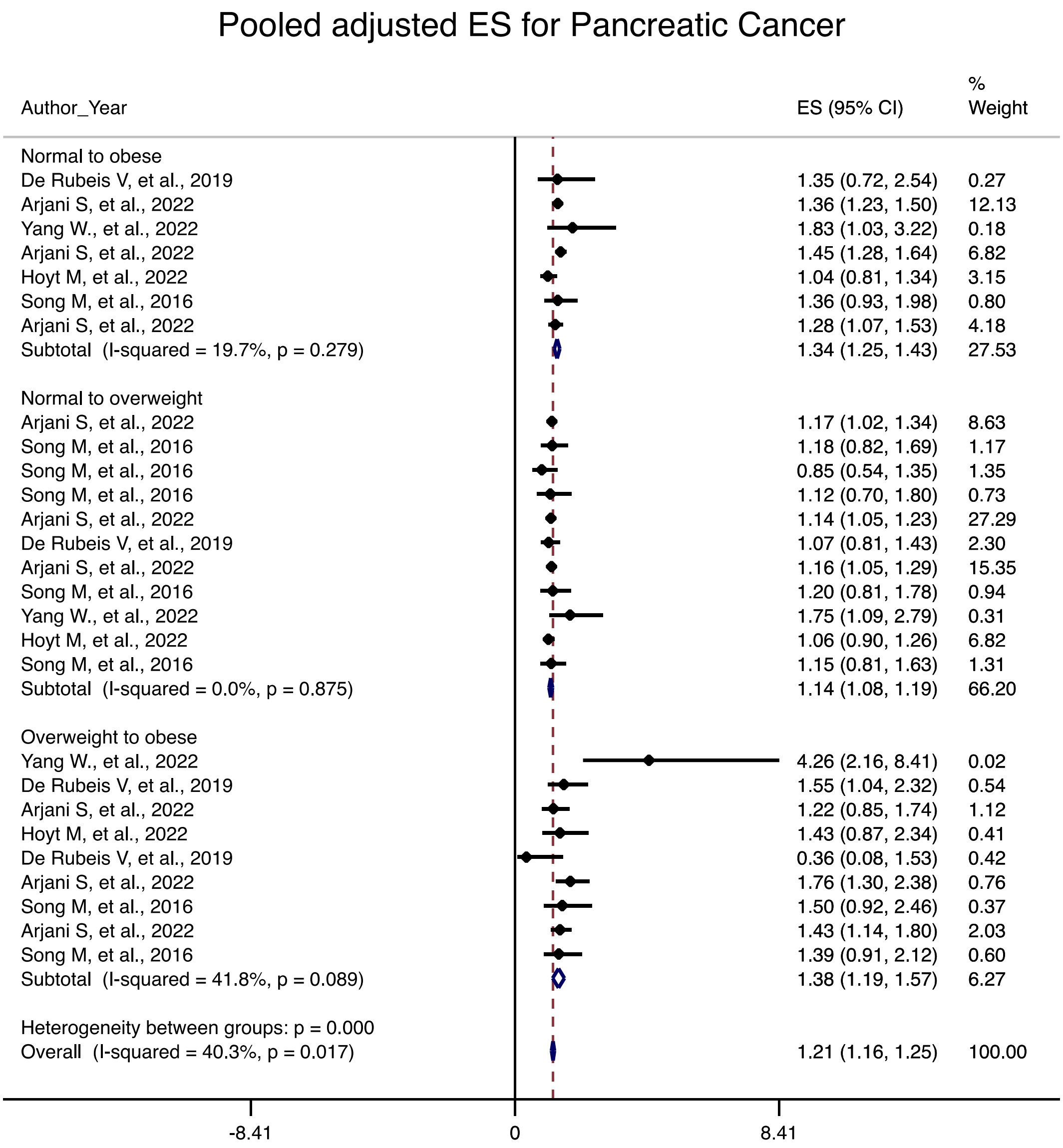

Supplementary Figure 6. Forest plots for the pooled effect size (ES) of liver cancer for different BMI trajectories compared to stable normal weight trajectory

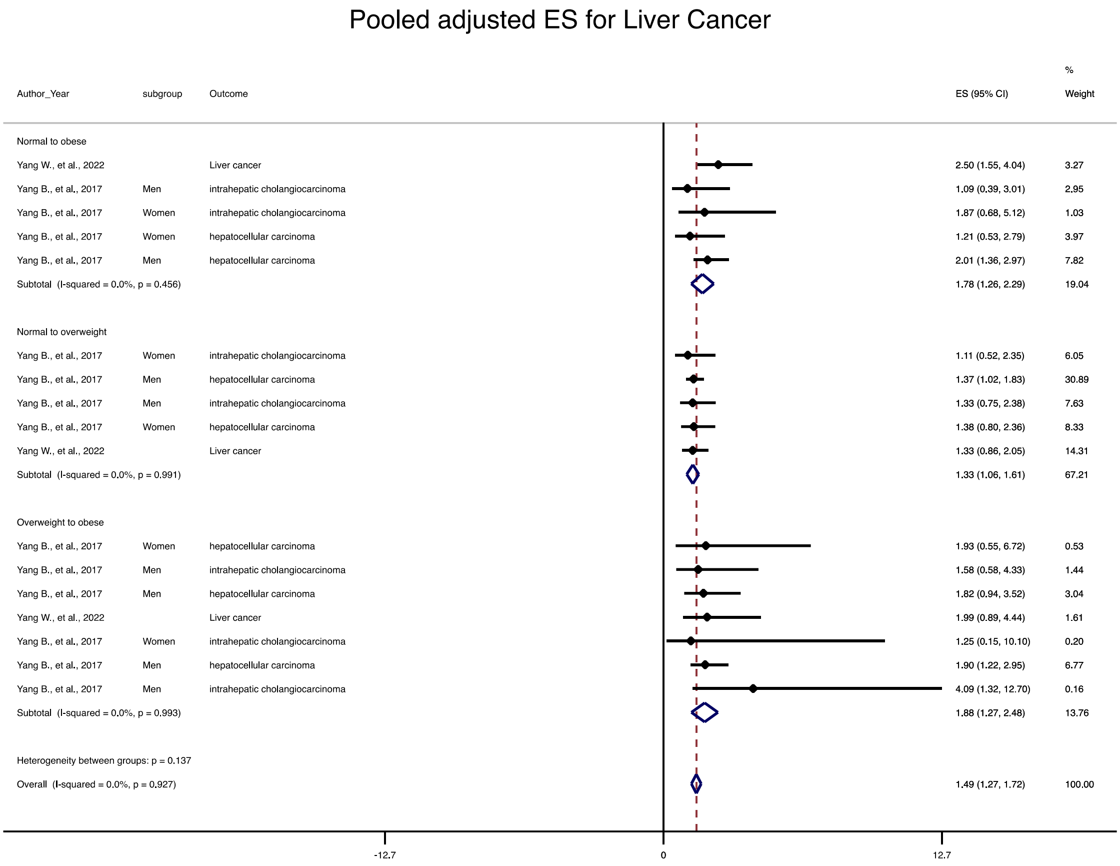

Supplementary Figure 7. Forest plots for the pooled effect size (ES) of kidney cancer for different BMI trajectories compared to stable normal weight trajectory

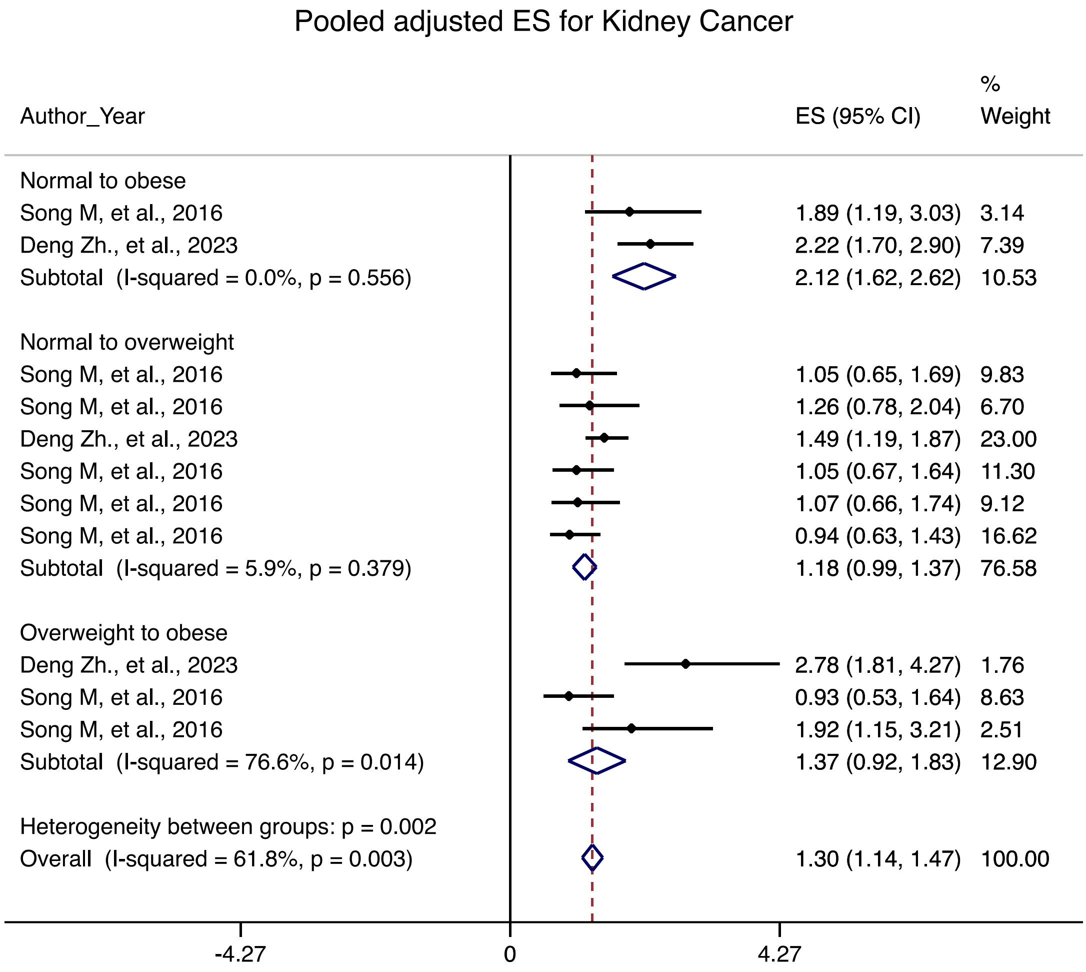

Supplementary Figure 8. Forest plots for the pooled effect size (ES) of endometrial cancer for different BMI trajectories compared to stable normal weight trajectory

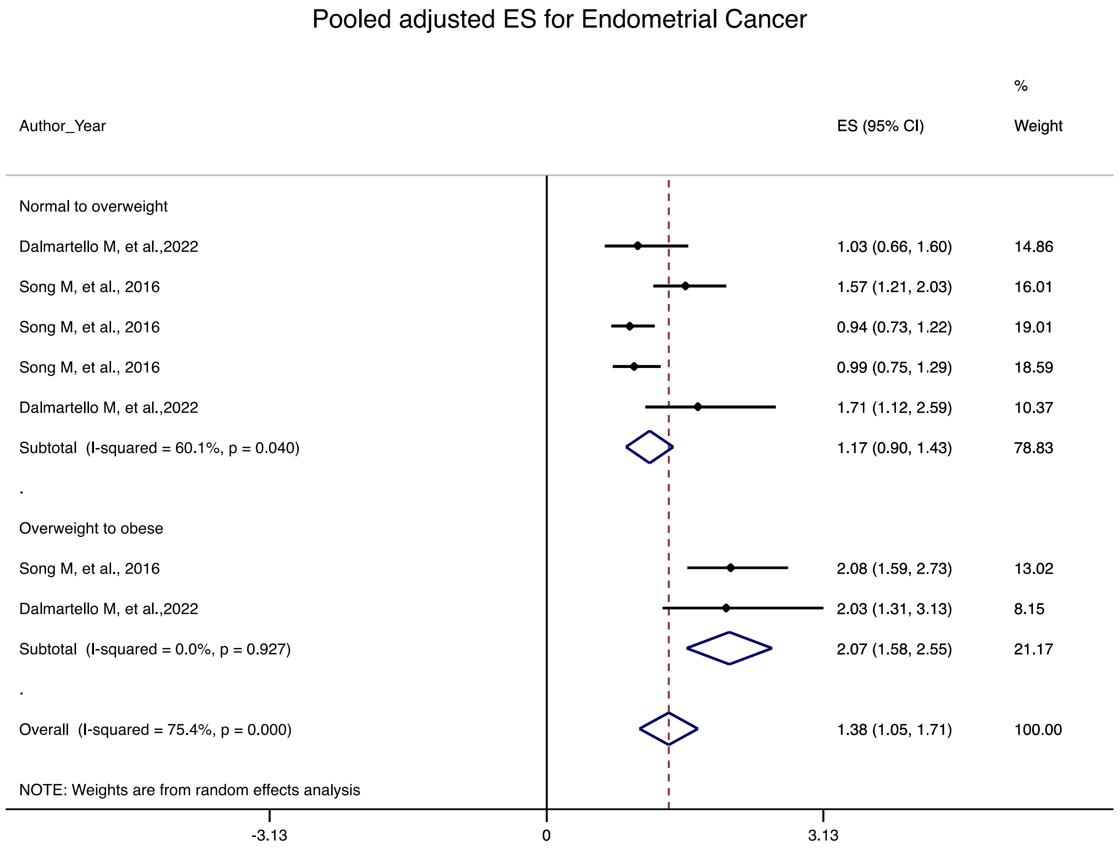

Supplementary Figure 9. Forest plots for the pooled effect size (ES) of prostate cancer for different BMI trajectories compared to stable normal weight trajectory

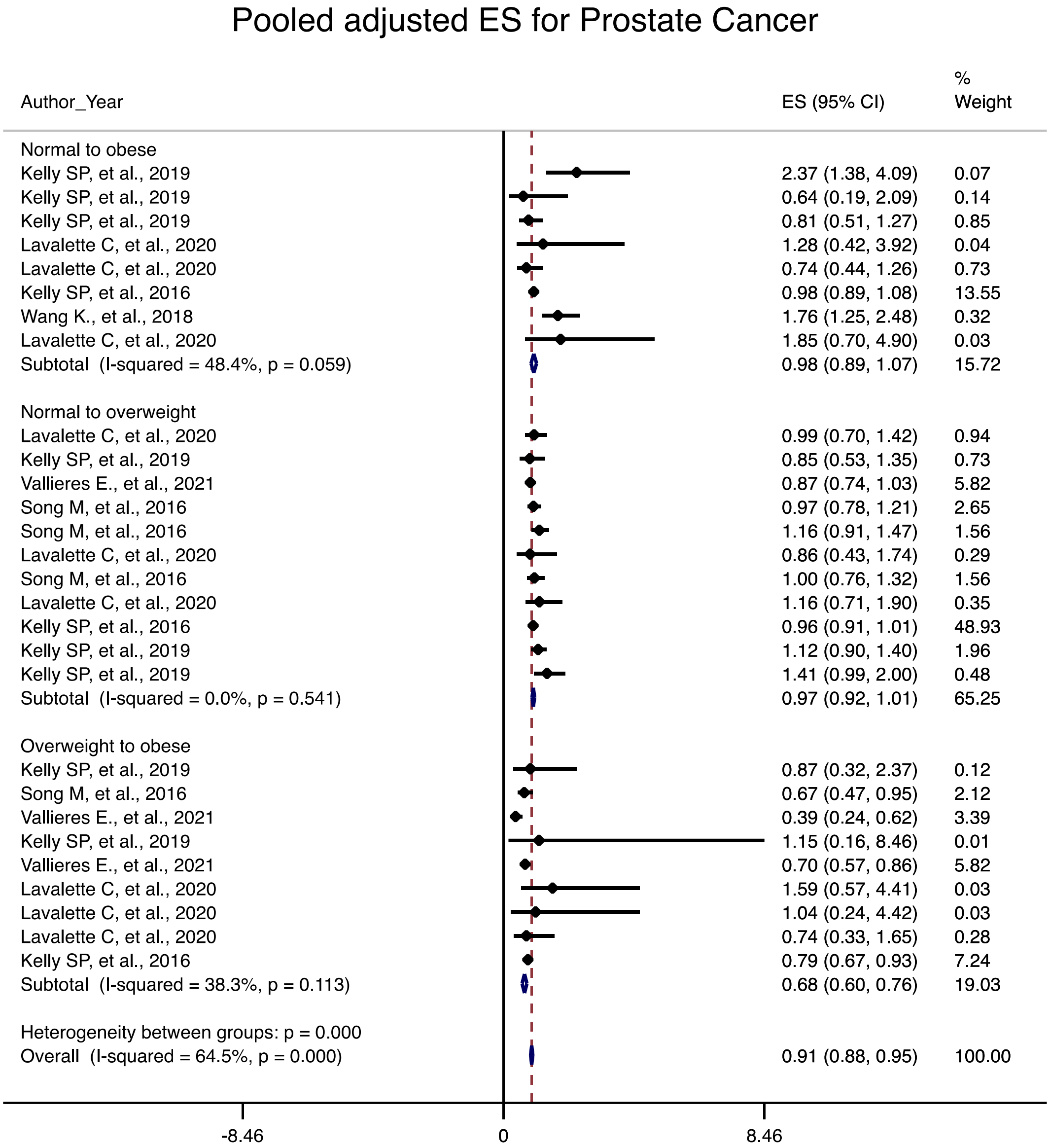

Supplementary Figure 10. Forest plots for the pooled effect size (ES) of lung cancer for different BMI trajectories compared to stable normal weight trajectory

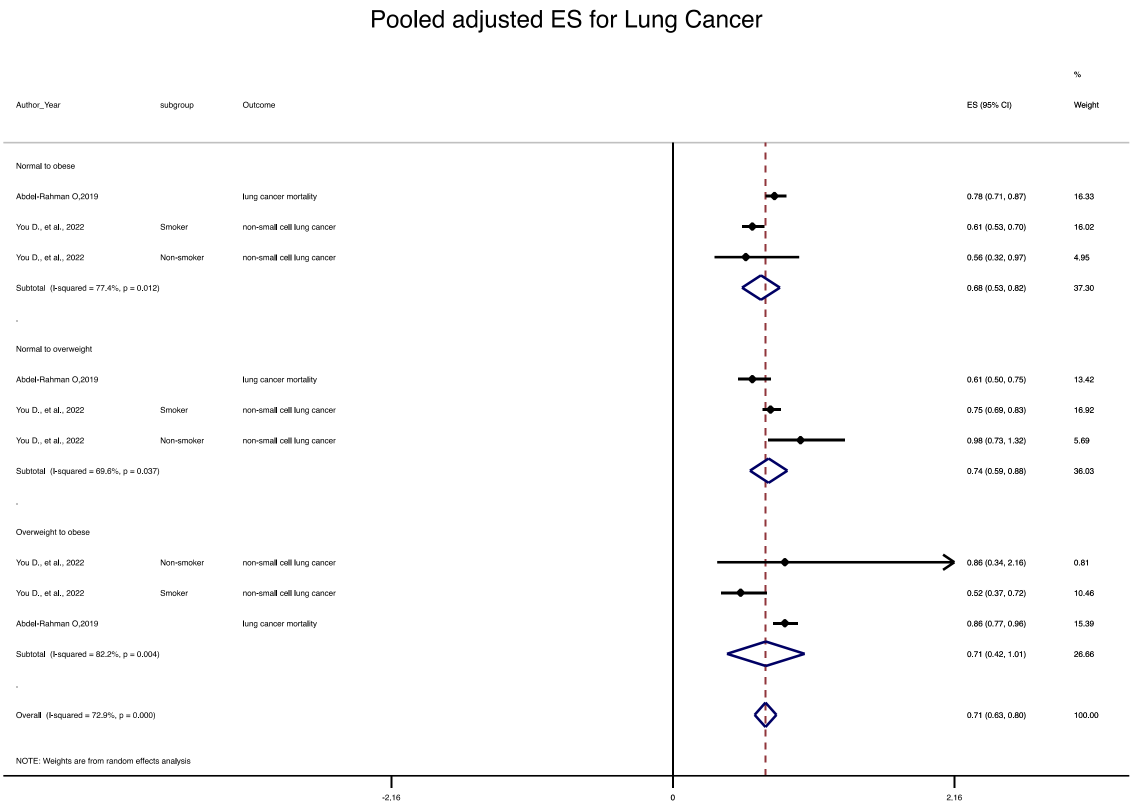

Supplementary Figure 11. Funnel plot assessing publication bias in the association between BMI trajectories and obesity related cancer and overall cancers risks

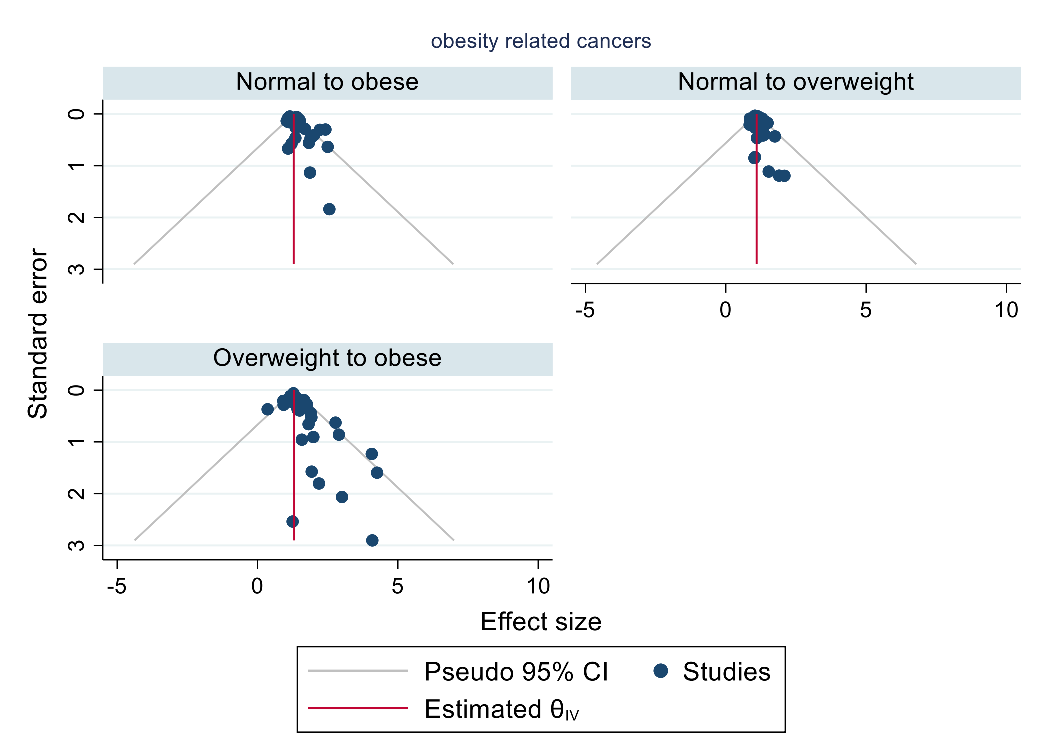

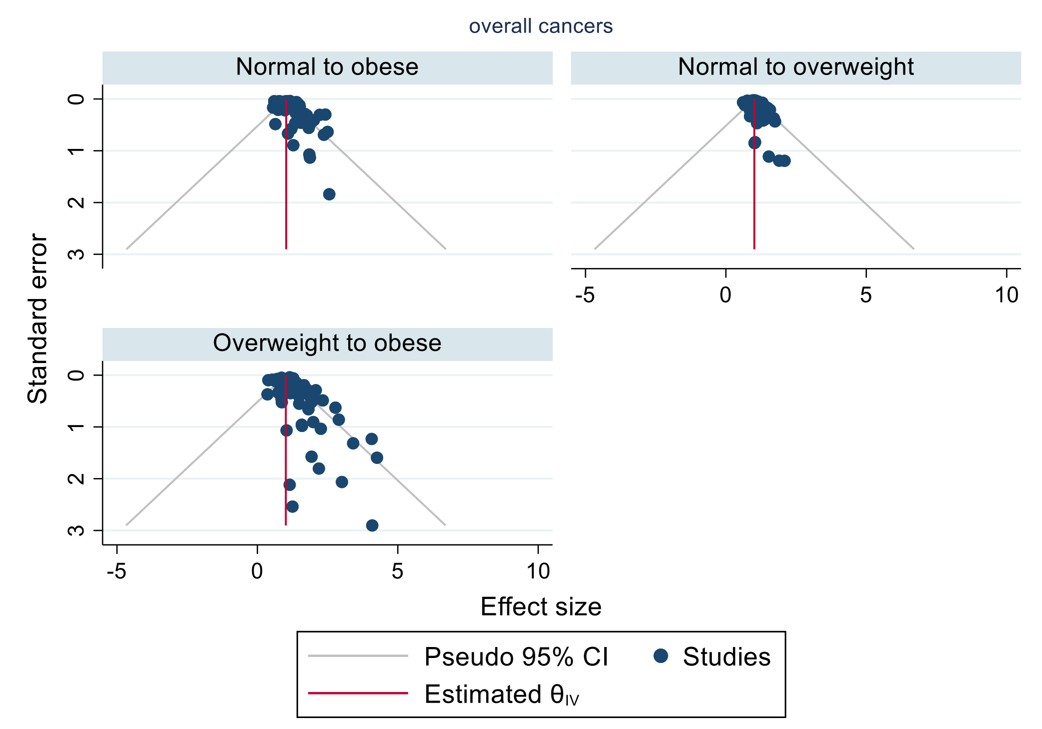

Supplementary Figure 12. Forest plots for the pooled effect size (ES) of subgroup analysis for different BMI trajectories

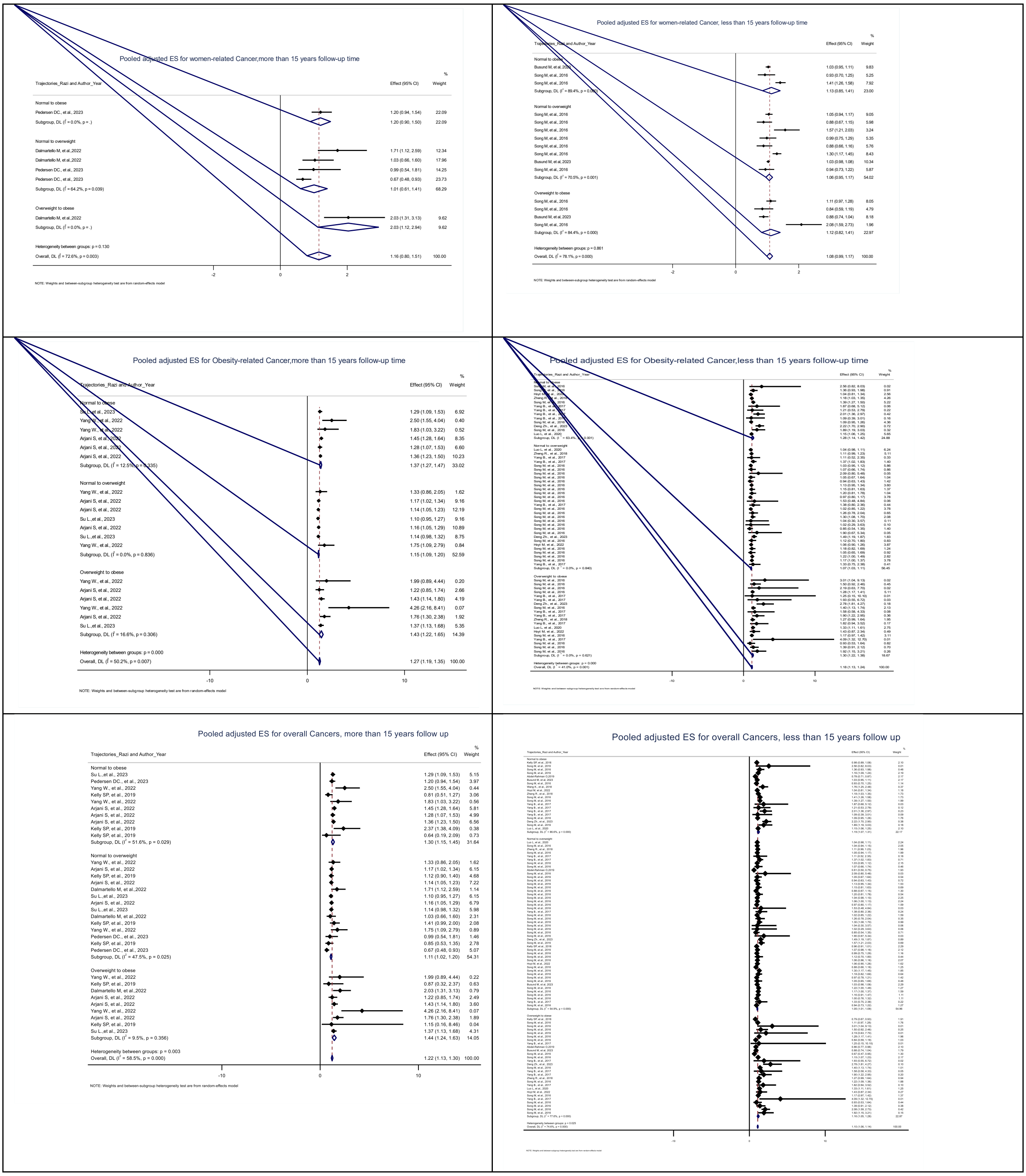

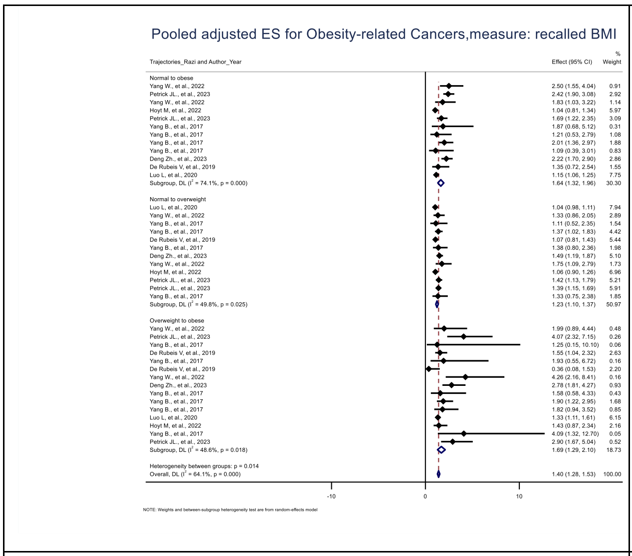
Pooled adjusted ES for Obesity-related Cancers,measure: recalled BMI

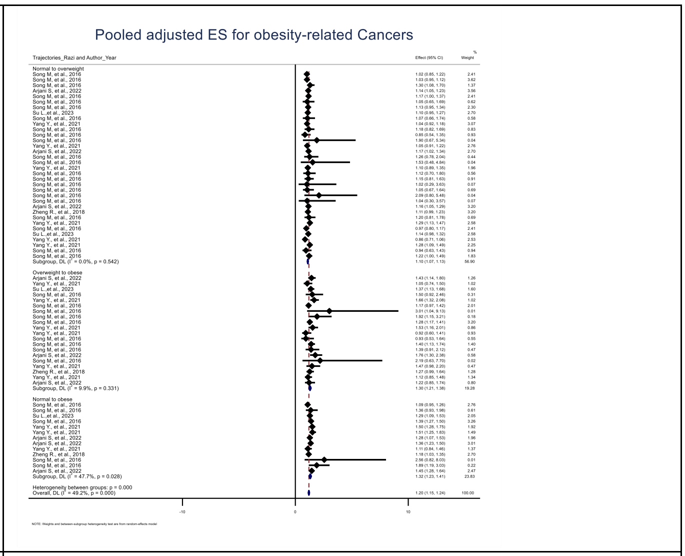
Pooled adjusted ES for obesity-related Cancers

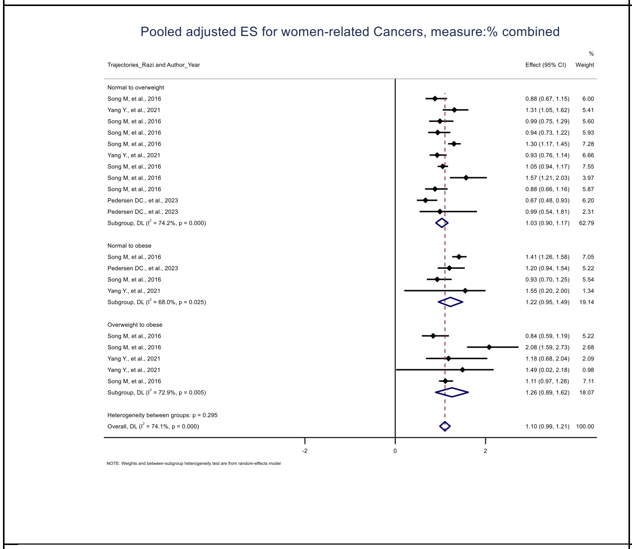
Pooled adjusted ES for women-related Cancers, measure:% combined

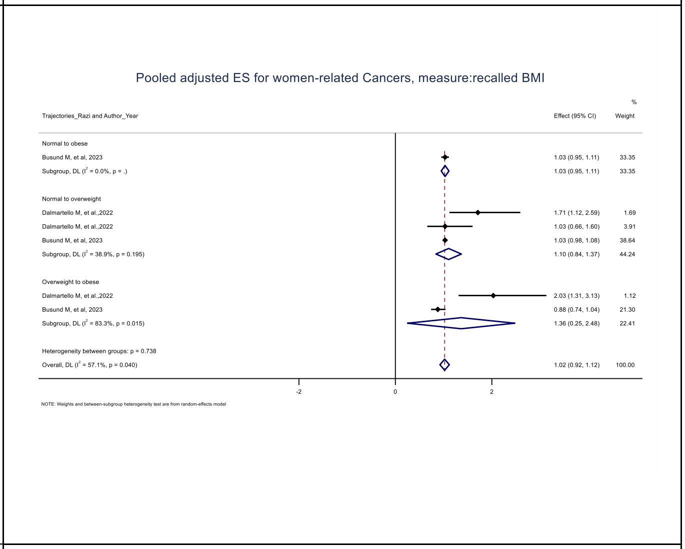
Pooled adjusted ES for women-related Cancers, measure:recalled BMI

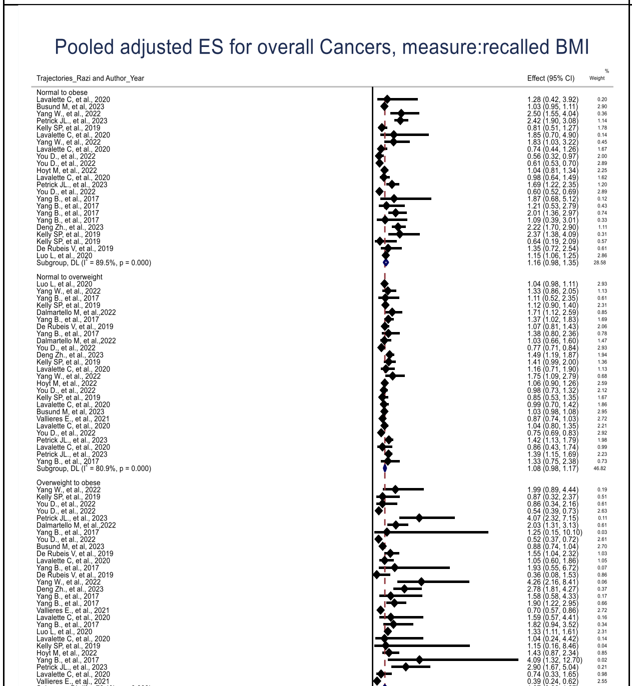
Pooled adjusted ES for overall Cancers, measure:recalled BMI

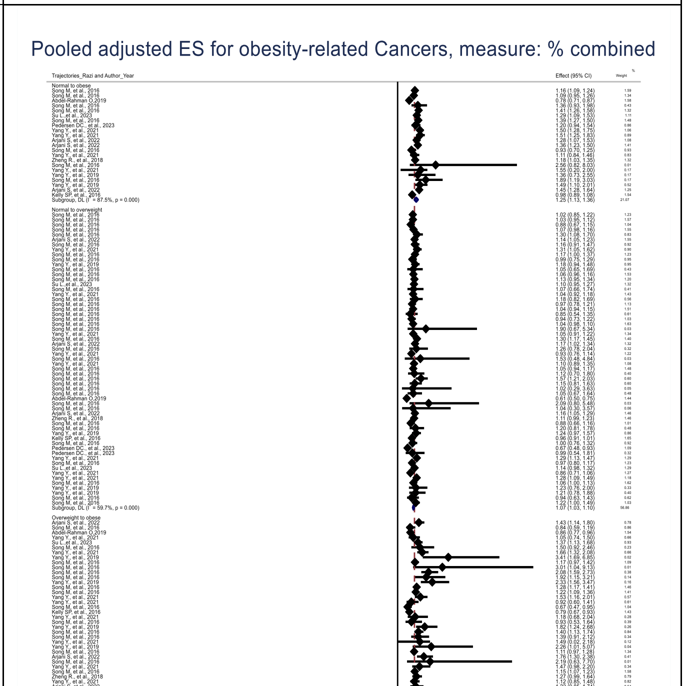
Pooled adjusted ES for obesity-related Cancers, measure: % combined

Pooled adjusted ES for women-related Cancers in America

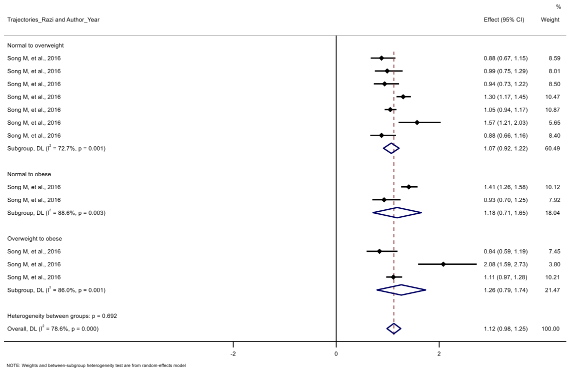

Pooled adjusted ES for women-related Cancers in Europe

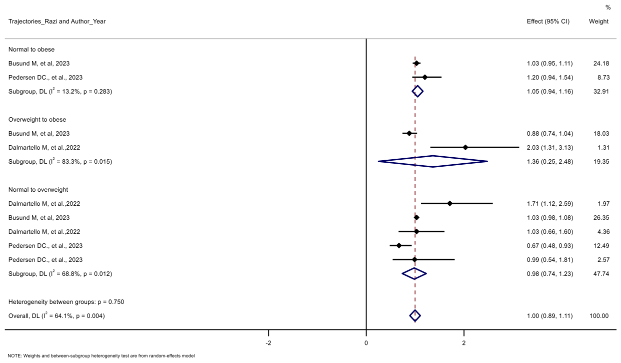

Pooled adjusted ES for obesity-related Cancers in America

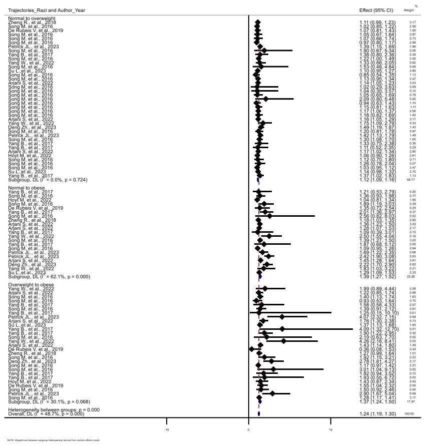

Pooled adjusted ES for overall Cancers in America

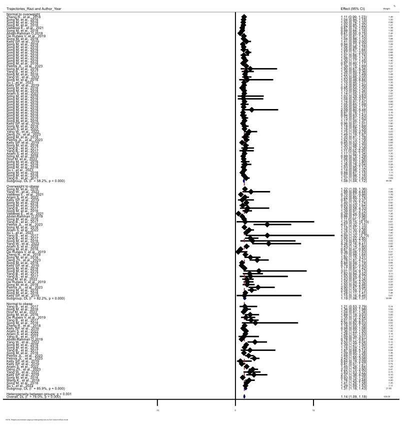

Pooled adjusted ES for obesity-related Cancers in europe

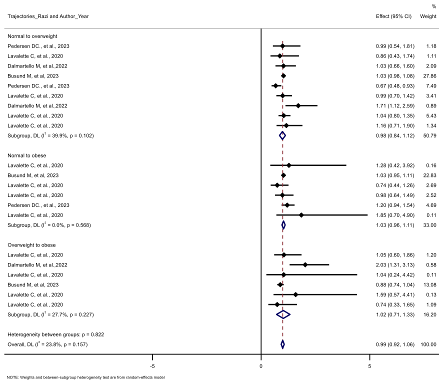

Supplementary Figure 13. Sensitivity analysis

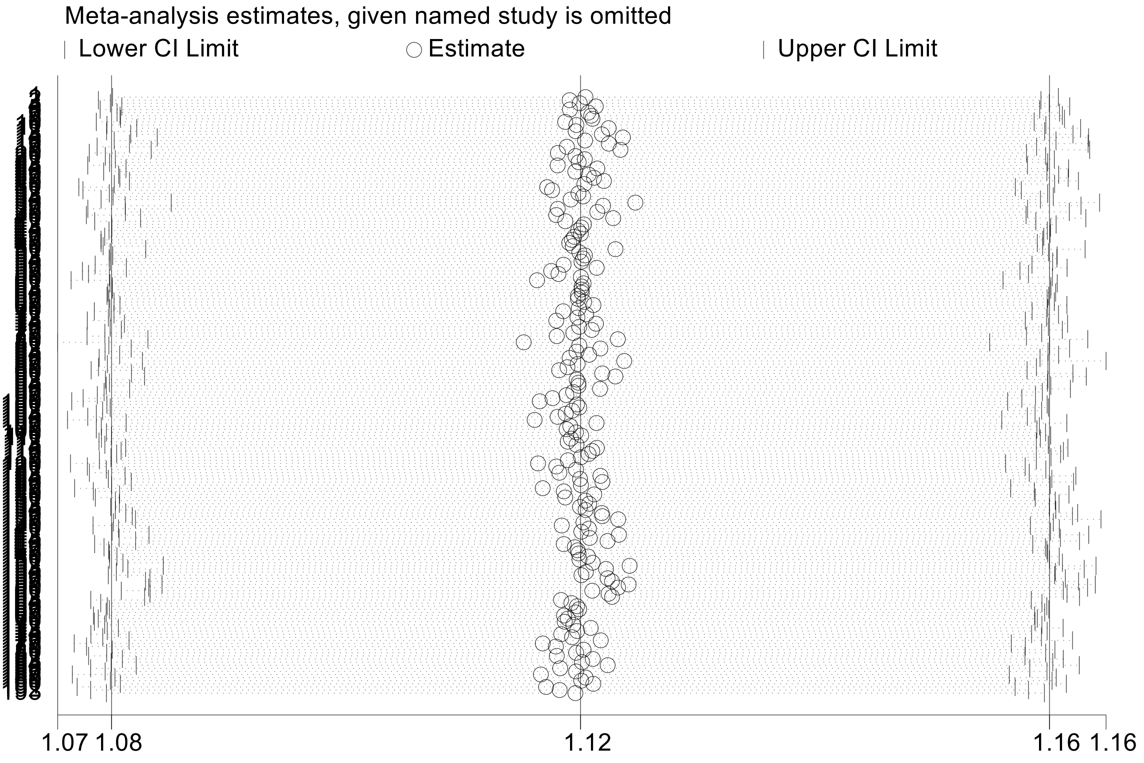

Supplement: Supplementary file 1 — Table S1: Quality assessment of the included studies using the Newcastle–Ottawa Quality Assessment Scale for cohort studies. Table S2: Quality assessment of the included studies using the Newcastle–Ottawa Quality Assessment Scale for case–control studies. Table S3: Results of Fixed/Random effect meta‐analysis to estimate pooled unadjusted ES (95% CI) for various cancer outcomes by trajectories. Table S4: Results of Random effect sub‐group analysis to estimate pooled unadjusted ES (95% CI) for various cancer outcomes by trajectories based on follow‐up duration (< 15 years vs. ≥ 15 years), BMI assessment method (measured vs. recalled), and geographic region. Figure S1: Forest plots for the pooled effect size (ES) of composite outcome of obesity related cancers for different BMI trajectories compared to stable normal weight trajectory: (A) ES of composite outcome of obesity related for normal to obesity trajectory; (B) ES of composite outcome of obesity related for normal to overweight trajectory (C) ES of composite outcome of obesity related for overweight to obesity trajectory. Figure S2: Forest plots for the pooled effect size (ES) of composite outcome of gastrointestinal cancers for different BMI trajectories compared to stable normal weight trajectory. Figure S3: Forest plots for the pooled effect size (ES) of composite outcome women related cancers for different BMI trajectories compared to stable normal weight trajectory. Figure S4: Forest plots for the pooled effect size (ES) of colorectal cancer for different BMI trajectories compared to stable normal weight trajectory. Figure S5: Forest plots for the pooled effect size (ES) of pancreatic cancer for different BMI trajectories compared to stable normal weight trajectory. S6. Forest plots for the pooled effect size (ES) of liver cancer for different BMI trajectories compared to stable normal weight trajectory. Figure S7: Forest plots for the pooled effect size (ES) of kidney cancer for different BMI trajectories [file OBR-27-e70114-s001.pdf]
